# Heterofunctional Cationic Polyester Dendrimers as Antibacterial Agents: The Role of Internal and External Charges

**DOI:** 10.1021/acs.biomac.5c01094

**Published:** 2025-08-05

**Authors:** Arunika Singh, Natalia Sanz del Olmo, Michael Malkoch

**Affiliations:** † Department of Fibre and Polymer Technology, KTH Royal Institute of Technology, 100 44 Stockholm, Sweden; ‡ University of Alcala, Faculty of Sciences, Department of Organic and Inorganic Chemistry, and Research Institute in Chemistry “Andrés M. Del Río” (IQAR), 28805 Madrid, Spain

## Abstract

Antimicrobial resistance is a global health crisis, necessitating novel antibacterial strategies. Polycations, particularly polyester dendrimers, are promising due to their structural precision and membrane-disruptive mechanisms. However, existing dendrimers lack versatility in charge distribution, limiting their antibacterial efficacy. Here, we report two families of cationic polyester dendrimers (up to the third generation) with either internal or combined internal and external charges compared with traditional bis-MPA dendrimers bearing only external cations. These heterofunctional dendrimers, built from AB_2_C monomers, carry up to 45 charges at the highest generation. Internal propargyl amines were introduced via CuAAC chemistry, while external β-alanine provided complementary charges in the second family. Dendrimers with both internal and external charges showed superior antibacterial activity against Gram-positive and Gram-negative bacteria while maintaining biocompatibility. The second-generation dendrimer (G2-(PA-NH_3_^+^)_9_-(β-Ala-NH_3_^+^)_12_) exhibited bacteriostatic and bactericidal activity at 10–21 μM and enhanced *Escherichia coli* sensitivity, with favorable biodegradation, highlighting their promise as antimicrobials where charge distribution is key for efficacy.

## Graphical abstract

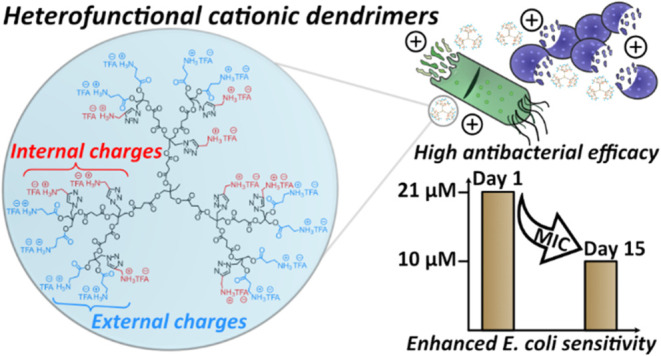

## Introduction

Antimicrobial resistance (AMR) has emerged as a pressing global health concern, with recent data suggesting a surge in antibiotic-resistant bacterial infections resulting in over 4.71 million deaths annually.^1^ Statistics also indicate that without any novel intervention, AMR could lead to more than 40 million deaths by 2050.^2^ The rise of AMR and multidrug-resistant (MDR) bacteria has rendered conventional antibiotics increasingly ineffective, necessitating the immediate need for alternative treatment strategies.^3^ While therapeutic alternatives such as bacteriophage therapy^4^ and combination therapies utilizing β-lactamase inhibitors^5^ show promise, their clinical utility remains constrained due to narrow-spectrum activity, bacterial resistance potential, immune clearance risks, and stringent handling requirements.^6,7^ To ensure clinical translation and long-term sustainability of antimicrobial therapeutics, there is a critical need for biodegradable antimicrobial systems capable of counteracting bacterial pathogens, as well as reducing resistance development. Highly charged polymers, particularly cationic dendrimers, have emerged as a promising approach due to their unique antibacterial mechanisms.^8,9^ They exert their antibacterial effects primarily through electrostatic interactions with negatively charged bacterial membranes, leading to membrane disruption.^10^ Unlike antibiotics that target specific enzymes or metabolic pathways, this nonspecific mechanism minimizes the likelihood of genetic resistance mutations, making cationic dendrimers promising candidates against AMR.

Dendrimers, a distinct subclass of dendritic polymers, are particularly appealing due to their monodispersity, nanometric size, and globular topology.^11–13^ These macromolecules consist of a central core, multiple branching units, and terminal functionalities and are synthesized in a precisely controlled manner following sequential growth steps, with each step increasing the dendritic generation, molecular weight, and terminal functionalities. PAMAM dendrimers,^14,15^ among the other well-known dendritic families, have been extensively researched for their antibacterial activity.^16–19^ However, their cytotoxicity remains a major concern due to the large number of surface cationic charges and slow degradation.^19,20^ To address these challenges, there is growing interest in biodegradable dendrimers as safer alternatives.

Polyester dendrimers have emerged as promising candidates in the biomedical field due to their facile synthesis, excellent biocompatibility, and biodegradability.^11,21,22^ Comparative studies have exemplified their superiority over PAMAM dendrimers in terms of cell viability and hydrolytic degradability at physiological conditions.^23^ Cationic polyester dendrimers based on 2,2-bis­(hydroxymethyl)­propionic acid (bis-MPA) have garnered significant attention for their promising antibacterial properties. One study has shown the synthesis and biological evaluation of bis-MPA dendrimers up to the fifth dendritic generation decorated with β-alanine functionalities in their periphery.^24^ The second-generation cationic β-alanine dendrimer, with 12 peripheral functionalities, exhibited the most promising biological performance with high antibacterial activity against *Escherichia coli* along with good biocompatibility in human cells. Additionally, a rapid degradation profile was observed for this derivative at physiological conditions (pH 7.4 and 37 °C) through hydrolysis of ester linkages connecting β-alanine chains. In another report, cysteamine functionalized bis-MPA dendrimers were shown as robust cationic systems demonstrating enhanced hydrolytic stability with potent antibacterial activity against Gram-positive bacteria (*Staphylococcus aureus*) and Gram-negative (*E. coli* and *Pseudomonas aeruginosa*) bacterial strains, and low toxicity at the concentrations they were active toward bacteria.^25^ These homofunctional dendrimers are characterized by the presence of the same chemical functionality in the dendritic periphery, which renders a dormant dendritic interior incapable of active functionalization with the desired chemical moieties.^22^ Despite the tremendous antibacterial efficacy and biocompatibility shown by homofunctional cationic constructs, their scope remains limited due to their terminal charge distribution. Their utility can be expanded through the design of more sophisticated dendritic scaffolds that enable the strategic incorporation of cationic ammonium groups throughout the entire framework rather than being confined to the periphery.

Heterofunctional polyester dendrimers (HFDs) have emerged as versatile nanocarriers with superior structural and chemical tunability over homofunctional scaffolds.^22,26^ Their distinct functional groups enable precise control over drug loading, release, and pharmacokinetics. Recent advances in monomers with orthogonal functionalities such as AB_2_C-type structures^27–29^ have further enhanced their chemoselective functionalization with additional cargo. Few studies have explored HFDs as polycationic agents for combating bacterial infections.^27,28,30^ A study demonstrated the synthesis of a second-generation HFD employing a bis-MPA-based AB_2_C monomer with 6 alkenes and 12 terminal ammonium groups.^30^ The alkene functionalities were cross-linked with a dithiol-functionalized PEG cross-linker via ultraviolet (UV)-cured thiol–ene coupling to form hydrogel networks, while the free ammonium groups conferred antibacterial activity against *E. coli* and *S. aureus* with good biocompatibility. In other reports, Boc-protected amino groups were selectively introduced into second-generation HFDs based on bis-MPA^27^ and 2-(bromomethyl)-2-(hydroxymethyl) propane-1,3 diol (BHP-diol)^28^ via CuAAC modification of azides. Trifluoroacetic acid (TFA) deprotection rendered 9 protonated charges for enabling further antibacterial evaluation.^27^ Although preliminary studies have shown promising results for the use of HFDs as polycationic systems, immense possibilities remain to fully exploit their chemical and biological potential for targeting aggressive bacterial infections.

In this work, we present a versatile synthetic approach toward the synthesis of two families of cationic polyester dendrimers based on a BHP-diol AB_2_C building block. The first dendritic family features internal ammonium functionalities derived from propargyl amine (PA-NH_3_^+^) along with peripheral hydroxyl groups, while the second family incorporates both internal and external ammonium functionalities, based on PA-NH_3_^+^ and β-alanine (β-Ala-NH_3_^+^), respectively. Cationic dendrimers from generation 1 to 3 (G1–G3) have been synthesized for each family, which display up to 21 and 45 amino groups, respectively. Biologically, the newly synthesized polycationic systems have been evaluated against Gram-positive and Gram-negative bacteria and screened for cytotoxicity in human keratinocytes (HaCaT) and human dermal fibroblast (hDF) cells. Importantly, the influence of commercial antibiotics and the test cationic dendrimer has been examined for bacterial resistance in both *S. aureus* and *E. coli* as models of Gram-positive and Gram-negative bacteria, respectively. Finally, the hydrolytic stability of the test antibacterial agent under physiological conditions has been established in both bacteria.

## Experimental Section

### Materials

All materials and chemical reagents were obtained from Sigma-Aldrich and utilized without further alterations unless specified otherwise. Dowex 50WX2 50–100 (H) was acquired from Acros Organics. Silica gel for column chromatography was purchased from ICN SiliTech (ICN Biomedicals GmbH, Eschwege, Germany). Amoxicillin and Penicillin G Sodium were purchased from Melford Biolaboratories Limited, United Kingdom.

*Escherichia coli* ATCC 25922 (*E. coli* ATCC 25922) and *S. aureus* ATCC 29213 (*S. aureus* ATCC 29213) were graciously supplied by the Karolinska Institute. *Pseudomonas aeruginosa* 22644 (*P. aeruginosa* 22644) was bought from DSMZ (Leibniz Institute DSMZ - German Collection of Microorganisms and Cell Cultures GmbH). Human dermal fibroblast (hDF) and Human epidermal keratinocyte (HaCaT) cells were obtained from the American Type Culture Collection (ATCC). For the evaluation of cell viability, Dulbecco’s modified Eagle medium (DMEM), fetal bovine serum (FBS), and a penicillin/streptomycin antibiotic mixture were procured from Thermo Fisher Scientific. For the assessment of antibacterial activity, Mueller Hinton Broth 2 (MHB II broth) and Mueller Hinton Agar were purchased from Sigma-Aldrich.

### Synthetic Protocols

All synthetic protocols are described in the Supporting Information.

### Characterization Methods

#### Nuclear Magnetic Resonance (NMR)

^1^H NMR, ^13^C NMR, and 2D-NMR analyses were conducted utilizing a Bruker AM NMR instrument. The ^1^H NMR spectra were recorded at 400 MHz after 16 scans at a spectral window of 20 ppm and a relaxation delay of 1 s, with automatic locking and shimming processes. The ^13^C NMR spectra were acquired at 101 MHz over a range of 256–1024 scans at a spectral window of 240 ppm and a relaxation delay of 2 s. The Diffusion-Ordered Spectroscopy NMR (DOSY-NMR) spectra were recorded with a spectral width of 12 ppm and a transmitter frequency offset set to 5 ppm, using 16 scans, a prescan delay of 6.5 microseconds, and a relaxation delay of 3 s, at a temperature of 25 °C. The resulting spectra were analyzed using MestReNova version 14.2.0–26256 (Mestrelab Research S.L, 2020).

#### Matrix-Assisted Laser Desorption/Ionization Time-of-Flight (MALDI-TOF) Mass Spectrometry

MALDI-TOF was conducted in a Bruker UltrafleXtreme MALDI-TOF mass spectrometer (Bruker Daltonics, Bremen, Germany) equipped with a SmartbeamII laser (355 nm, UV) operating in positive ion mode. The calibration was performed using SpheriCal calibrants (Polymer Factory Sweden AB). The mass spectra were recorded and processed by utilizing FlexControl and FlexAnalysis Version 3.4 (Bruker Daltonics). Trans-2-[3-(4-*tert*-Butylphenyl)-2-methyl-2-propenylidene]­malononitrile (DTCB) and 2,5-dihydroxybenzoic acid (DHB) were employed as matrices and dissolved in tetrahydrofuran at a concentration of 20 mg/mL. The MALDI sample preparation was performed by consecutively depositing 1 μL of a 1 mg/mL analyte solution and 2 μL of the matrix solution onto an MPT 284 Target ground steel TF Target from Bruker Daltonics. The spectra were acquired in reflector mode with an acceleration voltage of 25 kV and a reflector voltage of 26.3 kV with the laser intensity set between 50 and 100% to achieve high-resolution spectra.

#### Size Exclusion Chromatography (SEC)

SEC measurements were recorded and analyzed using a TOSOH EcoSECHLC-8320GPC instrument equipped with an EcoSEC RI detector and three columns (PSS PFG 5 μm; Microguard, 100, and 300 Å) from PSS GmbH, allowing for molecular weight resolution in the range of 300–100,000 Da. Dimethylformamide (DMF) was used as the mobile phase, with 0.01 M LiBr maintained at 35 °C. SEC samples were prepared for the postfunctionalized dendrimers within a concentration range of 2–3 mg/mL. The calibration was performed using narrow linear poly­(methyl methacrylate) standards sourced from PSS. Toluene was employed as an internal standard for correcting flow rate fluctuations. The data analysis and plotting of graphs were carried out in WinGPC Unity software version 7.2 and Origin 9.1.0 Sr1, respectively.

#### Fourier Transform Infrared Spectroscopy (FTIR)

FTIR spectra were recorded on a PerkinElmer FTIR spectrometer equipped with an ATR accessory. All samples were dried under vacuum and measured over the spectral range of 600–4000 cm^–1^ at a resolution of 4 cm^–1^ with 16 accumulated scans. Baseline correction and normalization were applied during data processing using PerkinElmer Spectrum IR software, version 10.5.1.

#### Antibacterial Activity Assessment of the Cationic Dendrimers in Planktonic Bacteria

Minimum inhibitory concentration (MIC) and minimum bactericidal concentration (MBC) assays were performed to evaluate the antibacterial properties of the synthesized cationic dendrimers against *E. coli*, *S. aureus*, and *P. aeruginosa*. For MIC determination, the test compounds were diluted with sterilized deionized (DI) water using a double dilution method. Bacterial solutions at log phase were diluted with MHB II broth to achieve a concentration of 10^6^ CFU mL^–1^. An equal volume (50 μL) of the adjusted microorganism concentration was incubated with the different dilutions of test compounds and controls in sterile 96-well plates. The plates were left to incubate for 18 h at 37 °C with shaking (250 rpm). The MIC values were then determined based on the final absorbance readings at OD_620_, which were measured using the Multiskan FC Microplate reader (Thermo Fisher Scientific, Shanghai). The negative controls consisted of the bacteria without the test compound and the culture medium without bacteria and test compound. Amoxicillin and Ciprofloxacin were utilized as positive controls.

For MBC determination, 10 μL of broth from the wells at MIC, 2× MIC, 4× MIC, and 8× MIC, along with controls, were subcultured onto fresh agar plates and incubated overnight to check for bacterial growth the following day. All microbiological evaluations were performed in triplicate (*n* = 3) using freshly prepared dendritic and antibiotic stock solutions for each experiment.

#### Evaluation of Antimicrobial Resistance

The procedure for the determination of MIC was performed for *S. aureus* and *E. coli* in the same manner as described above, and the MIC values obtained after 18 h were assigned as the initial minimum inhibitory concentrations (MIC_day1_) for the test compound and commercial antibiotics, respectively in both bacterial strains. Next, 100 μL was taken from the well corresponding to the concentration below the MIC and transferred to 2 mL of broth for a 4 h incubation period, accompanied by shaking. The obtained suspension was then adjusted to a concentration of 10^6^ CFU mL^–1^ and added to the plate containing various dilutions of the test compound and antibiotics as described previously. The MIC values were monitored daily over a period of 15 days, resulting in MIC_day15_. The relative MIC value (MIC_day15_/MIC_day1_) was calculated by taking the ratio of the MIC obtained from the 15th subculture (MIC_15_) to that obtained from the first culture (MIC_day1_).

#### Hydrolytic Stability Evaluation

Two stock solutions of the test compound were prepared in sterilized DI water (pH 6.5) and phosphate-buffered saline (PBS) (pH 7.4) at a concentration of 4 mg mL^–1^, respectively, and were kept at 37 °C. The double dilution method was performed in sterilized DI water and PBS separately, for the aliquots taken from the respective stock solutions. The MIC experiment was conducted in the same manner as described earlier for both stock solutions at different time intervals (0, 3, 7, 30, and 96 h) in *E. coli* and *S. aureus*, respectively. The MIC values were recorded after an 18 h incubation period starting from the designated time interval.

#### Cytotoxicity Evaluation of the Cationic Dendrimers

The cytotoxicity of the synthesized cationic dendrimers was assessed by using the Alamar Blue assay. HaCaT and hDF cell lines were cultured in DMEM supplemented with 10% FBS and 100 units mL^–1^ each of penicillin and streptomycin under 5% CO_2_ at 37 °C. Both the cell lines were detached with trypsin and were subsequently seeded in 96-well plates at a density of 5 × 10^4^ cells per 100 μL of DMEM and incubated overnight. The following day, the medium was replaced with fresh DMEM, and the cells were treated with reference and test compounds diluted in DMEM at varying concentrations (62 to 1000 μg mL^–1^), with each concentration added to three wells of the plate. After 24 h, the treated wells were replaced with 100 μL of a 90:10 mixture of fresh DMEM and Alamar Blue reagent, followed by a 4 h incubation period. Next, the fluorescence intensities were measured using a Tecan Infinite M200 Pro plate reader at excitation/emission wavelengths of 560/590 nm. The cytotoxicity assessments were conducted in triplicate (*n* = 3) with freshly prepared dendritic stock solutions for each experiment.

## Results and Discussion

### Synthesis of Heterofunctional Cationic Polyester Dendrimers

Despite traditional bis-MPA cationic dendrimers^24,25^ showing potential as effective antibacterial agents, they are inherently limited by their homofunctional architecture, where the location of charge distribution is confined to the periphery (Figure 1A). This structural limitation restricts their chemical versatility and leaves the dendritic interior dormant, preventing full exploitation of the scaffold by introducing additional cationic charges. The newly synthesized cationic dendrimers incorporate amino groups both internally and peripherally (Figure 1B), thereby enabling a substantial increase in the charge density. Beyond enabling precise control over charge localization, the new cationic HFDs also offer greater versatility in tuning the number of protonated ammonium groups compared to traditional bis-MPA dendrimers. The first family with a comparatively lesser number of modifiable internal groups yields a lower number of cationic charges, while the second family incorporating both internal and external cationic charges renders a polycationic system with a significantly higher number of ammonium groups when compared to the cationic bis-MPA dendrimers (Figure 1C). Additionally, a comparison between the cationic charge densities of the first and second families of dendrimers reveals an interesting trend. As seen in Figure 1C, the first-generation dendrimer with internal and external cationic charges exhibits the same number of charges (9 charges) as a second-generation dendrimer with only internal cationic charges. This pattern persists across generations and enables the synthesis of lower-generation dendrimers (2nd family) that can attain the same charge density as higher-generation dendrimers from the first family. It is important to highlight that, despite the presence of triazole groups in both families of dendrimers that are susceptible to protonation, their low p*K*_a_ values (1–2) cause them to remain largely deprotonated at physiological pH (∼7.4).^31–33^ Thus, their contribution to the overall positive charge of the cationic HFDs is minimal compared to that of the protonated primary amines.

**Figure 1:**
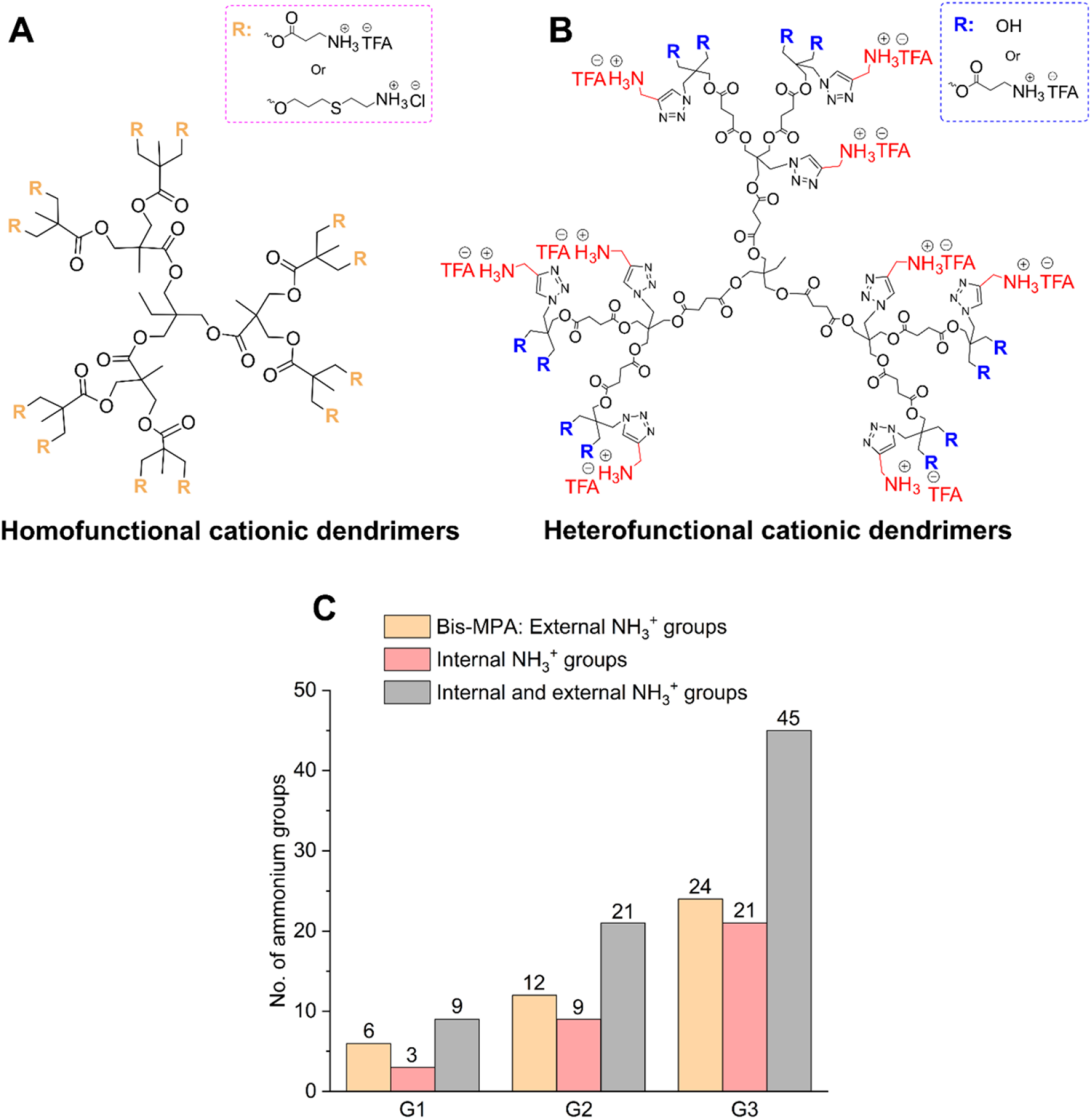
Comparison of second-generation cationic dendritic scaffolds with (A) homofunctional configuration showing peripheral β-Ala-NH_3_^+^ ^24^ or cysteamine^25^ groups and (B) heterofunctional configuration exhibiting internal PA-NH_3_^+^ groups and external OH or β-Ala-NH_3_^+^ functionalities. (C) An overview of the number of cationic ammonium groups presented by traditional bis-MPA dendrimers^24,25^ and the newly synthesized dendrimers (1st and 2nd family) from generation 1 to 3 under physiological conditions (pH = 7.4).

To address the limitations of traditional bis-MPA cationic scaffolds, a synthetic route (Scheme 1) was developed with the objective of attaining two classes of heterofunctional polyester dendritic scaffolds, which display cationic ammonium groups spread out internally (1st family) and in both the interior as well as exterior (2nd family), respectively. The BHP-diol-based AB_2_C monomer^28^ utilized to construct these polyester frameworks allowed the inclusion of active internal ammonium groups in the dendritic interior for overcoming the peripheral charge restriction of bis-MPA scaffolds. Additionally, the simultaneous introduction of cationic charges in the dendritic interior as well as exterior aided in sizably increasing the number of charges as opposed to those of the bis-MPA counterparts, thereby fully optimizing the potential of these next-generation cationic dendritic scaffolds.

**Scheme 1:**
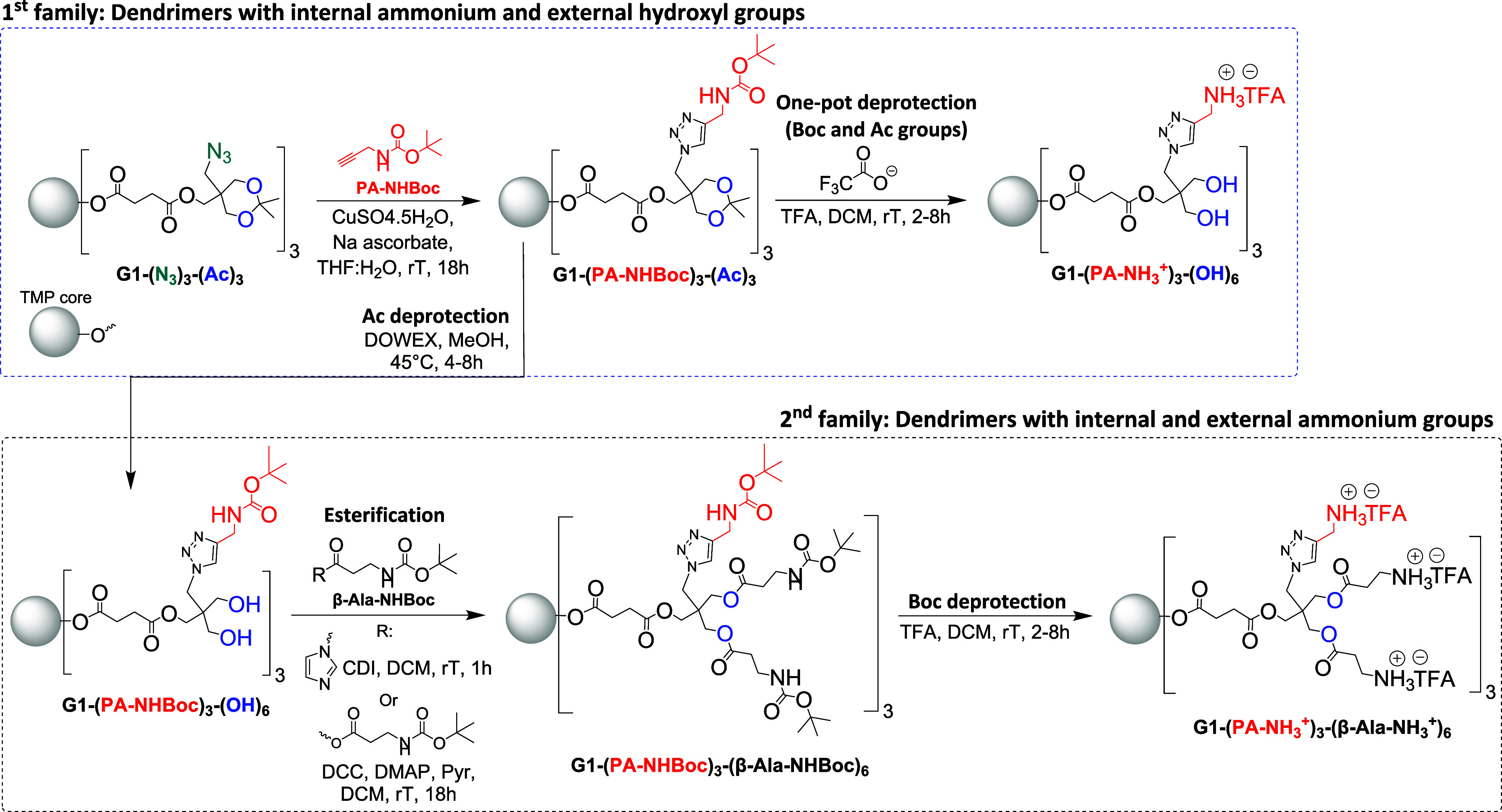
Overview of the Synthetic Strategy towards Two Families of Cationic Dendrimers, Using First-Generation Derivatives as Examples

For clarity in the nomenclature of the synthesized dendrimers discussed in the following sections, the formula G-(internal groups)*_m_*-(external groups)*_n_* will be used. Here, G denotes the dendrimer generation, internal groups refer to either Boc-protected or deprotected propargyl amine functionalities (PA-NHBoc or PA-NH_3_^+^), and external groups correspond to peripheral hydroxyl groups and Boc-protected or deprotected β-alanine functionalities (β-Ala-NHBoc or β-Ala-NH_3_^+^). The subscripts “*m*” and “*n*” indicate the number of internal and external functional groups, respectively. Additionally, the abbreviation for trimethylolpropane (TMP) has been omitted from the nomenclature as the dendritic core remains consistent across both synthesized dendrimer families. The synthesis toward the first family displaying internal ammonium groups and peripheral hydroxyl functionalities involved sequential steps comprising postfunctionalization through CuAAC,^28^ followed by one-pot TFA-mediated deprotection reactions. Initially, the acetonide-protected (Ac) dendritic precursors possessing internal azide groups from generation 1 to 3 were postfunctionalized with PA-NHBoc^28^ via CuAAC. This reaction was run overnight in a 1:1 ratio tetrahydrofuran/water (THF/H_2_O) solvent mixture, utilizing copper sulfate pentahydrate (CuSO_4_·5H_2_O) as a catalyst and sodium ascorbate (Na ascorbate) as a reducing agent.^28^ Subsequently, the purification, which consisted of washes with a 0.5% w/w EDTA solution followed by flushing the crude product through silica plugs, facilitated the removal of CuSO_4_·5H_2_O, Na ascorbate, and excess PA-NHBoc. The pure PA-NHBoc functionalized intermediates (G1-(PA-NHBoc)_3_-(Ac)_3_, G2-(PA-NHBoc)_9_-(Ac)_6_,^28^ and G3-(PA-NHBoc)_21_-(Ac)_12_) were obtained as colorless oils in near quantitative yields. Next, the obtained PA-NHBoc precursors (G1–G3) were exposed to TFA in the presence of DCM as a cosolvent for 2–8 h (varying reaction time for each generation) to facilitate one-pot deprotection of Boc and Ac groups, in a single reaction step. The crude products were precipitated in ether to ultimately yield the final dendrimers with internally charged ammonium groups (TFA as a counteranion) and external hydroxyl functionalities (G1-(PA-NH_3_^+^)_3_-(OH)_6_, G2-(PA-NH_3_^+^)_9_-(OH)_12_ and G3-(PA-NH_3_^+^)_21_-(OH)_24_).

The synthesis of the second family exhibiting internal and external ammonium groups involved three reaction steps constituting DOWEX deprotection, esterification^24,34^ and TFA deprotection. The first reaction step began from the PA-NHBoc functionalized derivatives where the Ac groups were deprotected with an acidic DOWEX resin in methanol (MeOH) at 45**°**C, followed by filtration of DOWEX and concentration of MeOH to yield dendritic precursors with peripheral hydroxyl groups (G1-(PA-NHBoc)_3_-(OH)_6_, G2-(PA-NHBoc)_9_-(OH)_12_ and G3-(PA-NHBoc)_21_-(OH)_24_) as slightly pink oils in moderate yields. Subsequently, the external hydroxyl groups underwent esterification with β-Ala-NHBoc for further introducing amino-based functionalities in the dendritic exterior. This was achieved using Fluoride Promoted Esterification (FPE) chemistry, wherein the β-Ala-NHBoc was first reacted with *N*,*N*′-carbonyldiimidazole (CDI) for 1 h at room temperature to obtain the imidazole-activated β-Ala-NHBoc.^24,34^ The CDI-activated β-Ala-NHBoc was then reacted *in situ* with hydroxyl precursors overnight in the presence of cesium fluoride (CsF), which served as a mild inorganic base for the formation of highly nucleophilic alkoxide ions and to facilitate the esterification reaction. The purification of G1-(PA-NHBoc)_3_-(β-Ala-NHBoc)_6_ and G2-(PA-NHBoc)_9_-(β-Ala-NHBoc)_12_ was accompanied by washes with sodium bisulfate (NaHSO_4_) and sodium bicarbonate (NaHCO_3_), following precipitations in ether. The products were obtained as colorless oils with decent yields. The third-generation dendrimer (G3-(PA-NHBoc)_21_-(β-Ala-NHBoc)_24_) was synthesized via anhydride chemistry as the complete functionalization by FPE required a higher molar ratio of imidazole-activated β-Ala-NHBoc. The anhydride-based esterification was conducted using the β-Ala-NHBoc anhydride^24,34^ formed from *N*,*N*′-dicyclohexylcarbodiimide (DCC), coupling with hydroxyls overnight in the presence of catalytic amount of 2-(dimethylamino)­pyridine (DMAP) and an excess of pyridine. After precipitation in ether, G3-(PA-NHBoc)_21_-(β-Ala-NHBoc)_24_ was obtained in poor yields (37%), which was almost less than half the synthesized yields of the first and second-generation derivatives. Finally, in the last reaction step, the external as well as internal Boc groups from both β-alanine and PA were deprotected with TFA, following the same reaction conditions as seen for the one-pot deprotection to obtain the second family (G1-(PA-NH_3_^+^)_3_-(β-Ala-NH_3_^+^)_6_, G2-(PA-NH_3_^+^)_9_-(β-Ala-NH_3_^+^)_12_ and G3-(PA-NH_3_^+^)_21_-(β-Ala-NH_3_^+^)_24_) as colorless oils. The synthesis of the third-generation β-alanine derivatives proved to be quite tedious requiring large amounts of the β-Ala-NHBoc monomer for complete functionalization and yielding low final product recovery after purification.

All the synthetic reactions depicted in Scheme 1 were followed through a combination of characterization techniques consisting of NMR spectroscopy (Figure 2A), MALDI-TOF (Figure 2B), FTIR (Figures S17, S18) and SEC (Figures S19, S21 and S24).

**Figure 2:**
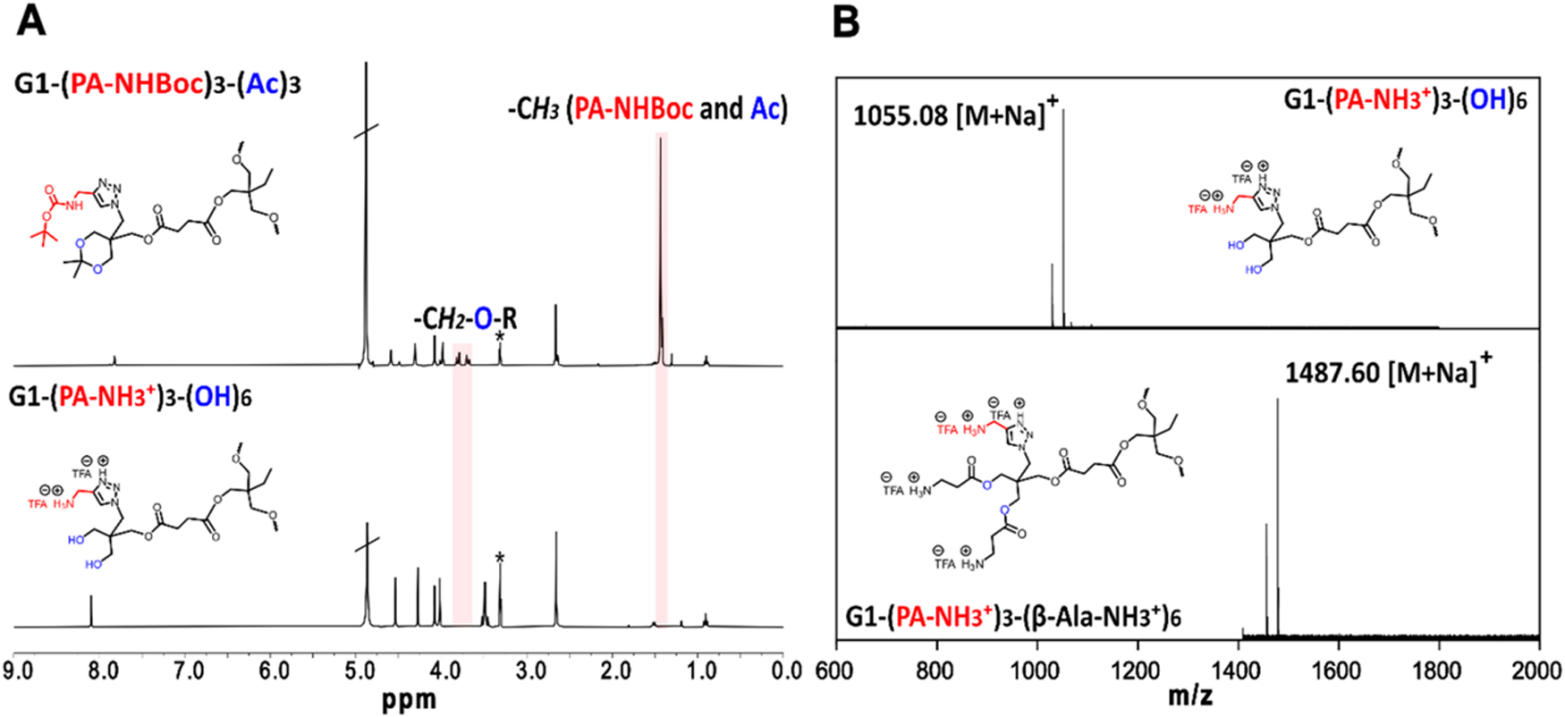
(A) Stacked ^1^H NMR spectra of G1-(PA-NHBoc)_3_-(Ac)_3_ and G1-(PA-NH_3_^+^)_3_-(OH)_6_ in CD_3_OD (*). (B) Stacked MALDI-TOF spectra of G1-(PA-NH_3_^+^)_3_-(OH)_6_ and G1-(PA-NH_3_^+^)_3_-(β-Ala-NH_3_^+^)_6_ (bottom) in DHB.

The successful postfunctionalization of azide-based precursors with PA-NHBoc via CuAAC was confirmed by the disappearance of the characteristic azide stretch at 2103 cm^–1^ in the FTIR spectrum, as seen in previous report.^28^ Figure 2A indicates the successful completion of the TFA-mediated one-pot deprotection of the first-generation PA-NHBoc intermediate through ^1^H NMR, where the peak attributing to the protons of methyl groups present in both Boc and acetonides at 1.43 ppm completely disappears. This was also accompanied by the disappearance of the signal attributed to the protons of methylene moieties next to the acetonide groups at 3.67 and 3.79 ppm and the appearance of a new signal attributed to the methylene groups next to the hydroxyl functionalities at 3.49 ppm. Additionally, under acidic conditions such as those provided by TFA, the proton signal corresponding to the triazole ring shifts downfield from 7.82 ppm in the Boc-protected precursor to 8.09 ppm in the deprotected cationic derivative, indicating protonation of the triazole groups. While such protonation is evident under acidic environments and in deuterated solvents such as CD_3_OD, the dendritic structures and charge numbers depicted in Figure 1B,1C and Scheme 1 are intended to reflect the structural charge state at physiological pH conditions (∼7.4), where the triazole groups (p*K*_a_ ∼ 1–2) remain largely deprotonated and therefore contribute minimally to the overall cationic charge density.^31–33^ The TFA deprotection of Boc groups in the second dendritic family was also verified by NMR spectroscopy in the same manner as that described above. The molecular weights of the obtained cationic polyester scaffolds were corroborated with MALDI-TOF as shown in Figure 2B.

All the PA-NHBoc intermediates showing peripheral Ac groups and hydroxyl functionalities, along with β-Ala-NHBoc functionalized derivatives, have been fully characterized by NMR (^1^H, ^13^C) spectroscopy, MALDI-TOF and SEC to verify their purity, structural perfection, and monodispersity. The structural characterization of the first and second families of cationic dendrimers was only possible with NMR spectroscopy and MALDI-TOF. SEC analysis of the synthesized cationic dendrimers was not feasible, likely due to aggregation and accumulation within the filter during the filtration process preventing analyte detection. Furthermore, the molecular weight (MALDI-TOF) and polydispersity analysis (SEC) of the third-generation β-alanine derivatives (G3-(PA-NHBoc)_21_-(β-Ala-NHBoc)_24_ and G3-(PA-NH_3_^+^)_21_-(β-Ala-NH_3_^+^)_24_) proved to be challenging despite testing various MALDI matrices and optimizing sample preparation in DMF at different concentrations. Therefore, these β-alanine derivatives were primarily characterized by NMR spectroscopy and supplemented by DOSY to verify the diffusion coefficients from a single species and confirm their complete functionalization after esterification and TFA deprotection reactions. A more detailed description of the synthetic protocols and characterization methods, including ^1^H NMR, ^13^C NMR, MALDI, SEC and FTIR is provided in the Supporting Information.

### Antibacterial Activity and Cytotoxicity Evaluation

To elucidate the impact of location of cationic charges, as well as charge density, on the antibacterial efficacy, we evaluated two families of amino-based polyester dendrimers by determining their minimum inhibitory concentration (MIC) and minimum bactericidal concentration (MBC) against Gram-positive bacteria (*S. aureus*) and Gram-negative (*E. coli* and *P. aeruginosa*) bacterial model strains. Additionally, to better understand the role of inner positive charges on antibacterial performance compared to peripheral charges, a second-generation homofunctional bis-MPA dendrimer with peripheral cationic β-alanine groups (G2-(β-Ala-NH_3_^+^)_12_) was selected.^24,34^ The MIC and MBC values for both the families of cationic dendrimers are displayed in Figure 3A.

**Figure 3:**
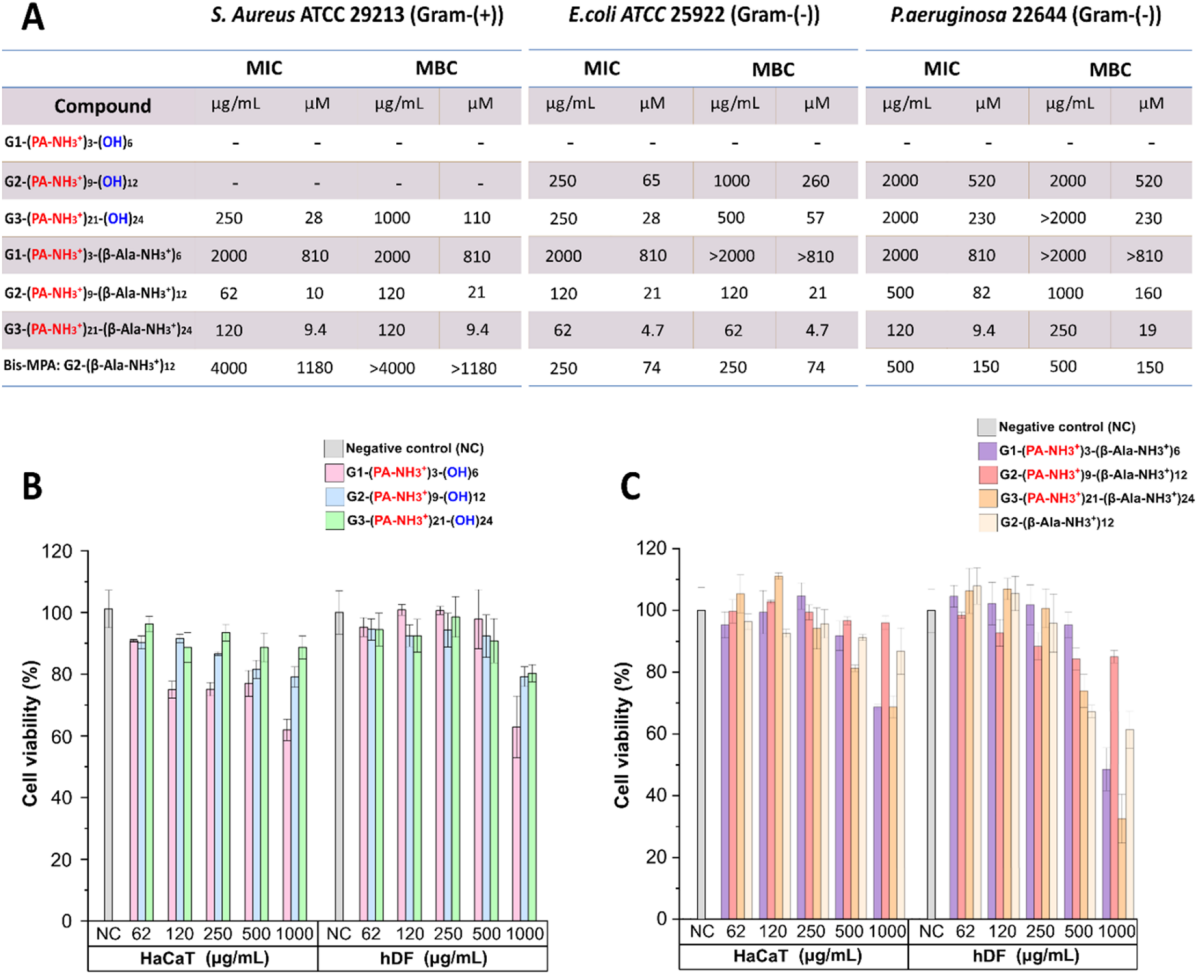
(A) MIC and MBC values of the newly synthesized families of cationic polyester dendrimers along with the second-generation bis-MPA dendrimer functionalized with β-alanine (G2-(β-Ala-NH_3_^+^)_12_)^24,34^ after evaluation in Gram-positive (*S. aureus*) and Gram-negative (*E. coli* and *P. aeruginosa*) bacterial strains. (B, C) Cytotoxicity evaluation of the (B) 1st family and (C) 2nd family of cationic dendrimers along with G2-(β-Ala-NH_3_^+^)_12_ ^24,34^ after 24 h incubation with HaCaT and hDF cell lines at various concentrations (62–1000 μg/mL) by Alamar Blue assay. Mean values are shown with error bars showing standard deviation, *n* = 3.

It was observed that among the first family containing positive charges only in the interior of the skeleton, just the third-generation dendrimer with 21 internal charges (G3-(PA-NH_3_^+^)_21_-(OH)_24_) could exhibit any significant antibacterial activity in both *S. aureus* and *E. coli* with MIC values of 28 μM and MBC values of 110 and 57 μM, respectively. In contrast, the second family displaying internal and external charges showed a drastic difference with regard to their antibacterial performance with each increasing generation. Noticeably, the second-generation (G2-(PA-NH_3_^+^)_9_-(β-Ala-NH_3_^+^)_12_) and third-generation (G3-(PA-NH_3_^+^)_21_-(β-Ala-NH_3_^+^)_24_) dendrimers exhibited significant bacteriostatic and bactericidal activity in both *S. aureus* and *E. coli* at low concentrations. Among all synthesized dendritic constructs, G3-(PA-NH_3_^+^)_21_-(β-Ala-NH_3_^+^)_24_ demonstrated the highest antibacterial potency against all of the tested bacteria, including *P. aeruginosa* with an MIC value of 9.4 μM and an MBC value of 19 μM.

The antibacterial performance displayed by both families of cationic dendrimers highlights key insights into the role played by the location and number of cationic ammonium groups. First, the relatively low antibacterial activity of dendrimers with internal cationic charges can be attributed to the limited accessibility of these ammonium groups toward the bacterial membrane, which are shielded by the branched dendritic structure. Second, the incorporation of peripheral cationic charges in the second family significantly boosted their antibacterial potency, likely due to the improved accessibility of the extended β-alanine chains facilitating membrane disruption. Lastly, the antimicrobial efficacy of the cationic dendrimers was more pronounced when the total number of charges reached or exceeded 21, indicating a direct correlation between the charge density and antibacterial activity. A particularly remarkable difference in antibacterial performance was observed when comparing the third-generation dendrimer from the first family (G3-(PA-NH_3_^+^)_21_-(OH)_24_) and the second-generation dendrimer from the second family (G2-(PA-NH_3_^+^)_9_-(β-Ala-NH_3_^+^)_12_), both of which contain 21 cationic charges in total. In *S. aureus*, G2-(PA-NH_3_^+^)_9_-(β-Ala-NH_3_^+^)_12_ exhibited an MIC value nearly 3.0 times lower and MBC value 5.5 times lower than that of G3-(PA-NH_3_^+^)_21_-(OH)_24_. Similar trends were observed with regard to their performance against *E. coli* and *P. aeruginosa*. These marked differences between both families reinstate the importance of charge localization in attaining antibacterial efficacy. Furthermore, G2-(PA-NH_3_^+^)_9_-(β-Ala-NH_3_^+^)_12_ shares commonality with the bis-MPA dendrimer G2-(β-Ala-NH_3_^+^)_12_,^24,34^ as both possess 12 β-alanine chains at the periphery. However, G2-(PA-NH_3_^+^)_9_-(β-Ala-NH_3_^+^)_12_ significantly outperformed the traditional bis-MPA construct in terms of antibacterial activity across all tested bacterial strains. This enhanced performance can be attributed to the heterofunctional polyester scaffold, which accommodates nine additional internal ammonium groups within the same dendritic framework. The increased number of charges resulting from this configuration strategically aids in the optimization of antimicrobial activity.

Despite the favorable antibacterial properties offered by positively charged systems, their major drawback is associated with the inherent toxicity for the purpose of biomedical applications.^19,20^ This phenomenon is caused due to the interaction of surface cationic groups with negatively charged biological membranes *in vivo*.^10^ To gain an in-depth understanding of the toxicity profile of the newly synthesized cationic dendrimers, a thorough cytotoxicity screening was performed in HaCaT and hDF cell lines by testing concentrations ranging from 62 to 1000 μg/mL based on the MIC and MBC values obtained in the microbiological evaluation, after incubation for 24 h. The cytotoxicity of the second family of cationic dendrimers was evaluated in comparison to G2-(β-Ala-NH_3_^+^)_12_ ^24,34^ which is the closest in terms of structure with peripheral β-Ala-NH_3_^+^ groups. Figure 3B,C represents the cell viability results obtained for both families of cationic dendrimers. Overall, both families of the newly synthesized cationic polyester dendrimers exhibit excellent biocompatibility, with respect to the concentrations at which they show antibacterial activity.

The first family of dendrimers with internal cationic charges showed good biocompatibility in both the cell lines at the tested concentration range except for G1-(PA-NH_3_^+^)_3_-(OH)_6_ which became toxic at the highest concentration of 1000 μg/mL. The toxicity observed at this particular concentration seems reasonable for the first-generation dendrimer considering that its concentration in μM is significantly higher (730 μM) than the successive dendritic generations with increasing molecular weights (G2:260 μM and G3:110 μM). The second family of cationic dendrimers also exhibited satisfactory cell viability results in both the cell lines apart from G1-(PA-NH_3_^+^)_3_-(β-Ala-NH_3_)_6_ and G3-(PA-NH_3_^+^)_21_-(β-Ala-NH_3_)_24_ which became toxic at the highest concentration (1000 μg/mL). Interestingly, there was a drastic difference in their toxicity profiles compared with G2-(PA-NH_3_^+^)_9_-(β-Ala-NH_3_^+^)_12_. The second-generation β-alanine derivative demonstrated high cell viability of 96 and 85% in HaCaT and hDF cell lines, respectively, while the cytotoxicity of G1-(PA-NH_3_^+^)_3_-(β-Ala-NH_3_^+^)_6_ and G3-(PA-NH_3_^+^)_21_-(β-Ala-NH_3_^+^)_24_ declined much below the 70% acceptable range.^35^ This cytotoxicity could be attributed to the exceptionally high concentration of G1-(PA-NH_3_^+^)_3_-(β-Ala-NH_3_^+^)_6_ (400 μM) tested and the huge surface cationic charge density of G3-(PA-NH_3_^+^)_21_-(β-Ala-NH_3_^+^)_24_ (76 μM) owing to 45 ammonium groups, which are five times more than the first-generation and approximately twice that of the second-generation dendrimer. The high biocompatibility of G2-(PA-NH_3_^+^)_9_-(β-Ala-NH_3_^+^)_12_ at 160 μM may be attributed to an optimal tested concentration range falling between those of the first- and third-generation dendrimers as well as its lower charge density (21 charges) compared to the higher-generation dendrimer (45 charges). Higher-generation dendrimers, such as G3-(PA-NH_3_^+^)_21_-(β-Ala-NH_3_^+^)_24_, exhibit greater multivalence, which can lead to strong interactions with the cell membranes, potentially causing cytotoxicity.^36^ This highlights the importance of achieving an optimal balance between the concentration and cationic ammonium groups in antibacterial dendrimers to ensure their translation for nanomedicine applications. Additionally, the biocompatibility of the traditional cationic dendrimer G2-(β-Ala-NH_3_^+^)_12_ ^24,34^ was also within the acceptable range at all tested concentrations in HaCaT, while it became toxic at high concentrations of 500 μg/mL (150 μM) and 1000 μg/mL (300 μM) in the hDF cell line.

Among the tested candidates, G2-(PA-NH_3_^+^)_9_-(β-Ala-NH_3_^+^)_12_ and G3-(PA-NH_3_^+^)_21_-(β-Ala-NH_3_^+^)_24_ emerged as the most promising antibacterial agents, with the lowest values of MIC/MBC and exhibiting no toxicity at the concentrations effective against bacteria. While the MIC/MBC values for the third-generation dendrimer were the most promising, in terms of bacteriostatic activity, both the second- and third-generation β-alanine dendrimers showed similar MIC values (∼10 μM) against Gram-positive bacteria (*S. aureus*). Furthermore, G3-(PA-NH_3_^+^)_21_-(β-Ala-NH_3_^+^)_24_ demonstrated superior antibacterial activity against Gram-negative bacteria (*E. coli* and *P. aeruginosa*), and it also exhibited higher cytotoxicity at the highest concentrations. Given these factors, along with the synthetic challenges and lower yields associated with G3-(PA-NH_3_^+^)_21_-(β-Ala-NH_3_^+^)_24_, the second-generation dendrimer G2-(PA-NH_3_^+^)_9_-(β-Ala-NH_3_^+^)_12_ was selected for further evaluation of antibiotic resistance and hydrolytic stability. The synthesis of G2-(PA-NH_3_^+^)_9_-(β-Ala-NH_3_^+^)_12_ provided advantages in terms of scalability, higher yields, and reduced production time, making it a more favorable candidate for future clinical translation.

### Evaluation of Antibiotic Resistance

Antibiotic-resistant bacteria pose a major threat to the global health of the society which leads to a high mortality rate and prolonged illnesses in patients.^2^ One of the major challenges as a consequence of this phenomenon is the reduced effectiveness of the conventional antibiotic regime over prolonged periods. Cationic dendrimers have emerged as a promising strategy to combat AMR due to their ability to strongly interact with negatively charged bacterial membranes via multivalent electrostatic interactions, leading to enhanced membrane permeability, disruption, and bacterial lysis.^10^ This mechanism minimizes resistance development by targeting essential bacterial structures rather than specific biochemical pathways, making adaptation more challenging for bacteria. Additionally, they are known to mimic antimicrobial peptides to effectively target both Gram-positive and Gram-negative bacteria, including multidrug-resistant strains. In this study, G2-(PA-NH_3_^+^)_9_-(β-Ala-NH_3_^+^)_12_ from the second family was selected as the test cationic antibacterial agent to evaluate its effect on the resistance of Gram-positive and Gram-negative bacterial strains. S. aureus was chosen as the Gram-positive model, while *E. coli* was selected to represent Gram-negative bacteria due to the significantly higher efficacy of these dendrimers against it compared with *P. aeruginosa*. Commercial antibiotics such as Penicillin G and Amoxicillin, were also included in this study for comparison as they are known to be effective against various strains of *S. aureus* and *E. coli*, respectively, but have also shown to build resistance in them over repeated administration.^37–41^

The bacterial resistance assessment was carried out by measuring the MIC repeatedly on a daily basis over 15 cycles, using subcultures derived from the MIC determined in the previous cycle.^42^ The comparison of the MIC values obtained on the final day (MIC_day15_) with those on the first day (MIC_day1_) aided in comprehending the changes in the inhibitory efficacy of G2-(PA-NH_3_^+^)_9_-(β-Ala-NH_3_^+^)_12_ and the commercial antibiotics over the course of the experiment. As it can be seen in Figure 4A, *S. aureus* grew highly resistant to G2-(PA-NH_3_^+^)_9_-(β-Ala-NH_3_^+^)_12_ within 5 days, requiring a 32 times higher concentration (330 μM) than the original dose (10 μM) to observe bacterial inhibition, while the commercial antibiotic Penicillin G had a significantly lower tendency to induce bacterial resistance. As *S. aureus* required considerably high doses of G2-(PA-NH_3_^+^)_9_-(β-Ala-NH_3_^+^)_12_ (>2000 μg/mL) to achieve bacteriostatic effect in a matter of 5 days, the cyclic experiment was stopped as such large concentrations of treatment were beyond the scope for biological testing. In contrast, this is the case for *E. coli*, the commercial antibiotic Amoxicillin induced resistance, as evidenced by a 4-fold increase in MIC when comparing the 15th day and first day, whereas G2-(PA-NH_3_^+^)_9_-(β-Ala-NH_3_^+^)_12_ enhanced the susceptibility of the Gram-negative bacterial strain, reducing the MIC value by half by the final day compared to the initial measurement (Figure 4B).

**Figure 4:**
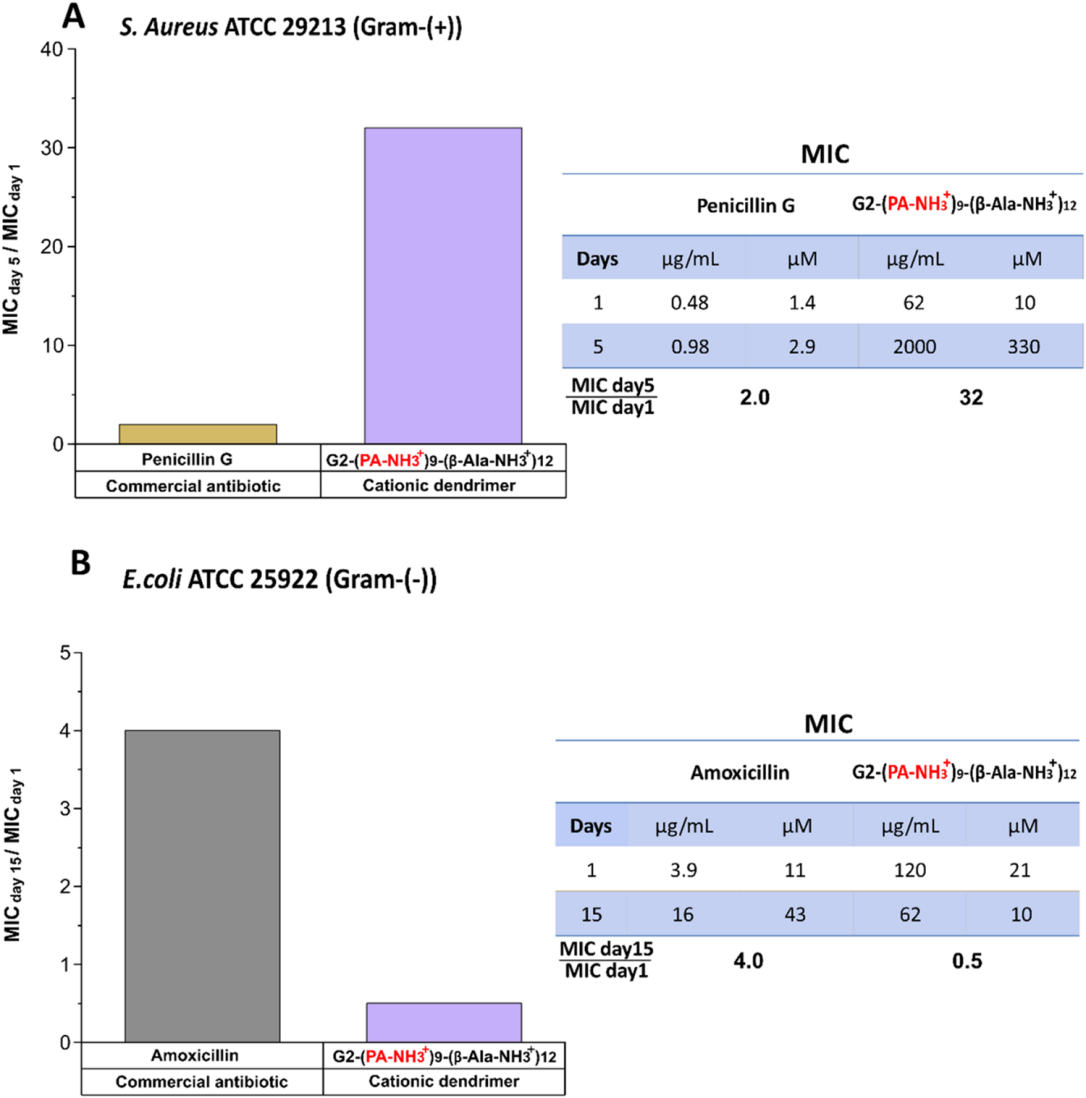
Assessment of antibiotic resistance in (A) Gram-positive bacteria: *S. aureus* using Penicillin G as the commercial antibiotic and G2-(PA-NH_3_^+^)_9_-(β-Ala-NH_3_^+^)_12_ as the antibacterial cationic dendrimer. MIC_day5_/MIC_day1_ is the ratio of the MIC from the 5th subculture to the 1st subculture. (B) Gram-negative bacteria: *E. coli* using Amoxicillin as the commercial antibiotic and G2-(PA-NH_3_^+^)_9_-(β-Ala-NH_3_^+^)_12_ as the antibacterial cationic dendrimer. MIC_day15_/MIC_day1_ is the ratio of MIC obtained from the 15th subculture to the 1st subculture.

These results demonstrate that even though the second-generation dendrimer exhibited stronger antimicrobial activity against *S. aureus* than *E. coli* as a standalone treatment, long-term resistance profiling revealed a contrasting trend where *E. coli* developed sensitivity to the second-generation cationic β-alanine dendrimer, whereas *S. aureus* developed a high resistance following repeated cyclic exposure. These distinct resistance profiles could arise due to multiple factors. First, the mechanisms of antibiotic resistance development differ significantly between *S. aureus* and *E. coli* due to their structural and genetic variations.^43,44^ Second, both species comprise more than hundreds of strains, each of them with different antibiotic resistance profiles and genomic content.^45–48^ Since this study evaluated only a single strain of each bacterium, the results highlight how prolonged exposure can influence the evolution of resistance or sensitivity in a specific strain to the selected antibacterial treatment.

While numerous studies have investigated the bacteriostatic and bactericidal activity of cationic dendrimers against various planktonic bacterial species, their long-term cyclic exposure and potential to induce sensitivity or resistance have remained largely unexplored. Few studies^49^ that have assessed resistance development in Gram-positive and Gram-negative bacteria following prolonged cyclic exposure to cationic dendrimers have shown lower resistance compared to conventional antibiotics. However, as far as we know, no prior reports have documented the development of increased bacterial sensitivity, as observed in Gram-negative (*E. coli*) bacteria in this study. To the best of our knowledge, this research represents the first-ever evaluation of a cationic polyester dendrimer in a heterofunctional configuration against both Gram-positive and Gram-negative bacterial model strains with a particular emphasis on understanding their potential impact on the development of bacterial resistance.

### Evaluation of Hydrolytic Stability and Its Impact on the Antibacterial Activity

The hydrolytic stability of cationic polymers is an important consideration for antibacterial applications, especially at physiological pH levels and temperature.^9,24,25^ The perfect balance between the stability and degradability of the designed molecule assures sustained antimicrobial activity along with consequent clearance from the body to mitigate risks associated with bioaccumulation and potential long-term toxicity. As seen in Figure 5A, the hydrolytic stability of G2-(PA-NH_3_^+^)_9_-(β-Ala-NH_3_^+^)_12_ was determined in sterilized deionized (DI) water (pH 6.5) and PBS (pH 7.4) at 37 °C. This was accomplished by conducting MIC experiments at different time intervals, utilizing the same stock solution in *E. coli* and *S. aureus*, respectively. The MIC values were measured after 18 h starting from the respective time of treatment. Figure 5B,C represents the stability curve of G2-(PA-NH_3_^+^)_9_-(β-Ala-NH_3_^+^)_12_ at physiological conditions, based on the recorded MIC values for both bacterial strains at varying time intervals. First, it can be seen that the MIC values recorded for G2-(PA-NH_3_^+^)_9_-(β-Ala-NH_3_^+^)_12_ in DI water and PBS show a difference of one dilution factor in both bacteria at all time intervals. This variability is not considered significant as literature suggests that a single-step dilution shift in MIC is generally insignificant due to technical or experimental variability.^50^ Consequently, a 4.0-fold increase in the MIC is typically required to indicate a clinically relevant difference in bacteriostatic studies. Next, with regard to the stability trend of G2-(PA-NH_3_^+^)_9_-(β-Ala-NH_3_^+^)_12_, it was observed that in *E. coli* the MIC values of 120 μg/mL in DI water and 250 μg/mL in PBS remained constant up to 30 h and then increased by 4 times to 500 and 1000 μg/mL at 96 h, respectively. A similar trend was observed in *S. aureus*, indicating a 4.0-fold increase in MIC values for both DI water and PBS.

**Figure 5:**
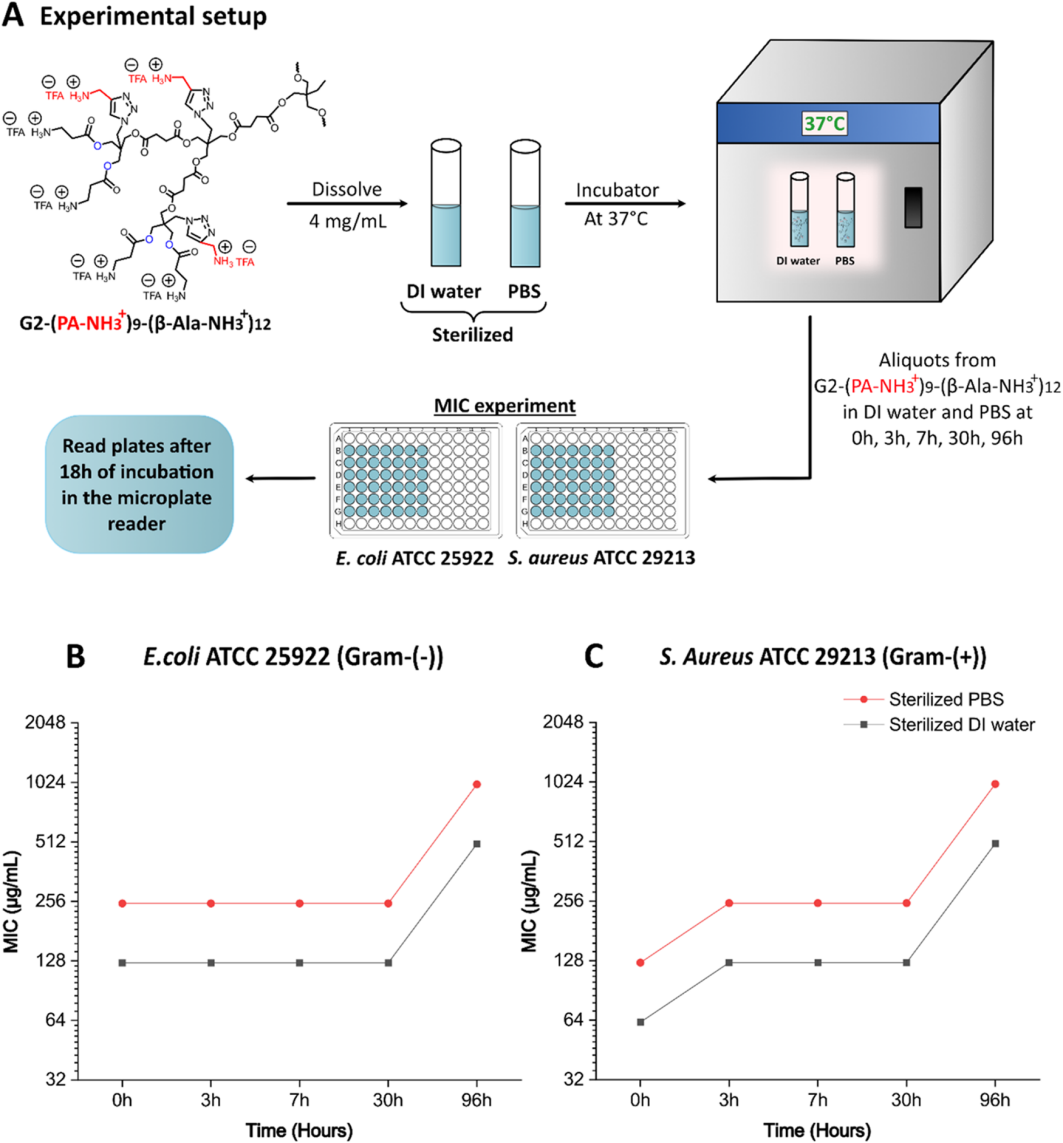
(A) An overview of the experimental setup for the determination of hydrolytic stability of G2-(PA-NH_3_^+^)_9_-(β-Ala-NH_3_^+^)_12_ at physiological conditions. Stability curve of G2-(PA-NH_3_^+^)_9_-(β-Ala-NH_3_^+^)_12_ in sterilized DI water and PBS at 37 °C, based on obtained MIC values for different time intervals in (B) gram-negative bacteria: *E. coli* and (C) Gram-positive bacteria: *S. aureus*.

These observations suggest that G2-(PA-NH_3_^+^)_9_-(β-Ala-NH_3_^+^)_12_ was hydrolytically stable in both DI water and PBS for 30 h. The drastic increase in MIC after 4 days (96 h) indicates that the second-generation cationic dendrimer began degrading in water at 37 °C, because of which it lost its initial antibacterial potency. This can also be correlated to the degradation evaluation of β-alanine-functionalized bis-MPA dendrimers by MALDI-TOF in a previous study,^25^ where G2-(β-Ala-NH_3_^+^)_12_ lost all its amine functionalities within 1 week at pH 7.4 and 37 °C due to the hydrolyzable β-alanine ester linkages connected to the periphery of the dendrimer. Since G2-(PA-NH_3_^+^)_9_-(β-Ala-NH_3_^+^)_12_ also shares structural similarity with G2-(β-Ala-NH_3_^+^)_12_ in terms of the peripheral β-alanine linkages, it is plausible that the loss in bacteriostatic activity was due to the rapid degradation of cationic β-alanine functionalities at physiological conditions. However, it is interesting to note that the earlier study indicated that at pH 7.4, only ∼50, ∼20, and ∼10% of the peripheral β-alanine functionalities remained intact after 8, 24, and 48 h, respectively. If a similar degradation profile were applicable to G2-(PA-NH_3_^+^)_9_-(β-Ala-NH_3_^+^)_12_ at 7 and 30 h, a significant reduction in antibacterial potency would be expected within this time frame. However, as the MIC values remained unchanged, it can be hypothesized that the internal positive charges played a synergistic role in maintaining bacteriostatic activity of the heterofunctional polycationic system. This highlights the importance of incorporating internal charges within the polyester scaffold to maintain its antibacterial activity despite loss of peripheral cationic groups. Additionally, the robust internal triazole linkages associated with these internal charges may have contributed toward the enhanced hydrolytic stability of G2-(PA-NH_3_^+^)_9_-(β-Ala-NH_3_^+^)_12_ until 30 h.

## Conclusions

Two families of cationic polyester dendrimers (generations 1–3) with an HFD configuration were successfully synthesized by using a BHP-diol AB_2_C monomer. A robust and versatile synthetic strategy employing CuAAC, one-pot TFA deprotection, and FPE/anhydride-coupling aided the facile synthesis of these polycationic systems. The first family presented internal cationic charges with peripheral hydroxyl functionalities, while the second family displayed both internal and external ammonium groups. The β-alanine-functionalized dendrimers from the second family exhibited significantly higher antibacterial activity compared to the internal propargyl amine-based dendrimers of the first family. Notably, both cationic families demonstrated excellent biocompatibility at concentrations relevant to their antimicrobial efficacy. Among the synthesized constructs, the second and third-generation β-alanine dendrimers exhibited the most potent antibacterial activity against Gram-positive and Gram-negative bacteria. While G3-(PA-NH_3_^+^)_21_-(β-Ala-NH_3_^+^)_24_ demonstrated superior activity against Gram-negative bacteria, its higher cytotoxicity, synthetic challenges, and lower yields favored the selection of G2-(PA-NH_3_^+^)_9_-(β-Ala-NH_3_^+^),_12_ considering its potential for future clinical translation. In a cyclic antibiotic resistance assay, G2-(PA-NH_3_^+^)_9_-(β-Ala-NH_3_^+^)_12_ developed significant resistance within just 5 days in *S. aureus*. Alternatively, the second-generation derivative induced increased sensitivity in the *E. coli* over 15 days, reducing its original MIC by half, showcasing species-dependent resistance mechanisms. Additionally, the chosen cationic dendrimer was hydrolytically stable up to 30 h under physiological conditions with consistent MIC values, while its bacteriostatic activity diminished after 4 days. This demonstrates the rapid biodegradation of the cationic polyester scaffold under physiological conditions in contrast to the prolonged stability of commercial PAMAM dendrimers, highlighting its more favorable degradation profile for biomedical applications. These findings showcase the potential of cationic polyester HFDs as promising antimicrobial agents, emphasizing the critical role of charge localization and number in modulating the antibacterial activity across different bacterial strains, thereby enhancing their likelihood for clinical translation.

## Supplementary Material



## Notes

Synthetic protocols of all heterofunctional cationic polyester dendrimers; ^1^H and ^13^C NMR spectra; DOSY spectra (Generation 3 β-alanine derivatives); MALDI-TOF spectra, SEC chromatographs; and FTIR spectra (PDF)
