# Supplementary material for: Heterofunctional Cationic Polyester Dendrimers as Antibacterial Agents: The Role of Internal and External Charges
Source: Biomacromolecules. 2025 Aug 5;26(9):6164–76. doi: 10.1021/acs.biomac.5c01094 (PMC12421690; doi:10.1021/acs.biomac.5c01094)
Supplement: Supplementary file 1 [file bm5c01094_si_001.pdf]

Electronic Supporting Information

Heterofunctional Cationic Polyester Dendrimers as Antibacterial Agents: The Role of Internal and External Charges

Arunika Singh[1], Natalia Sanz del Olmo[1,2]* and Michael Malkoch[1]*

[1]Department of Fibre and Polymer Technology, KTH Royal Institute of Technology, 100 44, Stockholm, Sweden.

[2]University of Alcala, Faculty of Sciences, Department of Organic and Inorganic Chemistry, and Research Institute in Chemistry "Andrés M. Del Río" (IQAR), 28805 Madrid, Spain.

*Author to whom correspondence should be addressed.

Corresponding authors: Prof. Michael Malkoch and Dr. Natalia Sanz del Olmo

Contact info: School of Engineering Sciences in Chemistry, Biotechnology and Health; Department of Fibre and Polymer Technology; Division of Coating Technology Teknikringen 48, SE-10044, Stockholm (malkoch@kth.se) Fax: (+) 46 (0)8 790 82 83. Department of Organic and Inorganic Chemistry, and Research Institute in Chemistry "Andrés M. del Río" (IQAR), University of Alcalá, Madrid 28805, Spain (natalia.sanzo@uah.es).

Table of Contents

General information 4

Synthesis protocols 5

Figures 22

Figure S1. ^{1}H and ^{13}C NMR spectra of G1-(PA-NHBoc)$_3$-(Ac)$_3$ in $CDCl_3$

Figure S2. ^{1}H and ^{13}C NMR spectra of G1-(PA-NHBoc)$_3$-(OH)$_6$ in CD_3OD

Figure S3. ^{1}H and ^{13}C NMR spectra of G1-(PA-NH_3^+)$_3$-(OH)$_6$ in CD_3OD

Figure S4. ^{1}H and ^{13}C NMR spectra of G2-(PA-NHBoc)$_9$-(OH)$_{12}$ in CD_3OD

Figure S5. ^{1}H and ^{13}C NMR spectra of G2-(PA-NH_3^+)$_9$-(OH)$_{12}$ in CD_3OD

Figure S6. ^{1}H and ^{13}C NMR spectra of G3-(PA-NHBoc)$_{21}$-(Ac)$_{12}$ in $CDCl_3$

Figure S7. ^{1}H and ^{13}C NMR spectra of G3-(PA-NHBoc)$_{21}$-(OH)$_{24}$ in CD_3OD

Figure S8. ^{1}H and ^{13}C NMR spectra of G3-(PA-NH_3^+)$_{21}$-(OH)$_{24}$ in CD_3OD

Figure S9. ^{1}H and ^{13}C NMR spectra of G1-(PA-NHBoc)$_3$-(β-Ala-NHBoc)$_6$ in $CDCl_3$

Figure S10. ^{1}H and ^{13}C NMR spectra of G1-(PA-NH_3^+)$_3$-(β-Ala-NH_3^+)$_6$ in CD_3OD

Figure S11. ^{1}H and ^{13}C NMR spectra of G2-(PA-NHBoc)$_9$-(β-Ala-NHBoc)$_{12}$ in $CDCl_3$

Figure S12. ^{1}H and ^{13}C NMR spectra of G2-(PA-NH_3^+)$_9$-(β-Ala-NH_3^+)$_{12}$ in CD_3OD

Figure S13. ^{1}H and ^{13}C NMR spectra of G3-(PA-NHBoc)$_{21}$-(β-Ala-NHBoc)$_{24}$ in $CDCl_3$

Figure S14. DOSY spectra of G3-(PA-NHBoc)$_{21}$-(β-Ala-NHBoc)$_{24}$ in $CDCl_3$

Figure S15. ^{1}H and ^{13}C NMR spectra of G3-(PA-NH_3^+)$_{21}$-(β-Ala-NH_3^+)$_{24}$ in CD_3OD

Figure S16. DOSY spectra of G3-(PA-NH_3^+)$_{21}$-(β-Ala-NH_3^+)$_{24}$ in CD_3OD

Figure S17. Stacked FTIR spectra of G1-(N_3)$_3$-(Ac)$_3$, G1-(PA-NHBoc)$_3$-(Ac)$_3$ and free PA-NHBoc

Figure S18. Stacked FTIR spectra of G3-(N_3)$_{21}$-(Ac)$_{12}$, G3-(PA-NHBoc)$_{21}$-(Ac)$_{12}$ and free PA-NHBoc

Figure S19. SEC overlay of G1-(PA-NHBoc)$_3$-(Ac)$_3$ and G3-(PA-NHBoc)$_{21}$-(Ac)$_{12}$

Figure S20. Stacked MALDI-TOF spectra of G1-(PA-NHBoc)$_3$-(Ac)$_3$ and G3-(PA-NHBoc)$_{21}$-(Ac)$_{12}$ in DCTB

Figure S21. SEC overlay of G1-(PA-NHBoc)$_3$-(OH)$_6$, G2-(PA-NHBoc)$_9$-(OH)$_{12}$ and G3-(PA-NHBoc)$_{21}$-(OH)$_{24}$

Figure S22. Stacked MALDI-TOF spectra of G1-(PA-NHBoc)$_3$-(OH)$_6$, G2-(PA-NHBoc)$_9$-(OH)$_{12}$ and G3-(PA-NHBoc)$_{21}$-(OH)

Figure S23. Stacked MALDI-TOF spectra of G1-(PA-NH_3^+)$_3$-(OH)$_6$, G2-(PA-NH_3^+)$_9$-(OH)$_{12}$ and G3-(PA-NH_3^+)$_{21}$-(OH)$_{24}$ in DHB

Figure S24. SEC overlay of G1-(PA-NHBoc)$_3$-(β-Ala-NHBoc)$_6$ and G2-(PA-NHBoc)$_9$-(β-Ala-NHBoc)$_{12}$

Figure S25. Stacked MALDI-TOF spectra of G1-(PA-NHBoc)$_3$-(β-Ala-NHBoc)$_6$ and G2-(PA-NHBoc)$_9$-(β-Ala-NHBoc)$_{12}$ in DCTB

Figure S26. Stacked MALDI-TOF spectra of G1-$(PA)_3$-(β-Ala-$NH3^+$)$_6$ in DHB and G2-$(PA)_9$-(β-Ala-$NH3^+$)$_{12}$ in DCTB

References..**35**

General Information

Abbreviations

Ac	Acetonide
Boc	Di-tert-butyl dicarbonate
PA-NHBoc	Boc protected propargyl amine
PA-NH_3^+	Propargylammonium trifluoroacetate
β-Ala-NHBoc	Boc protected β-alanine
β-Ala-NH_3^+	β-alaninium trifluoroacetate
br	Broad signal
$CuSO_4.5H_2O$	Copper sulfate
CsF	Cesium Fluoride
DCC	N,N'-Dicyclohexylcarbodiimide
DCM	Dichloromethane
DCU	1,3-Dicyclohexyl urea
DTCB	Trans-2-[3-(4-tert-Butylphenyl)-2-methyl-2-propenylidene]malononitrile
DHB	2,5-dihydroxybenzoic acid
DMAP	2,2-Dimethoxy propane
DMF	Dimethylformamide
EtOAc	Ethyl acetate
FPE	Fluoride-promoted esterification
MALDI-TOF	Matrix-assisted laser desorption ionization time-of-flight
Na ascorbate	Sodium ascorbate
NMR	Nuclear magnetic resonance
SEC	Size Exclusion Chromatography
TFA	Trifluoroacetic acid
$NaHCO_3$	Sodium bicarbonate
$NaHSO_4$	Sodium bisulfate
THF	Tetrahydrofuran

Synthesis protocols

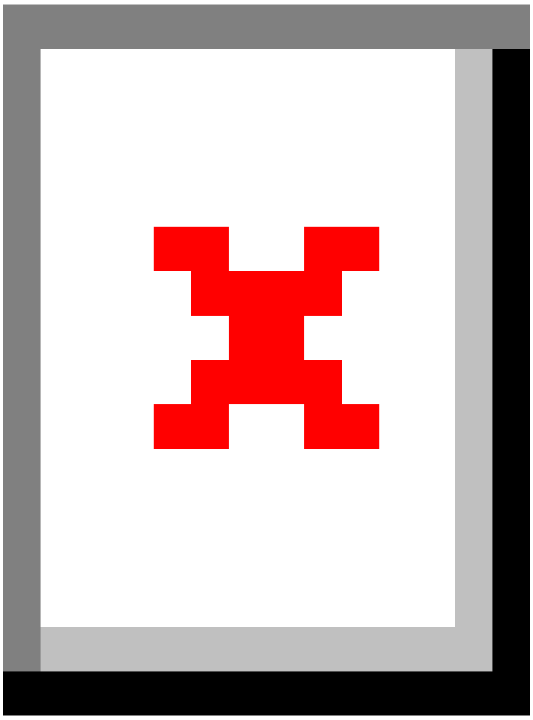

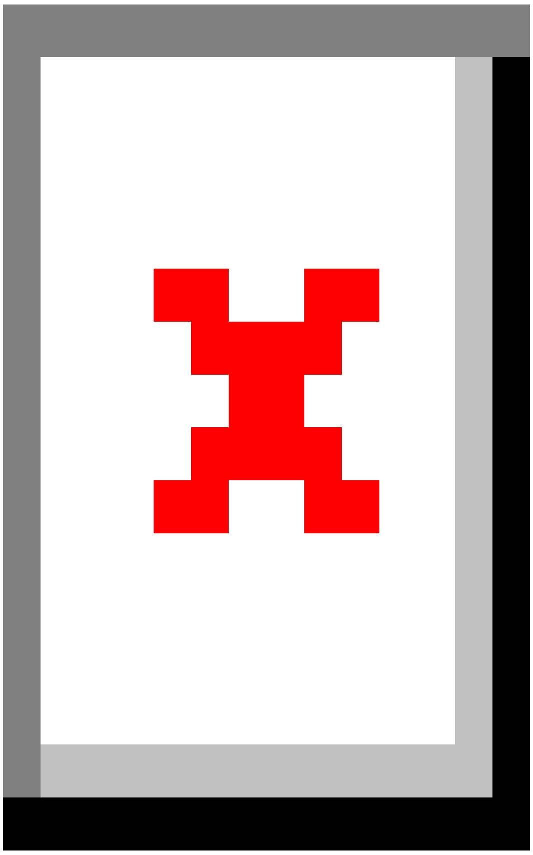

The compounds listed were synthesized according to previous publications.[1,2,3] A) G1-$(N_3)_3$-$(Ac)_3$[1] B) G2-$(N_3)_9$-$(Ac)_6$[1] C) G3-$(N_3)_{21}$-$(Ac)_{12}$[1] D) Boc protected propargyl amine[1] E) G2-$(PA\text{-}NHBoc)_9$-$(Ac)_6$[1] F) Imidazolide-activated β-Ala-NHBoc[2,3] G) β-Ala-NHBoc anhydride[2] H) G2-$(\beta\text{-}Ala\text{-}NH_3^+)_{12}$[2,3]

General procedure for PA-NHBoc functionalization

The general procedure for post-functionalization of azide-based dendrimers with PA-NHBoc[1] follows the same CuAAC protocol as previously published[1] with modifications in the purification steps. Briefly, the azide-based acetonide protected dendrimer (1 eq) and PA-NHBoc (1.5 eq/N_3 group) are reacted overnight at rt in a 1:1 THF:H_2O mixture, in the presence of $CuSO_4.5H_2O$ and Na ascorbate. After completion of reaction, the crude product is first washed with 0.5% w/w EDTA (3x) solution to remove $CuSO_4.5H_2O$ and Na ascorbate, then purified through a silica plug to eliminate excess of PA-NHBoc using an appropriate eluent depending on the specific PA-NHBoc functionalized dendrimer.

General acetonide deprotection procedure for the synthesis of dendrimers with hydroxyl-functional groups

The PA-NHBoc functionalized acetonide-protected dendrimer is dissolved in MeOH with consequent addition of the acidic resin Dowex™ 50WX2 50-100 (H). The deprotection reaction is carried out at 45°C with stirring for a few hours. The ongoing reaction is monitored for completion by NMR and MALDI-TOF analysis. After completion of the reaction, the Dowex resin is filtered out, and the MeOH is evaporated to yield the deprotected derivative.

General one-pot deprotection procedure for the synthesis of dendrimers with internal ammonium groups and external hydroxyl functionalities

The one-pot deprotection of PA-NHBoc functionalized acetonide protected dendrimers was conducted in TFA (12 eq/PA-NHBoc and acetonide group) with DCM (same volume as TFA) as a co-solvent. The reaction is carried out at room temperature for few hours, followed by evaporation of TFA and DCM overnight upon reaction completion. The crude product obtained is purified the following day by precipitations in ether (3x) after dissolution in the minimal volume of MeOH, with consequent removal of solvent.

General procedure for FPE

The esterification of peripheral hydroxyl groups with β-Ala-NHBoc in the G1 and G2 dendrimers follows the FPE protocol as previously published[2] with slight modifications in the purification steps depending on the specific dendritic generation. Briefly, CDI (1 eq) is slowly added to a solution of β-Ala-NHBoc (1 eq) in DCM. After one hour of stirring at rt, the CDI activated β-Ala-NHBoc[2] (1.5 eq/OH group) is added to the solution of hydroxy-functional dendrimer (1 eq) in DCM with consequent addition of CsF (0.2 eq/OH group). The reaction mixture is stirred at rt overnight. The reaction progress is monitored via NMR and MALDI-TOF spectroscopy. For purification of G1 dendrimer, the excess of CDI-activated β-Ala-NHBoc is quenched with water and vigorous stirring upon reaction completion. The crude reaction mixture diluted in DCM is washed with aqueous solutions of 10% $NaHCO_3$, 10% $NaHSO_4$, and brine before drying with $MgSO_4$ and evaporation of solvent. For purification of G2 dendrimer, instead of the quenching and washing steps, the concentrated crude product in DCM is precipitated in ether with consequent removal of solvent.

General procedure for anhydride-based esterification

The esterification of peripheral hydroxyl groups with β-Ala-NHBoc in the G3 dendrimer follows the anhydride-based esterification protocol as previously published.[2] Briefly, β-Ala-NHBoc (2 eq) is dissolved in a flask containing DCM which is placed on an ice bath. Consequently, a solution of DCC (1 eq) in DCM is poured over the cold reaction mixture and the reaction is left to stir overnight. The DCU formed in the reaction is filtered the following day through celite to obtain the β-Ala-NHBoc anhydride[2]. The third-generation hydroxyl functional dendrimer (1 eq) is dissolved in a flask containing

DCM to which the bases DMAP (0.2 eq/OH group) and pyridine (5 eq/OH group) are added. Next, a solution of the β-Ala-NHBoc anhydride[2] (1.6 eq/OH group) dissolved in DCM is added to the reaction mixture containing the precursor with bases and the reaction is left to run at rt overnight. The reaction completion is monitored through NMR spectroscopy. The concentrated crude product in DCM is precipitated in ether (5x) with consequent removal of solvent.

G1-(PA-NHBoc)$_3$-(Ac)$_3$

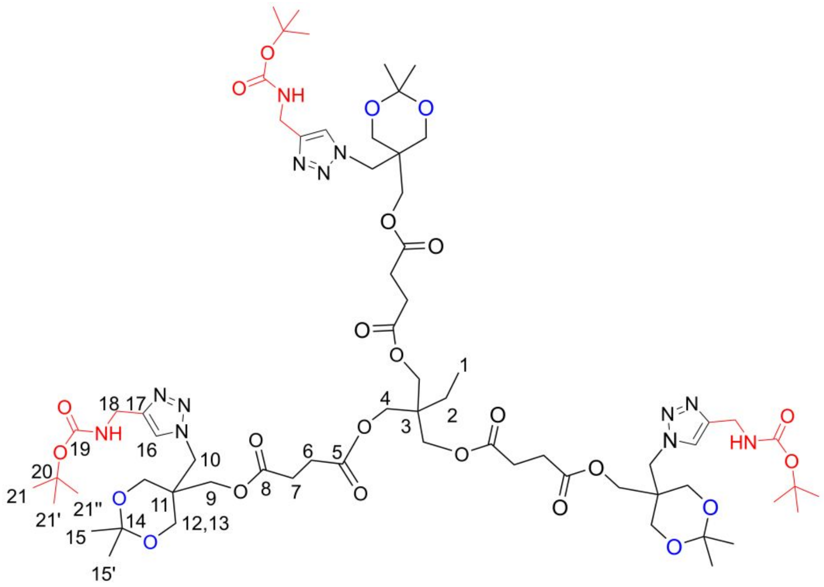

G1-$(N_3)_3$-$(Ac)_3$[1] (0.41 g, 0.41 mmol) was post-functionalized with PA-NHBoc[1] (0.29 g, 1.86 mmol) by running the reaction in 3 mL of a 1:1 mixture of THF and H_2O, utilizing the general CuAAC protocol. The reaction was facilitated by the addition of CuSO4·5H_2O (0.062 g, 0.25 mmol) and sodium ascorbate (0.098 g, 0.49 mmol). After washing with 0.5% w/w EDTA, G1-(PA-NHBoc)$_3$-(Ac)$_3$ was first eluted in a mixture of 50:50 EtOAc:heptane and later in EtOAc to obtain the pure product as a colorless oil after concentration. (0.45 g, 76%). $C_{66}H_{104}N_{12}O_{24}$ (1449.62 g mol^{-1}). ^{1}H-NMR (400 MHz, CDCl$_3$) δ/ppm: 7.61 (3H, s, H16), 4.53 (6H, s, H10), 4.35 (6H, s, H18), 3.98 (12H, m, H9, H4), 3.63 (12H, m, H12, H13), 2.62 (12H, m, H6, H7), 1.41 (47H, m, H2, H15, H15', H21, H21'and H21''), 0.86 (3H, m, H1). ^{13}C-NMR (101 MHz, CDCl$_3$) δ/ppm: 172.09 (C5), 171.85 (C8), 155.92 (C19), 124.03 (C16, C17), 98.98 (C14), 79.69 (C20), 64.25 (C4), 63.65 (C9), 62.91 (C12, C13), 49.92 (C10), 40.88 (C3), 38.28 (C11), 36.14 (C18), 28.92 (C6, C7), 28.89 (C6, C7), 28.46 (C21, C21' and C21''), 25.79 (C15), 23.07 (C2), 21.75 (C15'), 7.44 (C1). MALDI: Calc. [M+Cu$^+$] = 1513.16 Da, Found [M+Cu$^+$] = 1521.88 Da. SEC (DMF) M_n = 1968.0 g mol^{-1}, M_w= 2028.1 g mol^{-1}, Đ = 1.03.

G1-(PA-NHBoc)$_3$-(OH)$_6$

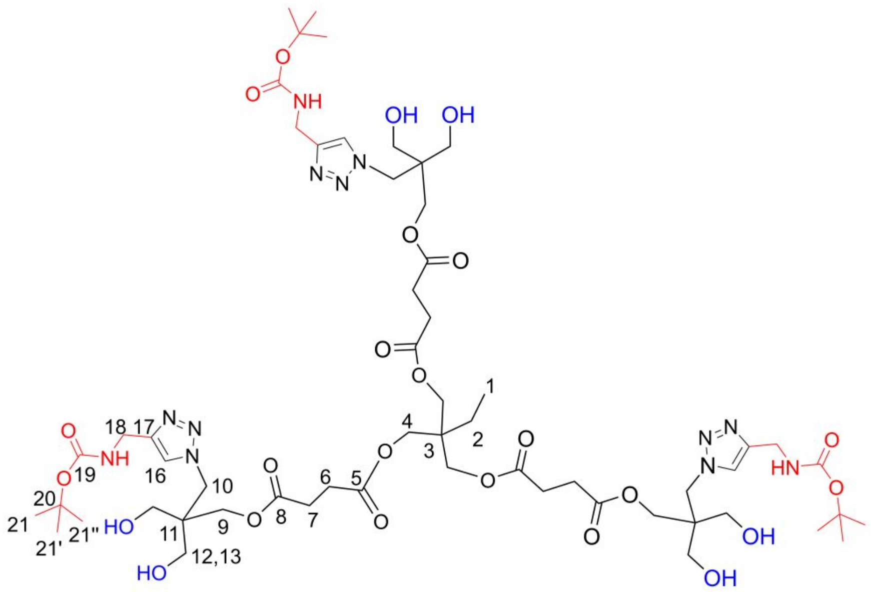

G1-(PA-NHBoc)$_3$-(OH)$_6$ was synthesized following the general acetonide deprotection procedure, utilizing the specified reagents with their indicated quantities: G1-(PA-NHBoc)$_3$-(Ac)$_3$ (70.0 mg, 0.048 mmol) and DOWEX (70.0 mg). After concentration, G1-(PA-NHBoc)$_3$-(OH)$_6$ was obtained as a viscous oil. (32.0 mg, 50%). $C_{57}H_{92}N_{12}O_{24}$ (1329.42 g mol^{-1}).^{1}H-NMR (400 MHz, MeOD) δ/ppm: 7.87 (3H, s, H16), 4.49 (6H, s, H10), 4.31 (6H, s, H18), 4.03 (12H, m, H9, H4), 3.48 (12H, m, H12, H13), 2.63 (12H, m, H6, H7), 1.44 (29H, m, H2, H21, H21', H21''), 0.89 (3H, m, H1). ^{13}C-NMR (101 MHz, MeOD) δ/ppm: 173.81 (C5), 173.76 (C8), 158.23 (C19), 146.86 (C17), 125.98 (C16), 80.45 (C20), 65.14 (C4), 64.61 (C9), 61.57 (C12, C13), 50.76 (C10), 46.40 (C11), 42.16 (C3), 36.68 (C18), 29.88 (C6, C7), 29.83 (C6, C7), 28.75 (C21, C21', C21''), 23.97 (C2), 7.72 (C1). MALDI: Calc. [M+Na$^+$] = 1352.40 Da, Found [M+Na$^+$] = 1351.61 Da. SEC (DMF) M_n = 2213.9 g mol^{-1}, M_w= 2267.6 g mol^{-1}, Đ = 1.02.

G1-(PA-NH_3^+)$_3$-(OH)$_6$

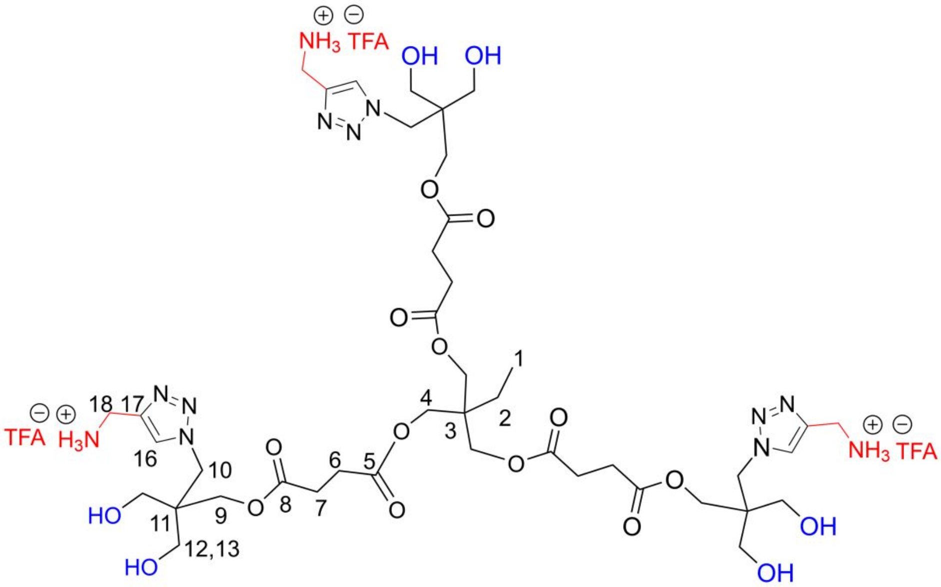

G1-($NH_3^+TFA^-$)$_3$-(OH)$_6$ was synthesized following the general one-pot deprotection procedure with a reaction time of 2 h, utilizing the specified reagents with their indicated quantities: G1-(PA-NHBoc)$_3$-(Ac)$_3$ (150 mg, 0.1 mmol), TFA (570.0 µL, 7.45 mmol) and DCM (570.0 µL). After precipitations in ether, G1-($NH_3^+TFA^-$)$_3$-(OH)$_6$ was freeze dried to obtain a white powder. (120 mg, 87%). $C_{48}H_{71}N_{12}O_{24}F_9$ (1371.15 g mol^{-1}).^{1}H-NMR (400 MHz, MeOD) δ/ppm: 8.09 (3H, s, H16), 4.54 (6H, s, H10), 4.27 (6H, s, H18), 4.04 (12H, m, H4, H9), 3.48 (12H, m, H12, H13), 2.66 (12H, m, H6, H7), 1.51 (2H, m, H2), 0.91 (3H, m, H1). ^{13}C-NMR (101 MHz, MeOD) δ/ppm: 173.90 (C5), 173.87 (C8), 163.22 (C=O, TFA), 162.88 (C=O, TFA), 140.99 (C17), 127.38 (C16), 119.70 (CF_3, TFA), 116.79 (CF_3, TFA), 65.22 (C4), 64.47 (C9), 61.56 (C12, C13), 50.82 (C10), 46.45 (C11), 42.19 (C3), 35.50 (C18), 29.87 (C6, C7), 29.83 (C6, C7), 24.03 (C2), 7.69 (C1). MALDI: Calc. [M+H$^+$] = 1033.09 Da, Found [M+H$^+$] = 1033.07 Da. Calc. [M+Na$^+$] = 1055.08 Da, Found [M+Na$^+$] = 1051.48 Da.

G2-(PA-NHBoc)$_9$-(OH)$_{12}$

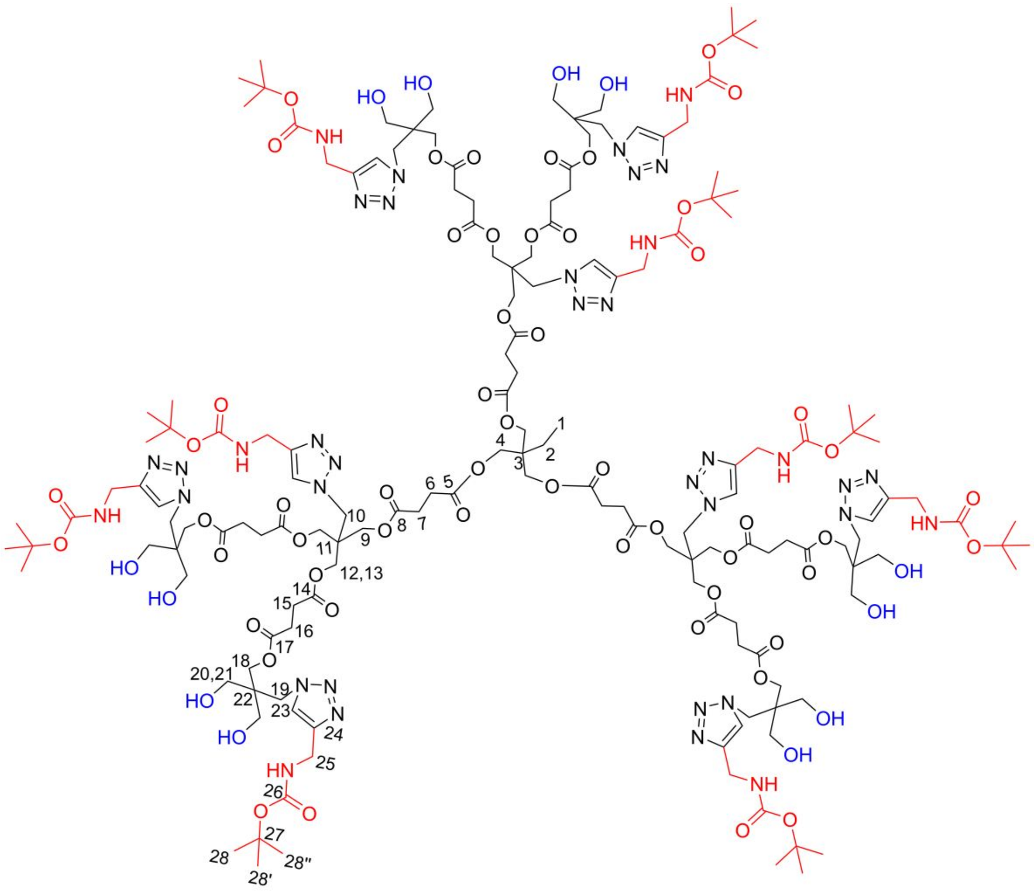

G2-(PA-NHBoc)$_9$-(OH)$_{12}$ was synthesized following the general acetonide deprotection procedure, utilizing the specified reagents with their indicated quantities: G2-(PA-NHBoc)$_9$-(Ac)$_6$[1] (100.0 mg, 0.025 mmol) and DOWEX (100.0 mg). After concentration, G2-(PA-NHBoc)$_9$-(OH)$_{12}$ was obtained as a viscous oil. (70.0 mg, 75%). $C_{159}H_{248}N_{36}O_{66}$ (3719.92 g mol^{-1}). ^{1}H-NMR (400 MHz, MeOD) δ/ppm: 7.88 (9H, m, H23), 4.54 (18H, m, H10, H19), 4.30 (18H, m, H25), 4.08 (36H, m, H9, H4, H18, H12 and H13), 3.53 – 3.48 (24H, m, H20, H21), 2.65 (36H, m, H6, H7, H15 and H16), 1.43 (83H, m, H2, H28, H28', H28''), 0.89 (3H, m, H1). ^{13}C-NMR (101 MHz, MeOD) δ/ppm: 173.83 (C5), 173.76 (C8), 173.48 (C14), 173.43 (C17), 158.18 (C26), 146.90 (C24), 125.93 (C23), 80.45 (C27), 65.36 (C4), 64.72 (C9, C18), 64.08 (C12, C13), 61.63 (C20, C21), 51.11 (C10, C19), 50.74 (C10, C19), 46.40 (C22), 44.09 (C11), 36.74 (C3), 29.89 (C6, C7, C15, C16), 29.66 (C6, C7, C15, C16), 28.84 (C28, C28' and C28''), 28.80 (C28, C28' and C28''), 24.15 (C2), 7.90 (C1). MALDI: Calc. [M+K$^+$] = 3759.02 Da, Found [M+K$^+$] = 3767.82 Da. SEC (DMF) M_n = 5336.8 g mol^{-1}, M_w= 5531.6 g mol^{-1}, Đ = 1.03.

G2-(PA-NH_3^+)$_9$-(OH)$_{12}$

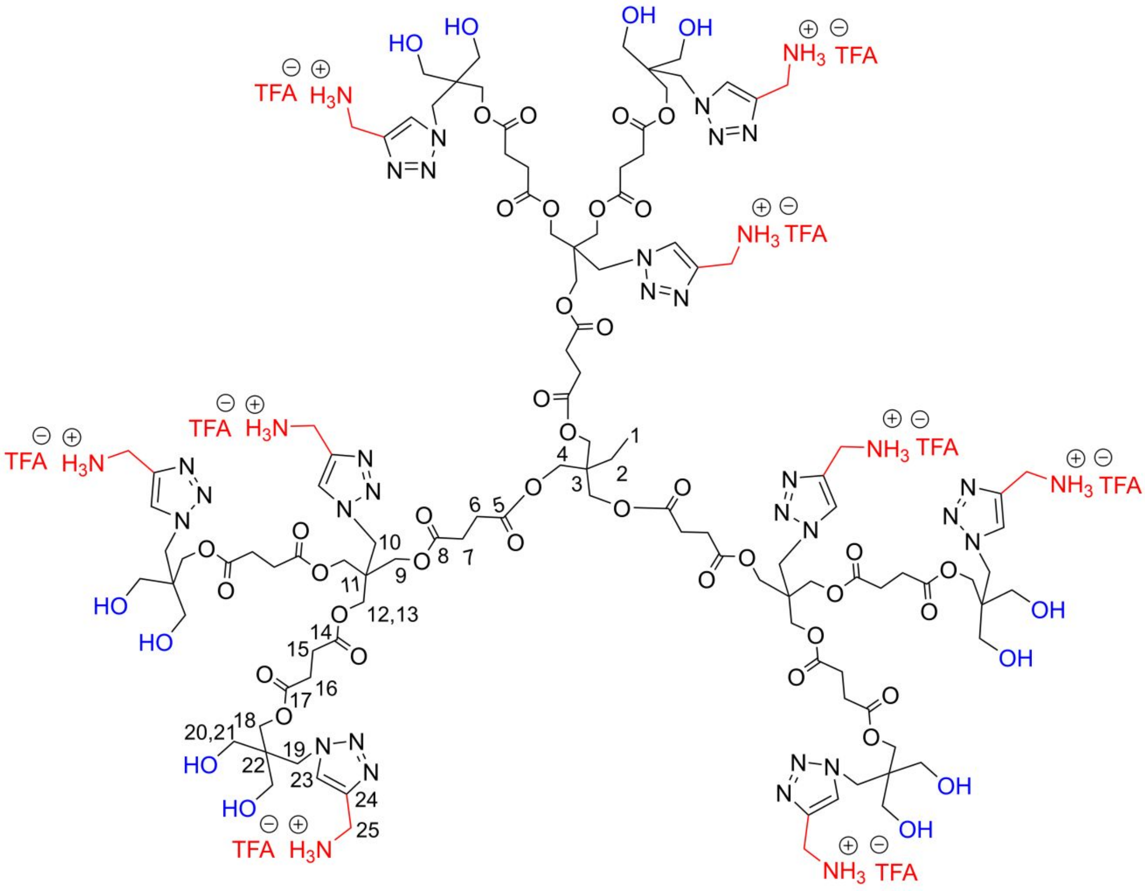

G2-($NH_3^+TFA^-$)$_9$-(OH)$_{12}$ was synthesized following the general one-pot deprotection procedure with a reaction time of 4 h, utilizing the specified reagents with their indicated quantities: G2-(PA-NHBoc)$_9$-(Ac)$_6$ (150 mg, 0.037 mmol), TFA (521.0 µL, 6.81 mmol) and DCM (521.0 µL). After precipitations in ether, G2-($NH_3^+TFA^-$)$_9$-(OH)$_{12}$ was freeze dried to obtain a white powder. (120 mg, 84%). $C_{132}H_{185}N_{36}O_{66}F_{27}$ (3845.11 g mol^{-1}). ^{1}H-NMR (400 MHz, MeOD) δ/ppm: 8.12 (9H, m, H23), 4.60 (18H, m, H10, H19), 4.30 (18H, m, H25), 4.08 (36H, m, H4, H9. H18, H12 and H13), 3.48 (24H, m, H20, H21), 2.68 (36H, m, H6, H7, H15 and H16), 1.51 (2H, m, H2), 0.90 (3H, m, H1). ^{13}C-NMR (101 MHz, MeOD) δ/ppm: 174.09 (C5), 173.90 (C8), 173.66 (C14), 173.57 (C17), 159.61 (C=O, TFA), 159.20 (C=O, TFA), 158.78 (C=O, TFA), 158.37 (C=O, TFA), 127.71 (C23), 120.24 (CF_3, TFA), 117.41 (CF_3, TFA), 114.58 (CF_3, TFA), 111.76 (CF_3, TFA), 65.38 (C4), 64.53 (C9, C18), 63.94 (C12, C13), 61.47 (C20, C21), 51.01 (C10), 50.80 (C19), 46.42 (C22), 44.13 (C11), 42.12 (C3), 35.51 (C25), 29.82 (C6, C7, C15 and C16), 24.09 (C2), 7.79 (C1). MALDI: Calc. [M+Na$^+$] = 2850.91 Da, Found [M+Na$^+$] = 2845.0 Da.

G3-(PA-NHBoc)$_{21}$-(Ac)$_{12}$

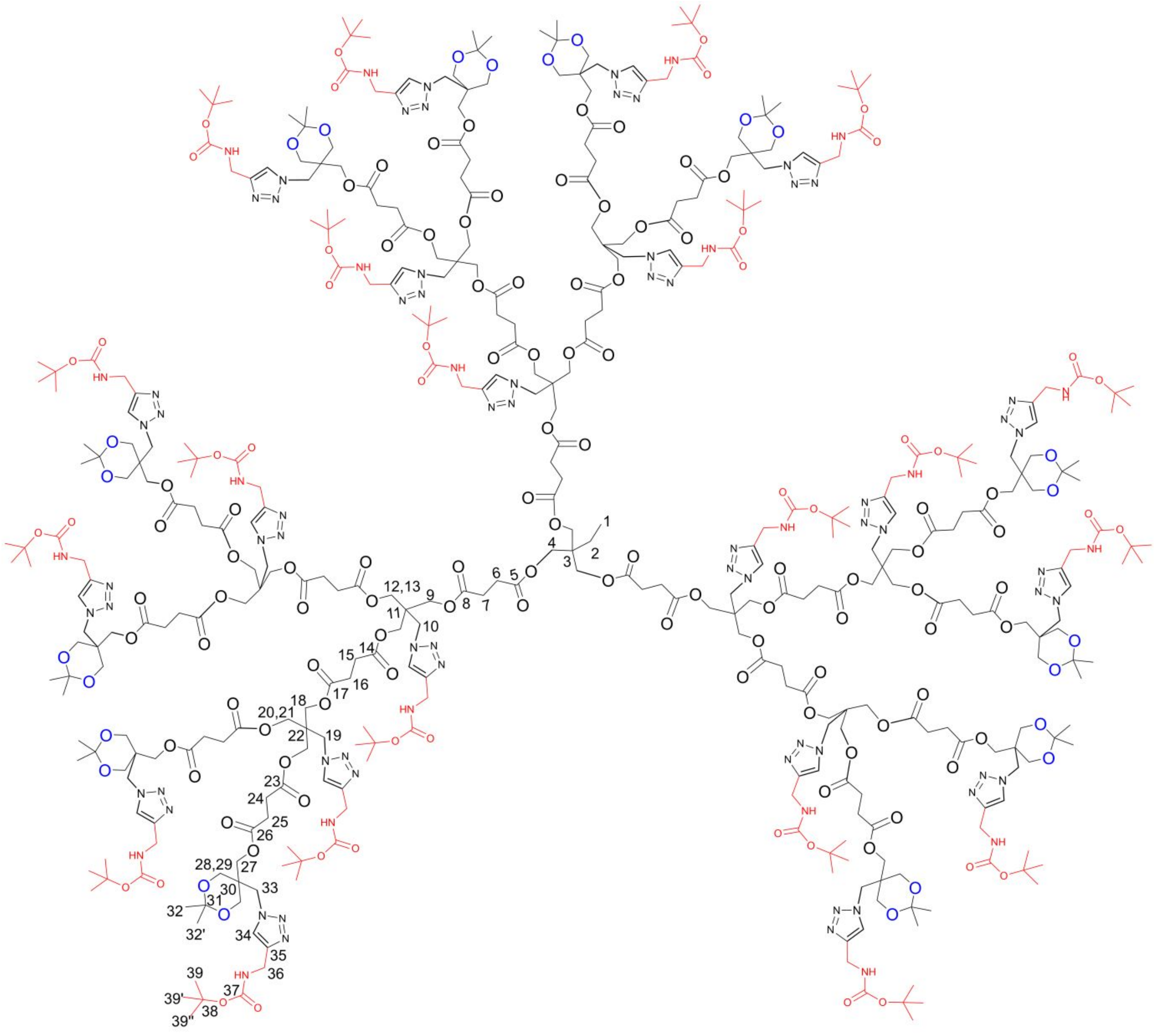

G3-(N$_3$)$_{21}$-(Ac)$_{12}$[1] (0.29 g, 0.05 mmol) was post-functionalized with PA-NHBoc[1] (0.25 g, 1.57 mmol) by running the reaction in 3 mL of a 1:1 mixture of THF and H_2O, utilizing the general CuAAC protocol. The reaction was facilitated by the addition of CuSO4·5H_2O (0.052 g, 0.21 mmol) and sodium ascorbate (0.083 g, 0.42 mmol). After washing with 0.5% w/w EDTA, G3-(PA-NHBoc)$_{21}$-(Ac)$_{12}$ was first eluted in a mixture of 50:50 EtOAc:heptane and later in MeOH to obtain the pure product as a colorless oil after concentration. (217 mg, 48%). $C_{399}H_{608}N_{84}O_{150}$ (8981.69 g mol^{-1}). ^{1}H-NMR (400 MHz, $CDCl_3$) δ/ppm: 7.69 (21H, m, H34), 4.43 (84H, m, H10, H19, H33 and H36), 4.03 (84H, m, H9, H12, H4, H13, H18, H20, H21 and H27), 3.65 (48H, m, H28, H29), 2.63 (84H, m, H6, H7, H15, H16, H24 and H25), 1.39 (263H, m, H2, H32, H32', H32'', H39, H39'and H39''), 0.85 (3H, m, H1). ^{13}C-NMR (101 MHz, $CDCl_3$) δ/ppm: 172.03 (C5, C8 and C14), 171.82 (C17, C23 and C26), 156.03 (C37), 145.43 (C35), 124.21 (C34), 99.01 (C31), 79.67 (C38), 63.86 (C9, C18 and C27), 62.90 (C28, C29, C12, C13, C20 and C21), 49.91 (C10, C19 and C33), 42.95 (C11, C22), 38.23 (C30), 36.08 (C36), 28.90 (C6, C7, C15, C16, C24 and C25), 28.51 (C39, C39' and C39''), 28.49 (C39, C39' and C39''), 25.90 (C32, C32'), 21.71 (C32, C32'), 7.51 (C1). MALDI: Calc. [M+Cu$^+$] = 9045.23 Da, Found [M+Cu$^+$] = 9068.02 Da. SEC (DMF) M_n = 9761.8 g mol^{-1}, M_w= 10481.0 g mol^{-1}, Đ = 1.07.

G3-(PA-NHBoc)$_{21}$-(OH)$_{24}$

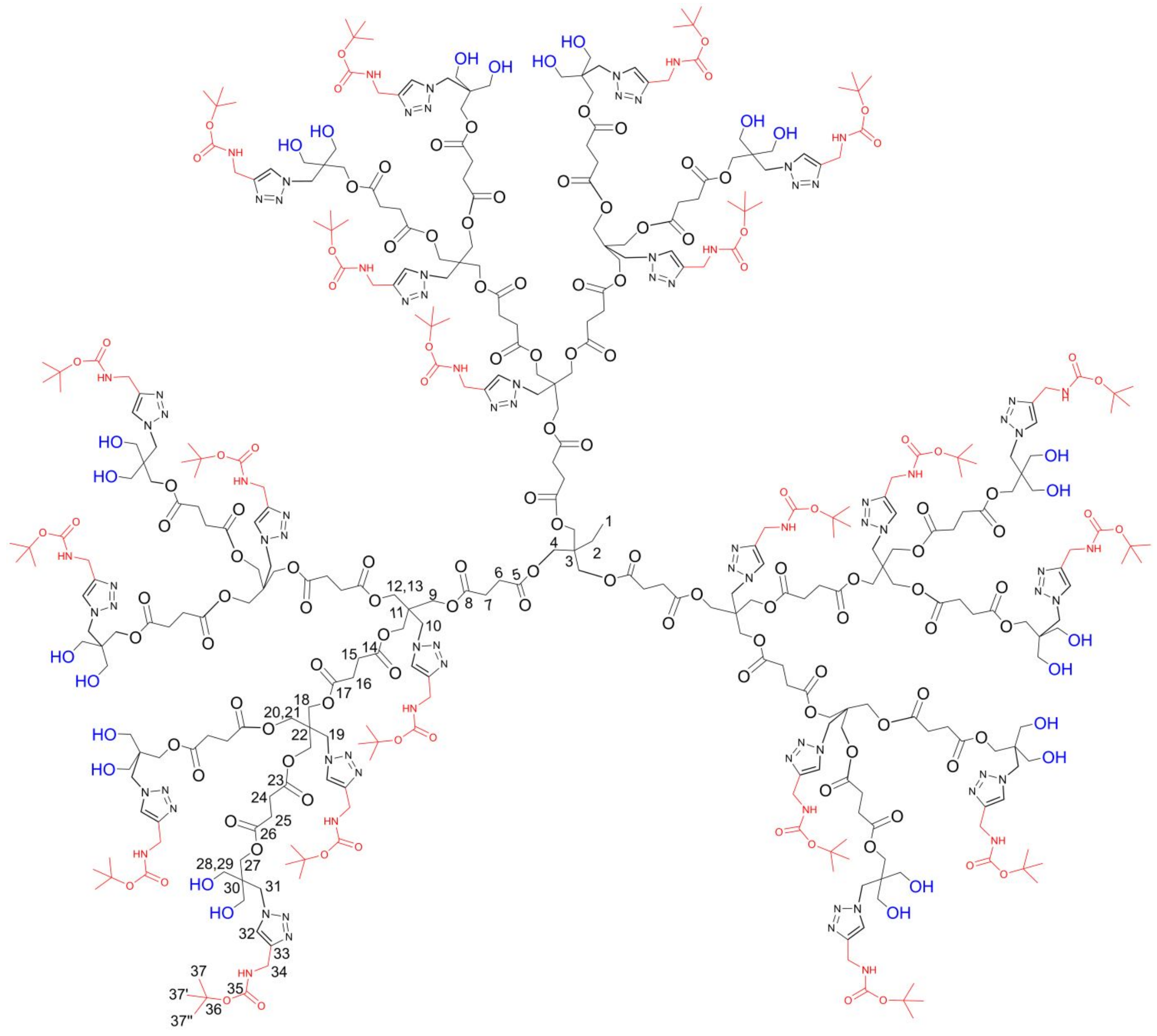

G3-(PA-NHBoc)$_{21}$-(OH)$_{24}$ was synthesized following the general acetonide deprotection procedure, utilizing the specified reagents with their indicated quantities: G3-(PA-NHBoc)$_{21}$-(Ac)$_{12}$ (100.0 mg, 0.011 mmol) and DOWEX (100.0 mg). After concentration, G2-(PA-NHBoc)$_{9}$-(OH)$_{12}$ was obtained as a viscous oil. (55.0 mg, 59%). $C_{363}H_{560}N_{84}O_{150}$ (8500.91 g mol^{-1}). ^{1}H-NMR (400 MHz, MeOD) δ/ppm: 7.88 (21H, m, H32), 4.56 (42H, m, H10, H19, and H31), 4.31 (42H, m, H34), 4.09 (84H, m, H9, H12, H4, H13, H18, H20, H21 and H27), 3.47 (48H, m, H28, H29), 2.63 (84H, m, H6, H7, H15, H16, H24 and H25), 1.42 (191H, m, H2, H37, H37' and H37''), 0.88 (3H, m, H1). ^{13}C-NMR (101 MHz, MeOD) δ/ppm: 173.92 (C5, C8 and C14), 173.58 (C17, C23 and C26), 158.18 (C35), 147.03 (C33), 125.94 (C32), 80.55 (C36), 64.73 (C9, C18), 64.12 (C27), 61.59 (C28, C29, C12, C13, C20 and C21), 51.12 (C10, C19), 50.71 (C31), 46.37 (C30), 45.26 (C11), 44.05 (C22), 42.12 (C3), 36.71 (C34), 29.87 (C6, C7, C15, C16, C24 and C25), 28.82 (C37, C37', C37''). MALDI: Calc. [M+Na^{+}] = 8523.89 Da, Found [M+Na^{+}] = 8522.67 Da. SEC (DMF) M_n = 8917.2 g mol^{-1}, M_w= 9664.5 g mol^{-1}, Đ = 1.08.

G3-(PA-NH_3^+)$_{21}$-(OH)$_{24}$

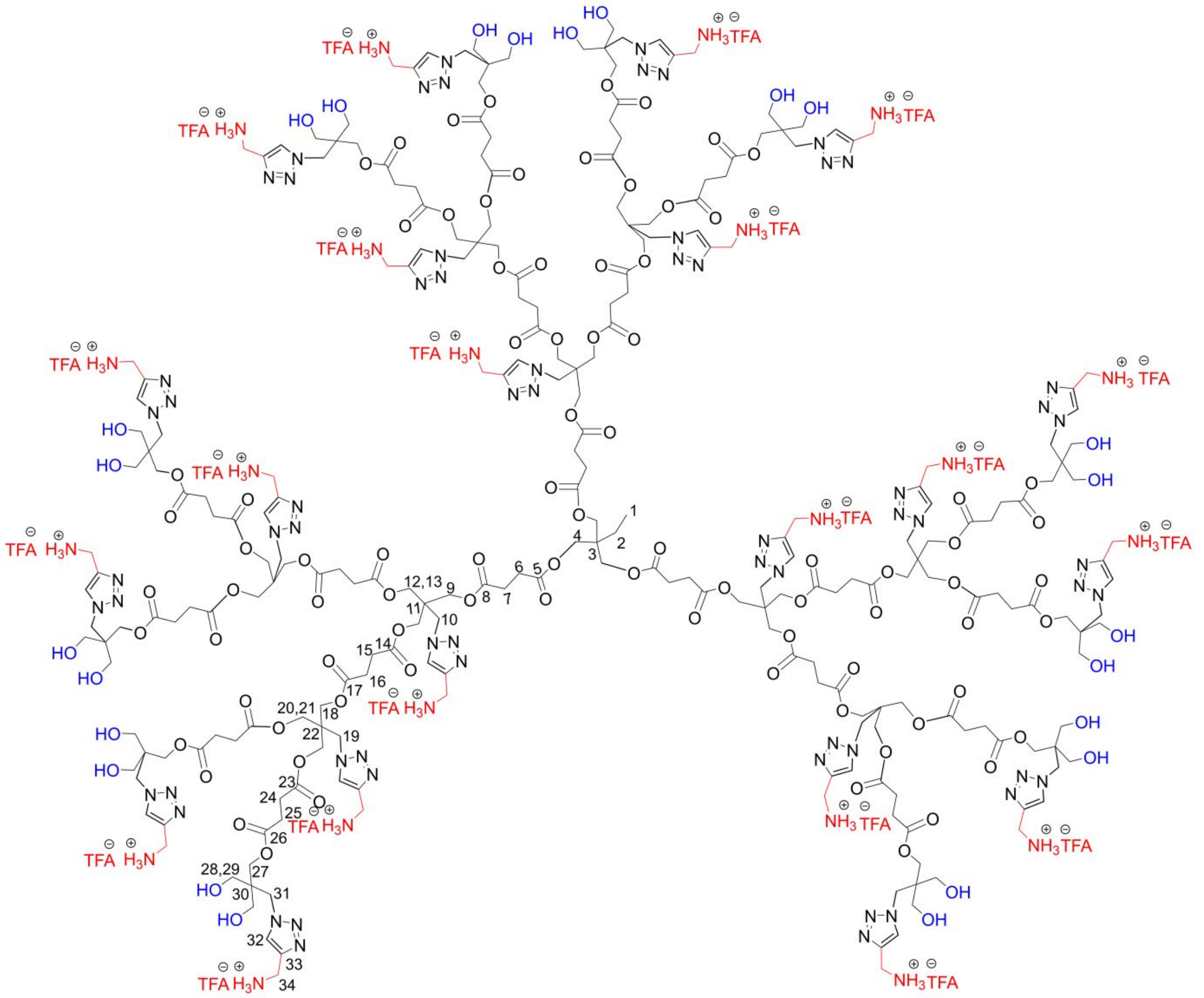

G3-(PA-NH_3^+)$_{21}$-(OH)$_{24}$ was synthesized following the general one-pot deprotection procedure with a reaction time of 8 h, utilizing the specified reagents with their indicated quantities: G3-(PA-NHBoc)$_{21}$-(Ac)$_{12}$ (60.0 mg, 0.007 mmol), TFA (43.0 µL, 0.56 mmol) and DCM (43.0 µL). After precipitations in ether, G3-(PA-NH_3^+)$_{21}$-(OH)$_{24}$ was freeze dried to obtain a white powder. (34.0 mg, 55%). $C_{300}H_{413}N_{84}O_{150}F_{63}$ (8793.03 g/mol). ^{1}H-NMR (400 MHz, MeOD) δ/ppm: 8.14 (21H, m, H32), 4.60 (42H, m, H10, H19 and H31), 4.31 (42H, m, H34), 4.09 (84H, m, H9, H12, H4, H13, H18, H20, H21 and H27), 3.48 (48H, m, H28, H29), 2.68 (84H, m, H6, H7, H15, H16, H24 and H25), 1.50 (2H, m, H2), 0.89 (3H, m, H1). ^{13}C-NMR (101 MHz, MeOD) δ/ppm: 174.09 (C5, C8 and C14), 173.63 (C17, C23 and C26), 163.07 (C=O, TFA), 141.33 (C33), 141.03 (C33), 127.62 (C32), 65.38 (C4), 64.58 (C9, C18, C27), 64.02 (C9, C18, C27), 61.50 (C28, C29, C12, C13, C20 and C21), 50.82 (C10, C19 and C31), 46.43 (C30), 44.14 (C11, C22), 42.14 (C3), 35.52 (C34), 29.83 (C6, C7, C15, C16, C24 and C25), 7.91 (C1). MALDI: Calc. [M+K$^+$] = 6458.7 Da, Found [M+K$^+$] = 6462.39 Da.

G1-(PA-NHBoc)$_3$-(β-Ala-NHBoc)$_6$

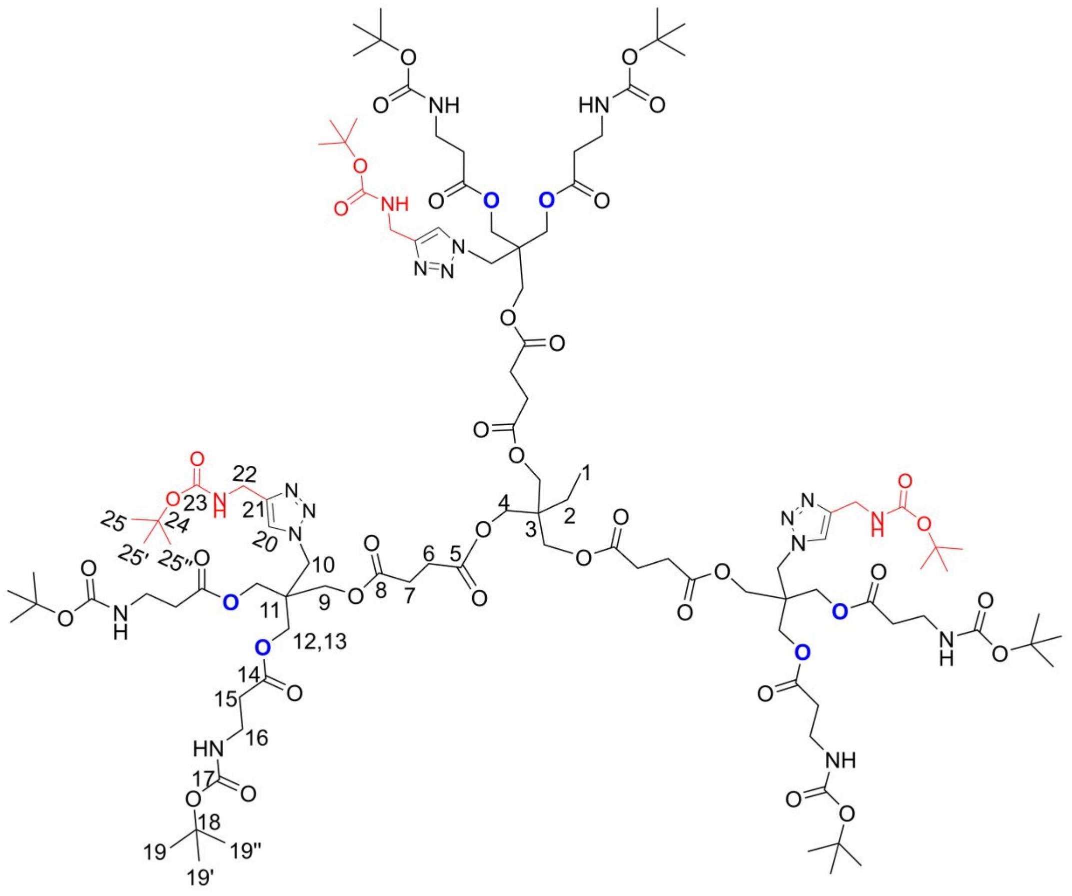

G1-(PA-NHBoc)$_3$-(β-Ala-NHBoc)$_6$ was synthesized following the general CDI mediated esterification procedure, utilizing the specified reagents with their indicated quantities: β-Ala-NHBoc (65.47 mg, 0.35 mmol), CDI (56.11 mg, 0.35 mmol), G1-(PA-NHBoc)$_3$-(OH)$_6$ (50.0 mg, 0.038 mmol) and CsF (7.0 mg, 0.046 mmol). (70.0 mg, 73%). $C_{105}H_{170}N_{18}O_{42}$ (2356.6 g mol^{-1}). ^{1}H-NMR (400 MHz, $CDCl_3$) δ/ppm: 7.61 (3H, s, H20), 5.39 (9H, m, br: NH), 4.48 (6H, s, H10), 4.35 (6H, m, H22), 4.04 (24H, m, H4, H9, H12 and H13), 3.38 (12H, m, H16), 2.63 (12H, m, H6, H7), 2.54 (12H, m, H15), 1.41 (83H, m, H2, H25, H25', H25'', H19, H19' and H19''), 0.87 (3H, m, H1). ^{13}C-NMR (101 MHz, $CDCl_3$) δ/ppm: 172.07 (C5, C8), 171.71 (C14), 156.01 (C17, C23), 145.77 (C21), 124.06 (C20), 79.78 (C24), 79.55 (C18), 64.30 (C4), 62.51 (C9), 62.24 (C12, C13), 49.59 (C10), 43.06 (C11), 40.90 (C3), 36.37 (C16), 36.06 (C22), 34.73 (C15), 29.80 (C6, C7), 28.90 (C6, C7), 28.51 (C25, C25', C25'', C19, C19' and C19''), 23.12 (C2), 7.48 (C1). MALDI: Calc. [M+Na$^+$] = 2379.58 Da, Found [M+Na$^+$] = 2379.14 Da. SEC (DMF) M_n = 3349.9 g mol^{-1}, M_w= 3445.5 g mol^{-1}, Đ = 1.02.

G1-(PA-NH$_3^+$)$_3$-(β-Ala-NH$_3^+$)$_6$

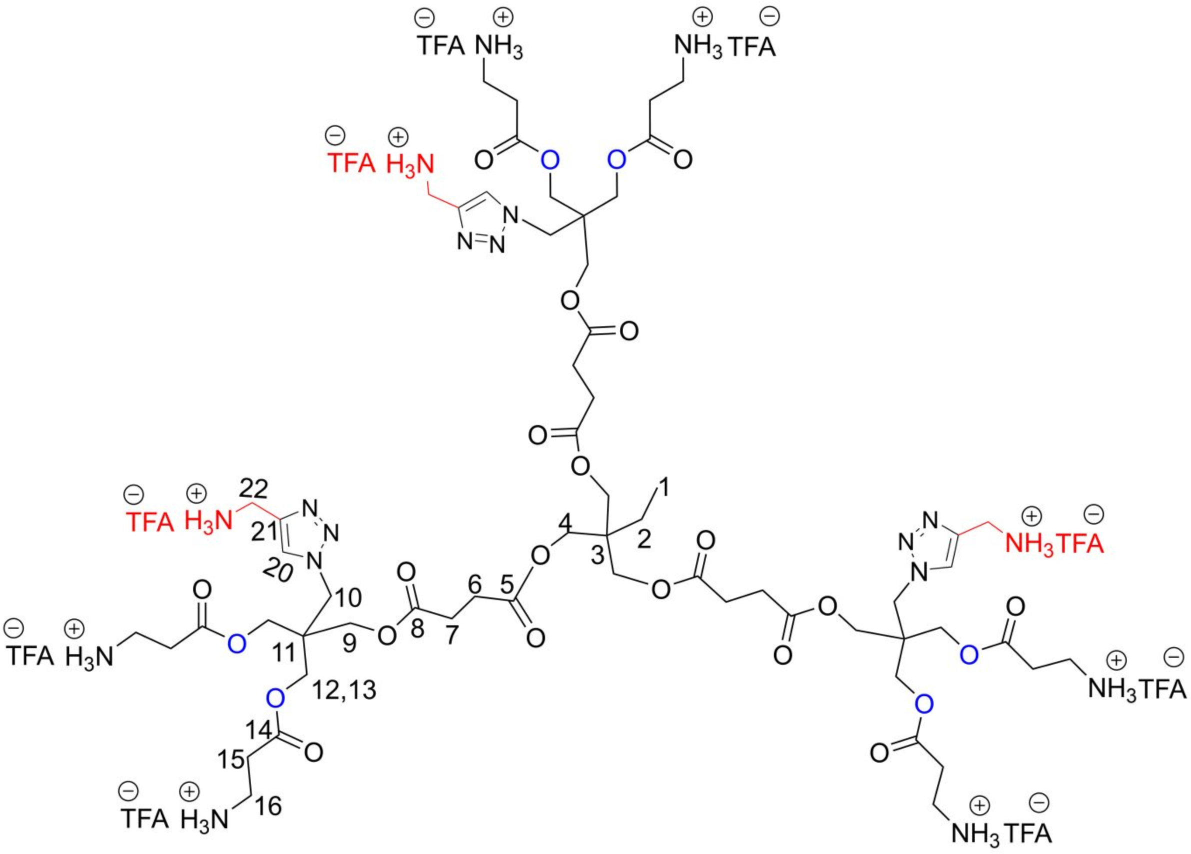

G1-(PA-NH$_3^+$)$_3$-(β-Ala-NH$_3^+$)$_6$ was synthesized following the general TFA deprotection procedure with a reaction time of 3 h, utilizing the specified reagents with their indicated quantities: G1-(PA-NHBoc)$_3$-(β-Ala-NHBoc)$_6$ (34.0 mg, 0.014 mmol), TFA (119.0 µL, 1.55 mmol) and DCM (119.0 µL). After precipitations in ether, G1-(PA)$_3$-(β-Ala-NH3+)$_6$ was freeze dried to obtain a white powder. (24.0 mg, 69%). $C_{78}H_{107}N_{18}O_{42}F_{27}$ (2481.79 g/mol). ^{1}H-NMR (400 MHz, MeOD) δ/ppm: 8.16 (3H, s, H20), 4.70 (6H, s, H10), 4.29 (6H, s, H22), 4.17 (18H, m, H9, H12 and H13), 4.07 (6H, s, H4), 3.25 (12H, t, H16), 2.84 (12H, t, H15), 2.67 (12H, m, H6, H7), 1.52 (2H, m, H2), 0.91 (3H, m, H1). ^{13}C-NMR (101 MHz, MeOD) δ/ppm: 173.91 (C5), 173.53 (C8), 171.71 (C14), 163.57 (C=O, TFA), 163.23 (C=O, TFA), 162.88 (C=O, TFA), 141.38 (C21), 127.60 (C20), 119.68 (CF_3, TFA), 116.76 (CF_3, TFA), 65.29 (C4), 64.04 (C12, C13), 63.61 (C9), 50.80 (C10), 44.17 (C11), 42.13 (C3), 36.27 (C22), 35.42 (C16), 32.16 (C15), 29.75 (C6, C7), 29.71 (C6, C7), 24.01 (C2), 7.72 (C1). MALDI: Calc. [M+Na$^+$] = 1487.60 Da, Found [M+Na$^+$] = 1477.77 Da.

G2-(PA-NHBoc)$_9$-(β-Ala-NHBoc)$_{12}$

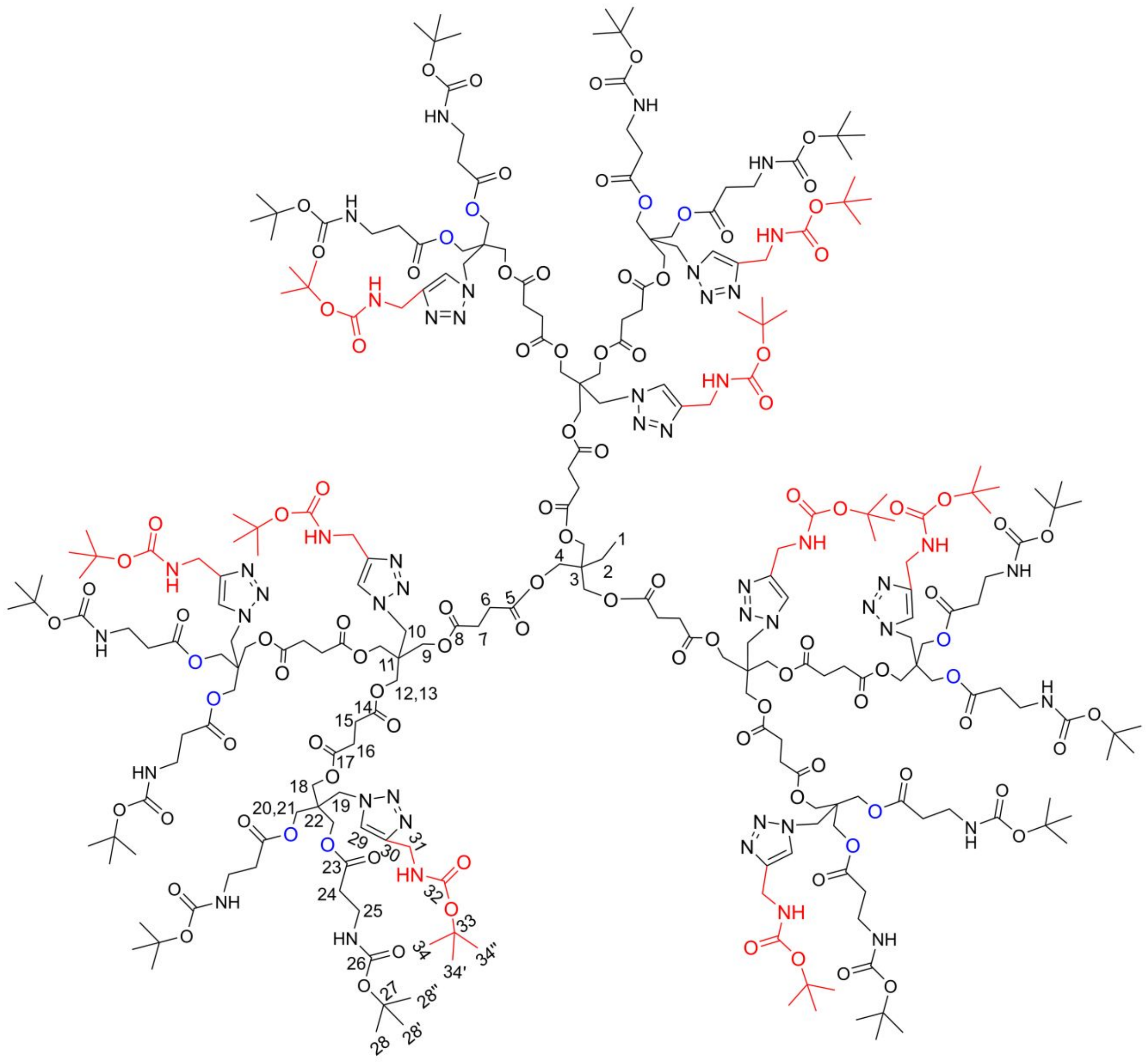

G2-(PA-NHBoc)$_9$-(β-Ala-NHBoc)$_{12}$ was synthesized following the general CDI mediated esterification procedure, utilizing the specified reagents with their indicated quantities: β-Ala-NHBoc (80 mg, 0.42 mmol), CDI (68.6 mg, 0.42 mmol), G2-(PA-NHBoc)$_9$-(OH)$_{12}$ (70.0 mg, 0.018 mmol) and CsF (7.0 mg, 0.045 mmol). (65.0 mg, 63%). $C_{255}H_{404}N_{48}O_{102}$ (5774.27 g/mol). ^{1}H-NMR (400 MHz, $CDCl_3$) δ/ppm: 7.65 (9H, m, H29), 5.49 (21H, m, br: NH), 4.50 (18H, m, H10, H19), 4.34 (18H, m, H31), 4.06 (62H, m, H4, H9, H12, H13, H18, H20 and H21), 3.37 (24H, m, H25), 2.63 (36H, m, H6, H7, H15 and H16), 2.54 (24H, m, H24), 1.40 (191H, m, H2, H34, 34', H34'', H28, H28' and H28''), 0.85 (3H, m, H1). ^{13}C-NMR (101 MHz, $CDCl_3$) δ/ppm: 172.10 (C5, C8, C14 and C17), 171.72 (C23), 156.03 (C26, C32), 145.73 (C30), 124.12 (C29), 79.73 (C33), 79.53 (C27), 64.25 (C4), 62.91 (C9, C12), 62.69 (C13, C18), 62.28 (C20, C21), 49.62 (C10, C19), 43.02 (C11), 42.92 (C22), 40.86 (C3), 36.36 (C25), 36.04 (C31), 34.71 (C24), 28.87 (C6, C7, C15 and C16), 28.50 (C34, C34', 34'', C28, C28' and C28''), 7.49 (C1). MALDI: Calc. [M+Cu$^+$] = 5837.81 Da, Found [M+Cu$^+$] = 5838.42 Da. SEC (DMF) M_n = 6917.3 g mol^{-1}, M_w= 7130.3 g mol^{-1}, Đ = 1.03.

G2-(PA-NH_3^+)$_9$-(β-Ala-NH_3^+)$_{12}$

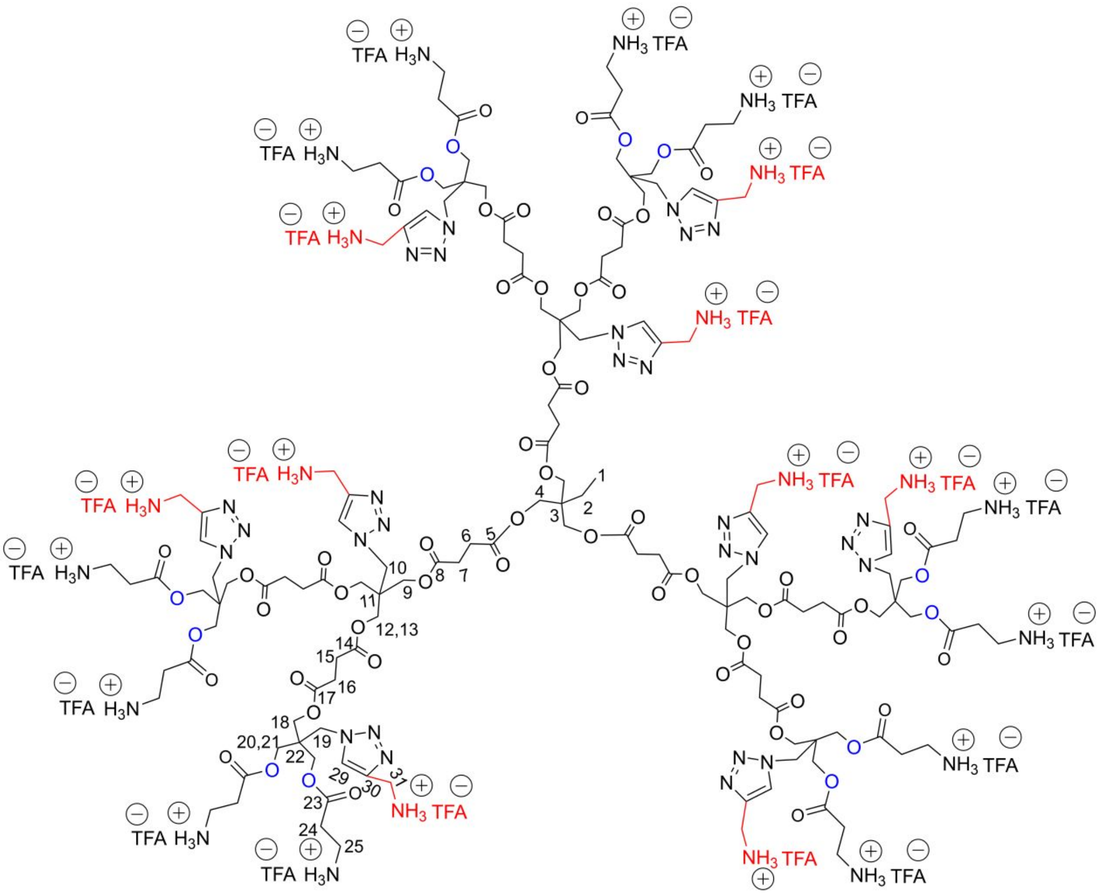

G2-(PA-NH_3^+)$_9$-(β-Ala-NH_3^+)$_{12}$ was synthesized following the general TFA deprotection procedure with a reaction time of 7 h, utilizing the specified reagents with their indicated quantities: G2-(PA-NHBoc)$_9$-(β-Ala-NHBoc)$_{12}$ (42.0 mg, 0.007 mmol), TFA (140.0 µL, 1.83 mmol) and DCM (140.0 µL). After precipitations in ether, G2-(PA)$_9$-(β-Ala-NH_3^+)$_{12}$ was freeze dried to obtain a white powder. (24.0 mg, 57%). $C_{192}H_{257}N_{48}O_{102}F_{63}$ (6066.39 g/mol). ^{1}H-NMR (400 MHz, MeOD) δ/ppm: 8.16 (9H, m, H29), 4.69 (18H, m, H10, H19), 4.31 (18H, m, H31), 4.13 (60H, m, H4, H9, H12, H13, H18, H20 and H21), 3.26 (24H, t, H25), 2.84 (24H, t, H24), 2.68 (36H, m, H6, H7, H15 and H16), 1.51 (2H, m, H2), 0.89 (3H, m, H1). ^{13}C-NMR (101 MHz, MeOD) δ/ppm: 173.92 (C5, C8), 173.63 (C14), 173.61 (C17), 171.75 (C23), 163.54 (C=O, TFA), 163.20 (C=O, TFA), 162.86 (C=O, TFA), 162.51 (C=O, TFA), 141.40 (C31), 127.67 (C30), 119.67 (CF_3, TFA), 116.76 (CF_3, TFA), 64.07 (C4, C9, C12, C13, C18, C20 and C21), 63.75 (C4, C9, C12, C13, C18, C20 and C21), 50.83 (C10, C19), 44.15 (C11, C22), 36.29 (C31), 35.45 (C25), 32.18 (C24), 29.69 (C6, C7, C15 and C16), 7.81 (C1). MALDI: Calc. [M+Na^+] = 3715.95 Da, Found [M+Na^+] = 3715.01 Da.

G3-(PA-NHBoc)$_{21}$-(β-Ala-NHBoc)$_{24}$

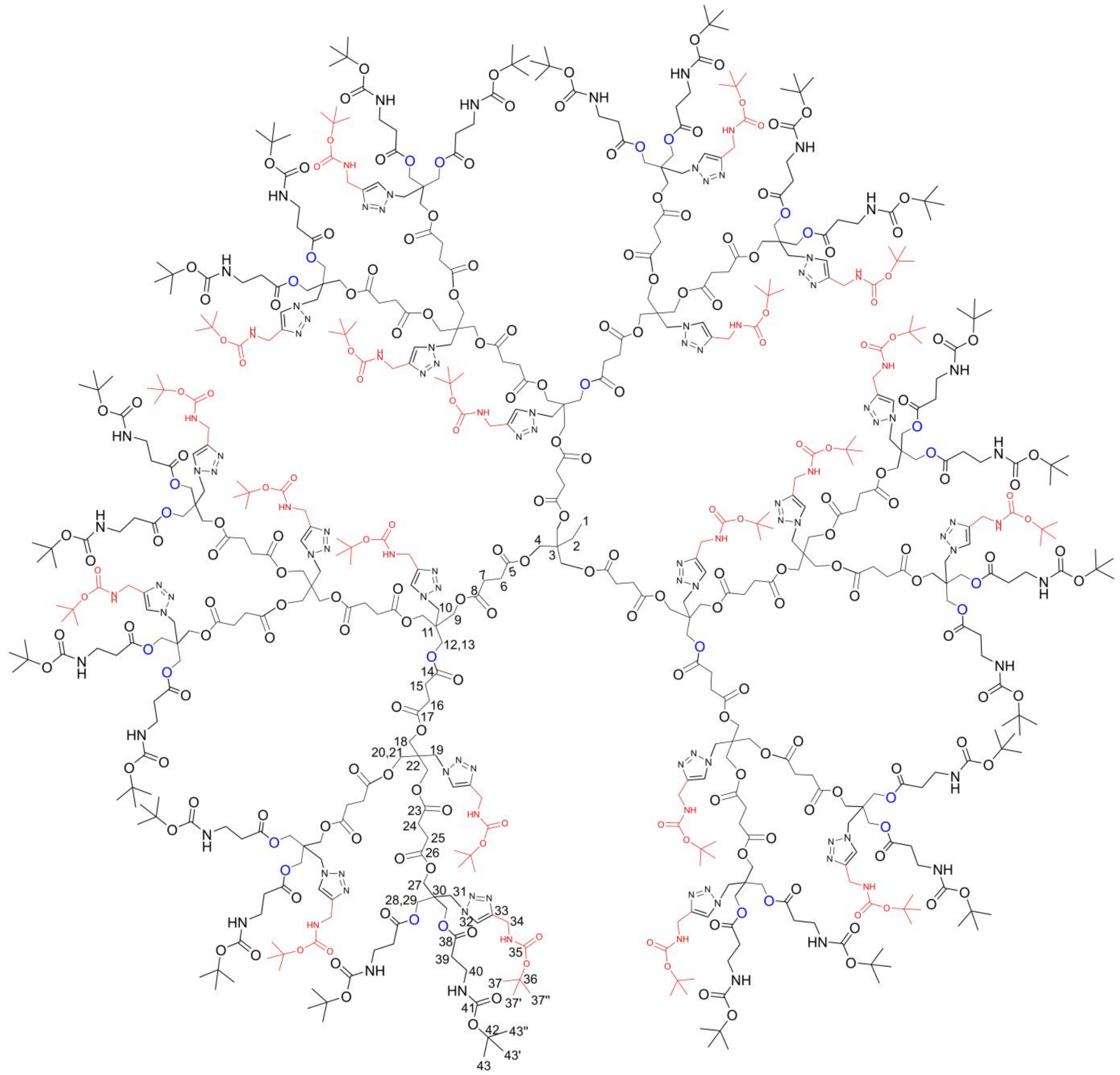

G3-(PA-NHBoc)$_{21}$-(β-Ala-NHBoc)$_{24}$ was synthesized following the general anhydride-based esterification procedure, utilizing the specified reagents with their indicated quantities: β-Ala-NHBoc (60.0 mg, 0.32 mmol), DCC (32.7 mg, 0.16 mmol), β-Ala-NHBoc anhydride (56.47 mg, 0.16 mmol), G3-(PA-NHBoc)$_{21}$-(OH)$_{24}$ (37.0 mg, 0.004 mmol), DMAP (2.6 mg, 0.021 mmol) and Pyridine (42.0 uL, 0.52 mmol). (18.2 mg, 37%). $C_{555}H_{872}N_{108}O_{222}$ (12609.62 g/mol). ^{1}H-NMR (400 MHz, $CDCl_3$) δ/ppm: 7.67 (21H, m, H32), 5.50 (45H, m, br: NH) 4.51 (42H, m, H10, H19 and H31), 4.36 (42H, m, H34), 4.08 (132H, m, H4, H9, H12, H13, H18, H20, H21, H27, H28 and H29), 3.38 (48H, m, H40), 2.60 (132H, m, H6, H7, H15, H16, H24, H25 and H39), 1.41 (407H, m, H2, H37, H37', H37'', H43, H43' and H43''), 0.86 (3H, m, H1). ^{13}C-NMR (101 MHz, $CDCl_3$) δ/ppm: 172.09 (C5, C8 and C14), 171.76 (C17, C23 and C26), 171.53 (C38), 156.05 (C35, C41), 145.69 (C33), 124.14 (C32), 79.58 (C36, C42), 65.98 (C4), 62.88 (C9, C12 and C13), 62.70 (C18, C20 and C21), 62.52 (C27), 62.28 (C28 and C29), 49.74 (C10, C19 and C31), 43.07 (C11, C22), 43.00 (C30), 40.94 (C3), 36.40 (C40), 36.09 (C34), 34.75 (C39), 28.92 (C6, C7, C15, C16, C24 and C25), 28.88 (C37, 37', C37'', C43, C43' and C43''), 28.54 (C37, 37', C37'', C43, C43' and C43''), 22.82 (C2), 7.51 (C1).

G3-(PA-NH_3^+)$_{21}$-(β-Ala-NH_3^+)$_{24}$

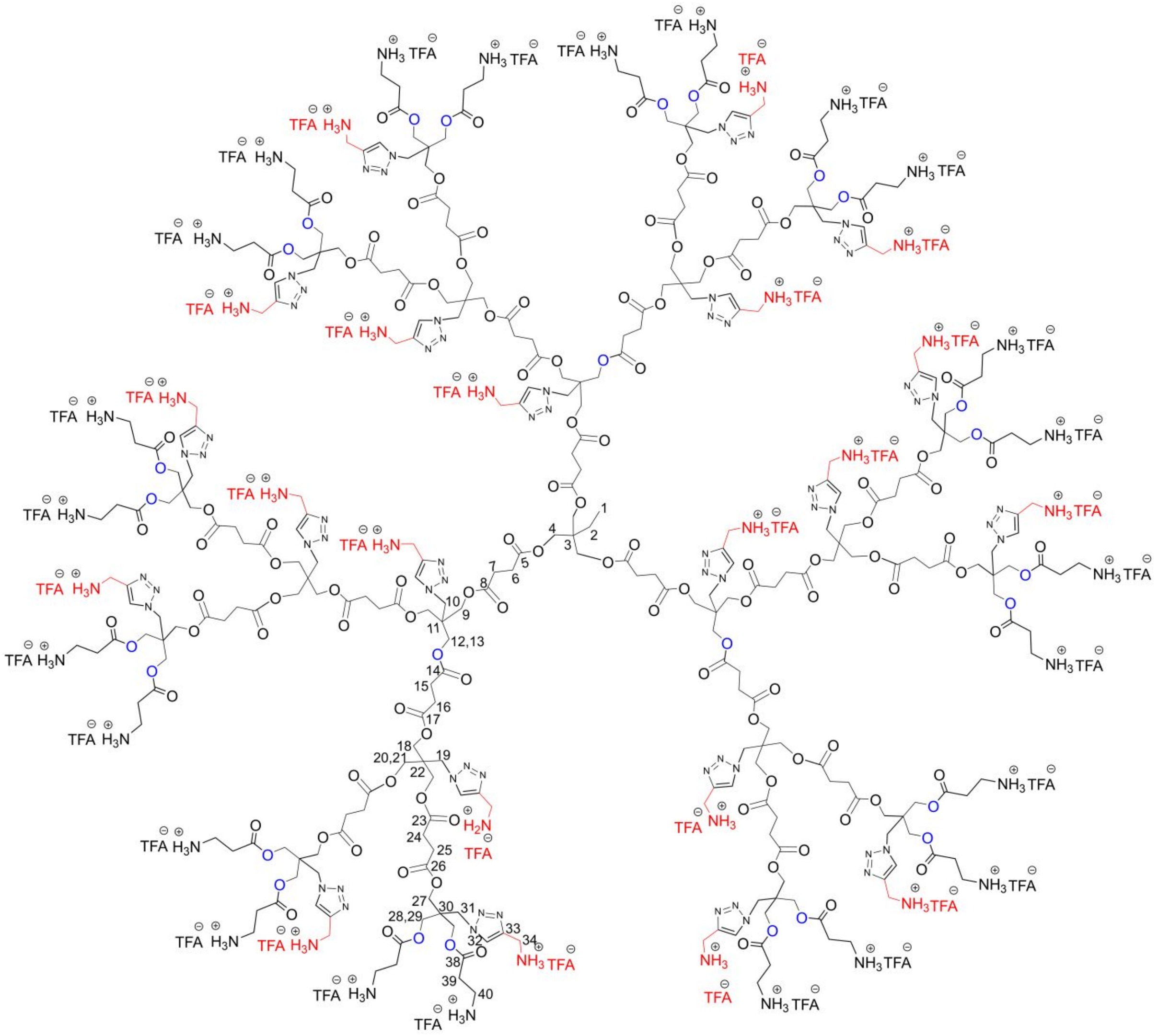

G3-(PA-NH_3^+)$_{21}$-(β-Ala-NH_3^+)$_{24}$ was synthesized following the general TFA deprotection procedure with a reaction time of 8 h, utilizing the specified reagents with their indicated quantities: G3-(PA-NHBoc)$_{21}$-(β-Ala-NHBoc)$_{24}$ (18.2 mg, 0.001 mmol), TFA (60.0 µL, 0.78 mmol) and DCM (60.0 µL). After precipitations in ether, G3-(PA-NH_3^+)$_{21}$-(β-Ala-NH_3^+)$_{24}$ was freeze dried to obtain a white powder. (10.0 mg, 76%). $C_{420}H_{557}N_{108}O_{222}F_{135}$ (13235.59 g/mol). ^{1}H-NMR (400 MHz, MeOD) δ/ppm: 8.16 (21H, m, H32), 4.70 (42H, m, H10, H19 and H31), 4.30 (42H, m, H34), 4.12 (132H, m, H4, H9, H12, H13, H18, H20, H21, H27, H28 and H29), 3.26 (48H, m, H40), 2.75 (132H, m, H39, H6, H7, H15, H16, H24 and H25), 1.51 (2H, m, H2), 0.90 (3H, m, H1). ^{13}C-NMR (101 MHz, MeOD) δ/ppm: 173.63 (C5, C8, C14, C17, C23 and C26), 171.75 (C38), 163.01 (C=O, TFA), 162.65 (C=O, TFA), 162.30 (C=O, TFA), 161.95 (C=O, TFA), 141.38 (C33), 127.65 (C32), 120.29 (CF_3, TFA), 119.41 (CF_3, TFA), 117.46 (CF_3, TFA), 116.49 (CF_3, TFA), 114.64 (CF_3, TFA), 111.81 (CF_3, TFA), 64.06 (C4, C9, C12, C13, C18, C20, C21, C27, C28 and C29), 49.71 (C10, C19 and C31), 44.16 (C11, C22 and C30), 36.30 (C34), 35.46 (C40), 32.18 (C39), 29.70 (C6, C7, C15, C16, C24 and C25).

Figures

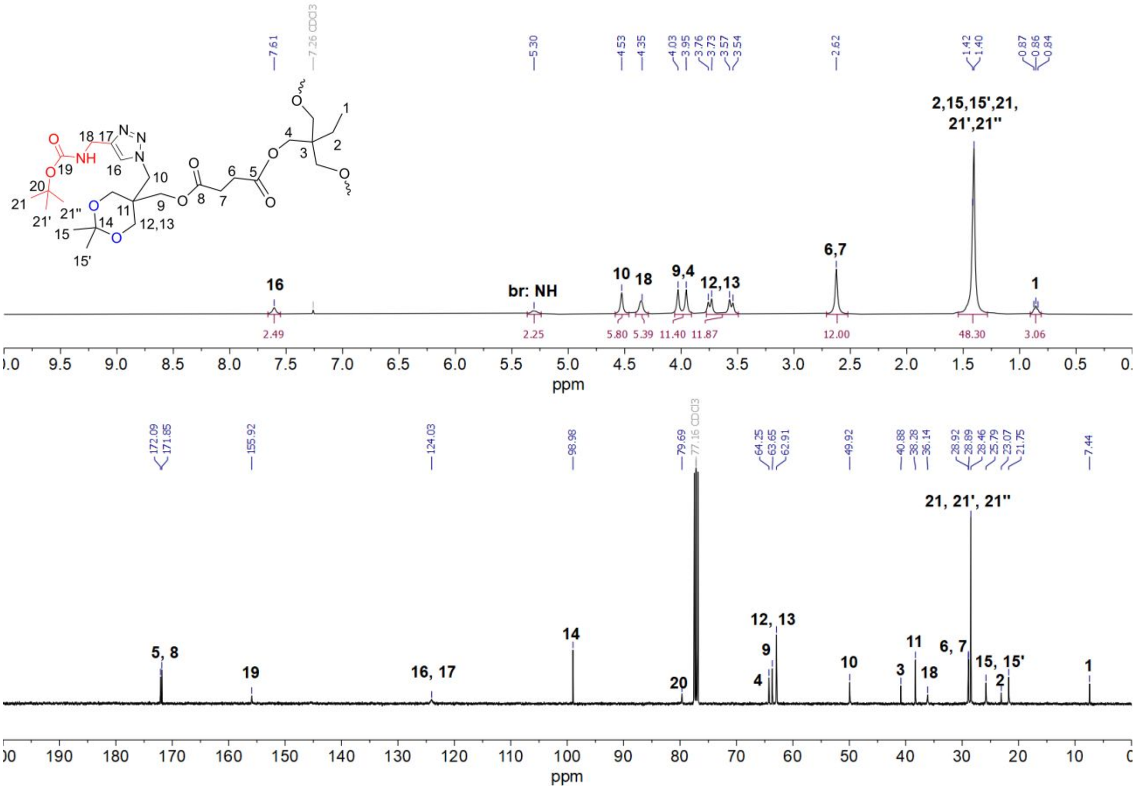

Figure S1. ¹H and ¹³C NMR spectra of G1-(PA-NHBoc)$_3$-(Ac)$_3$ in $CDCl_3$.

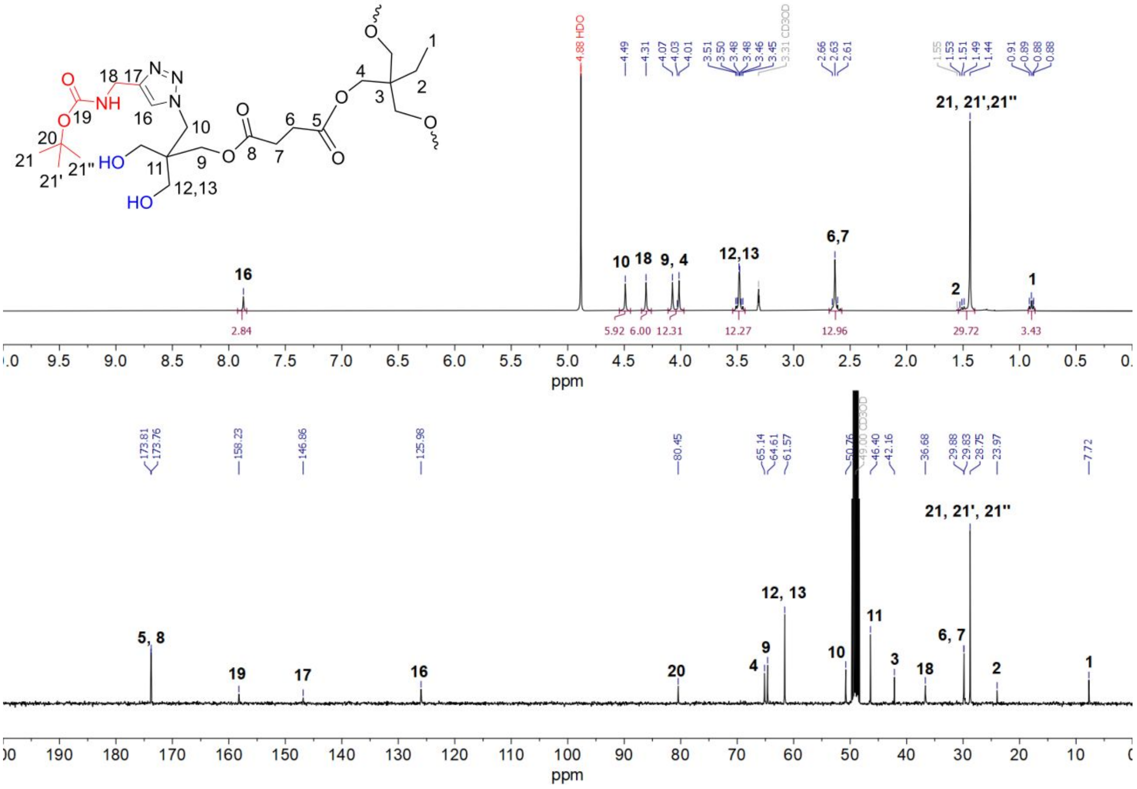

Figure S2. ¹H and ¹³C NMR spectra of G1-(PA-NHBoc)$_3$-(OH)$_6$ in CD_3OD.

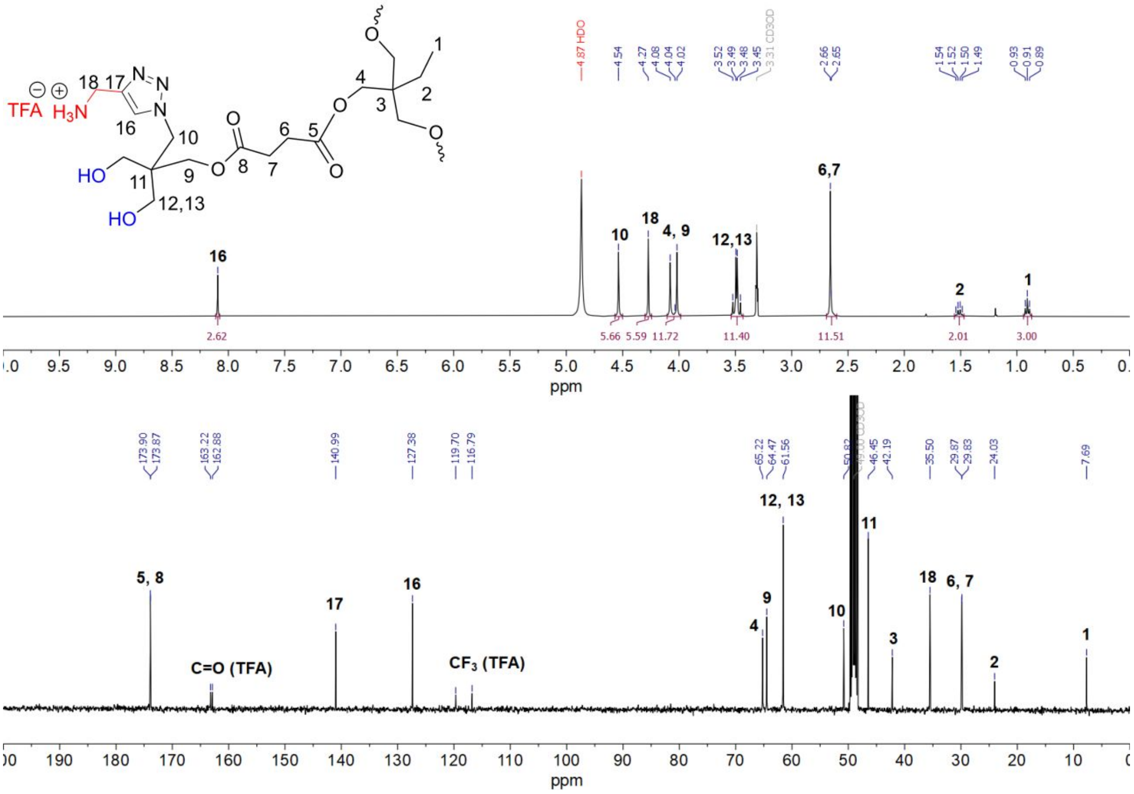

Figure S3. ^{1}H and ^{13}C NMR spectra of G1-(PA-NH_3^+)$_3$-(OH)$_6$ in CD_3OD.

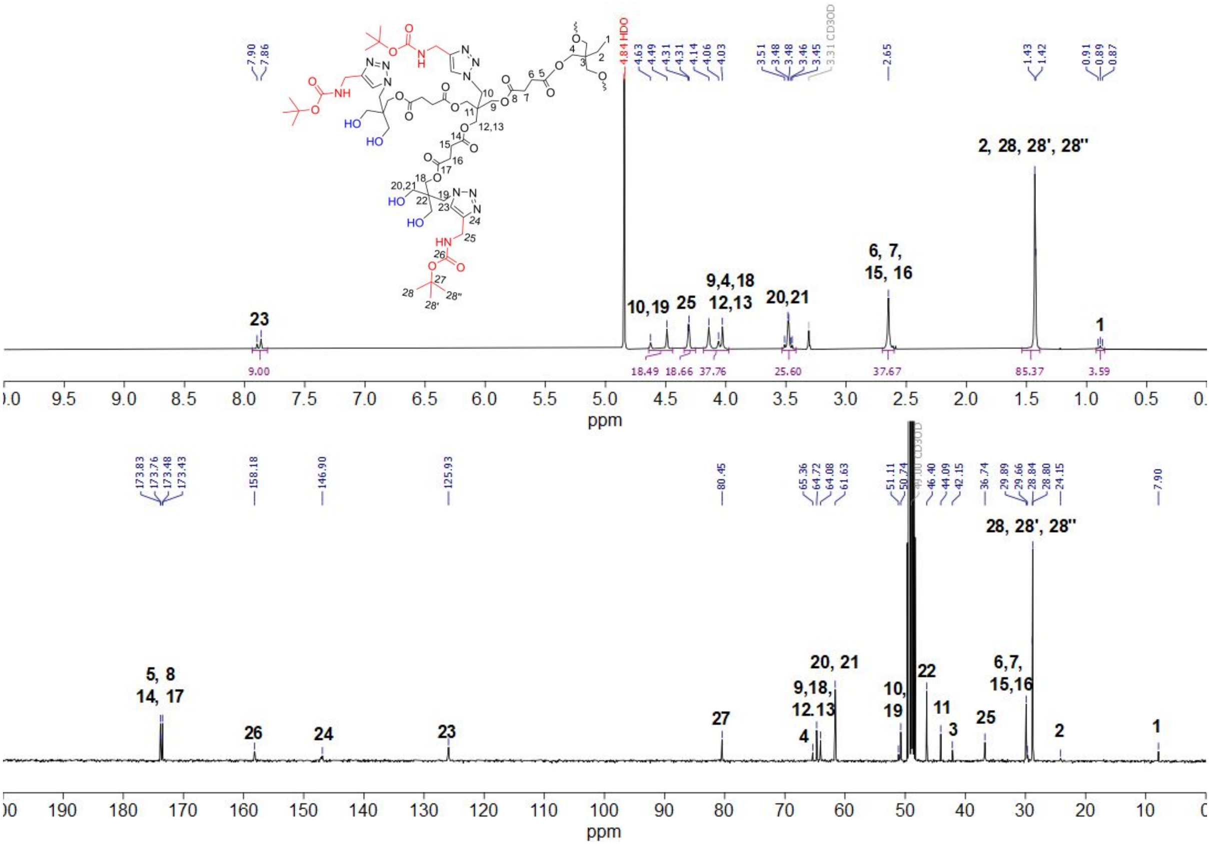

Figure S4. ^{1}H and ^{13}C NMR spectra of G2-(PA-NHBoc)$_9$-(OH)$_{12}$ in CD_3OD.

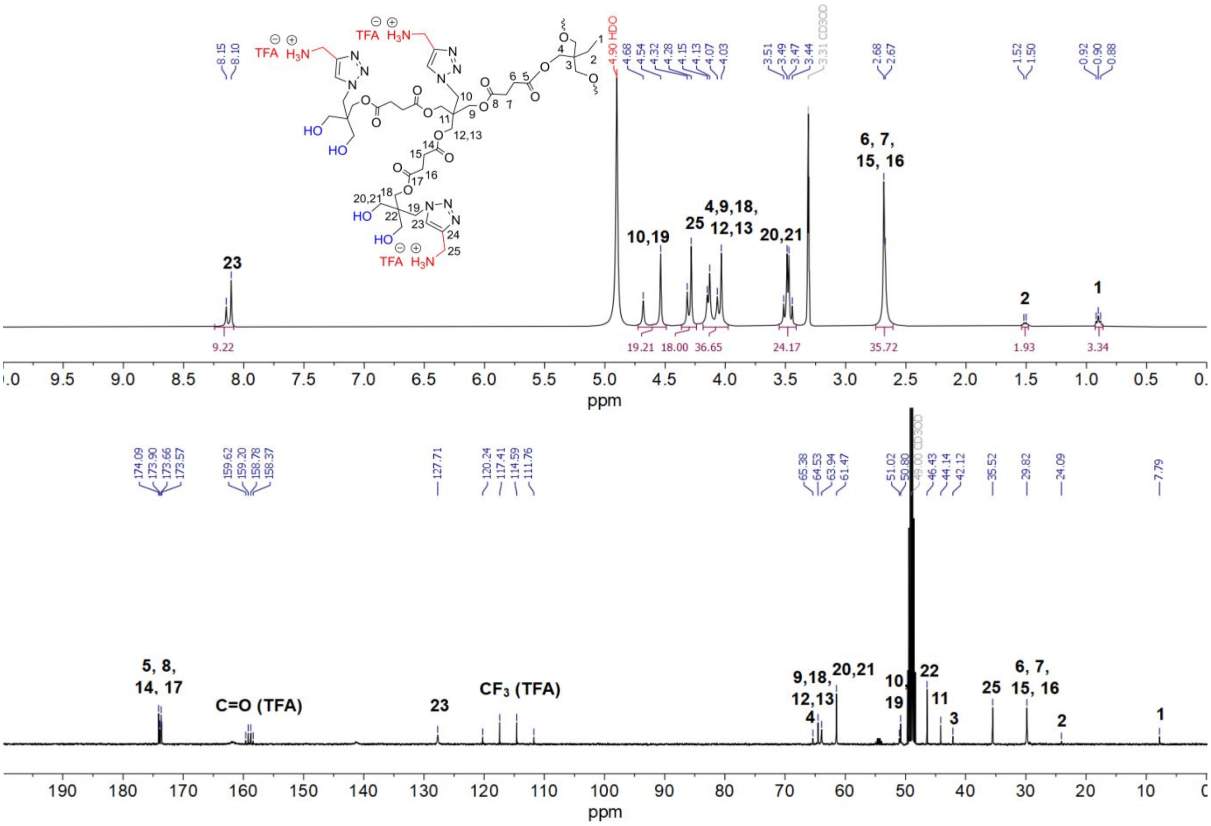

Figure S5. ^{1}H and ^{13}C NMR spectra of G2-$(PA-NH_3^+)_9$-$(OH)_{12}$ in CD_3OD.

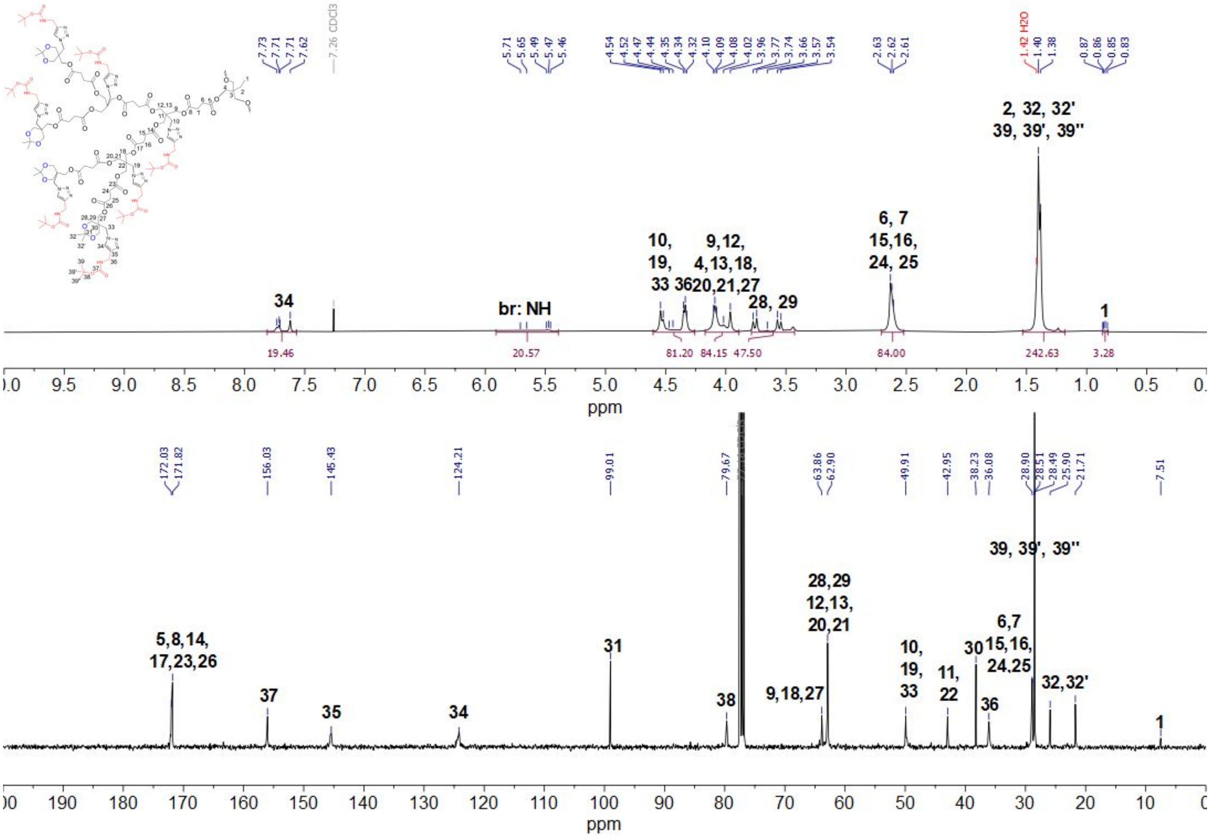

Figure S6. ^{1}H and ^{13}C NMR spectra of G3-$(PA-NHBoc)_{21}$-$(Ac)_{12}$ in $CDCl_3$.

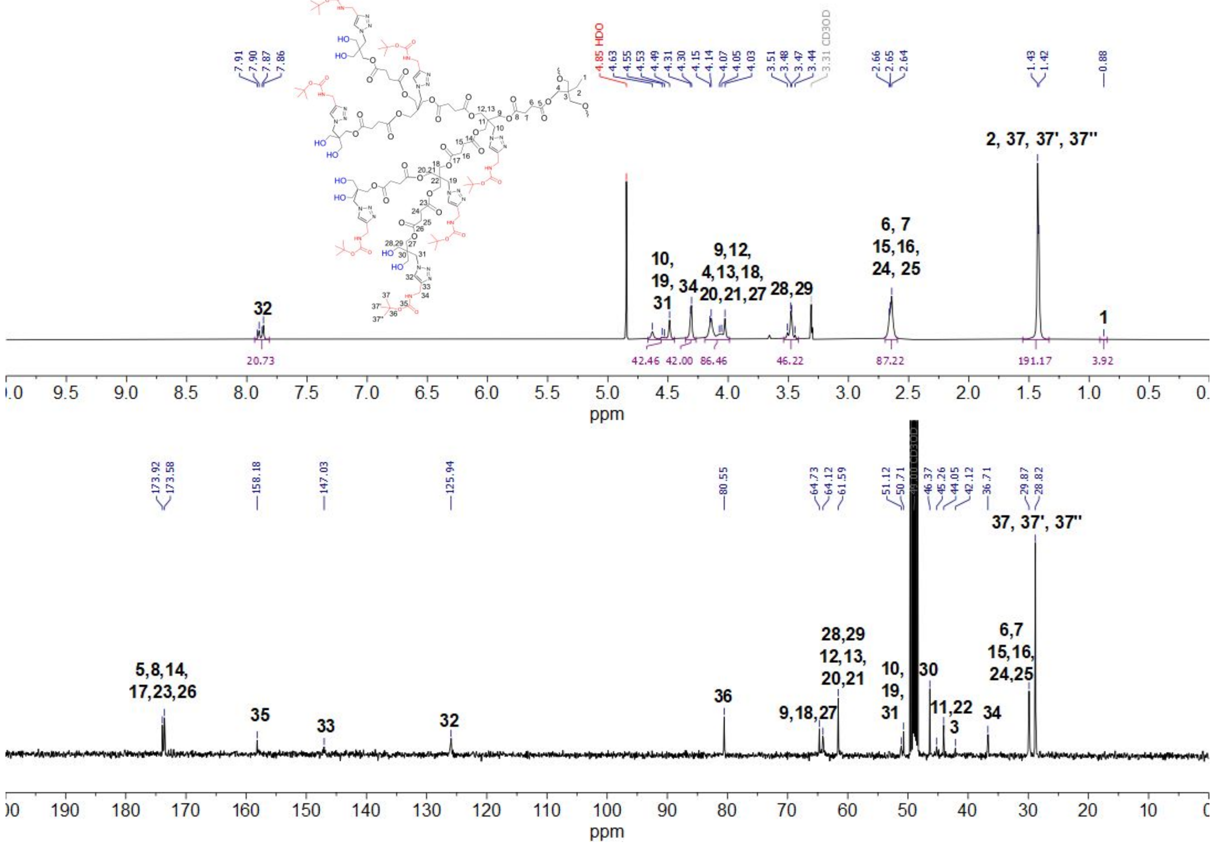

Figure S7. ^{1}H and ^{13}C NMR spectra of G3-(PA-NHBoc)$_{21}$-(OH)$_{24}$ in CD_3OD.

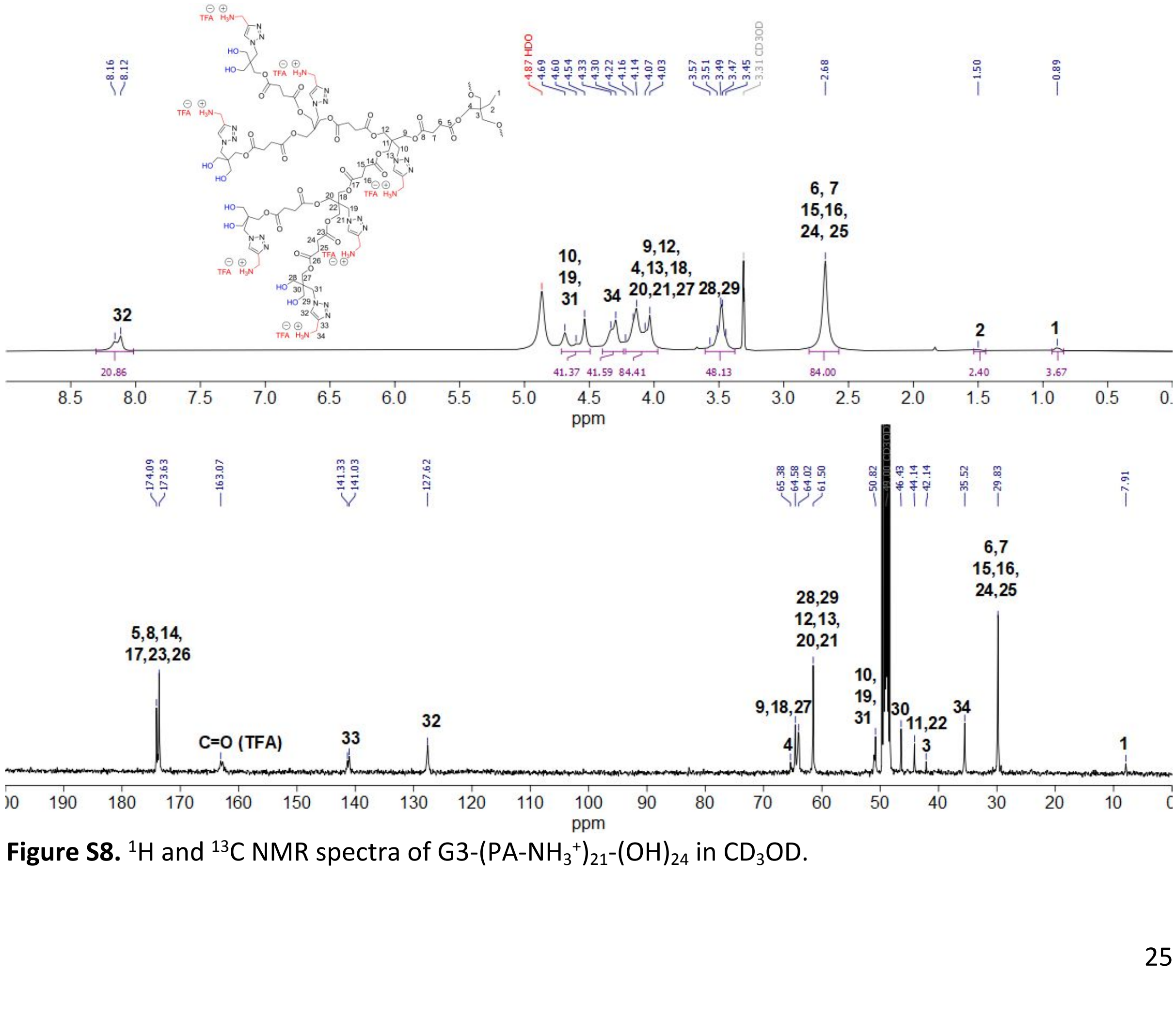

Figure S8. ^{1}H and ^{13}C NMR spectra of G3-(PA-NH_3^+)$_{21}$-(OH)$_{24}$ in CD_3OD.

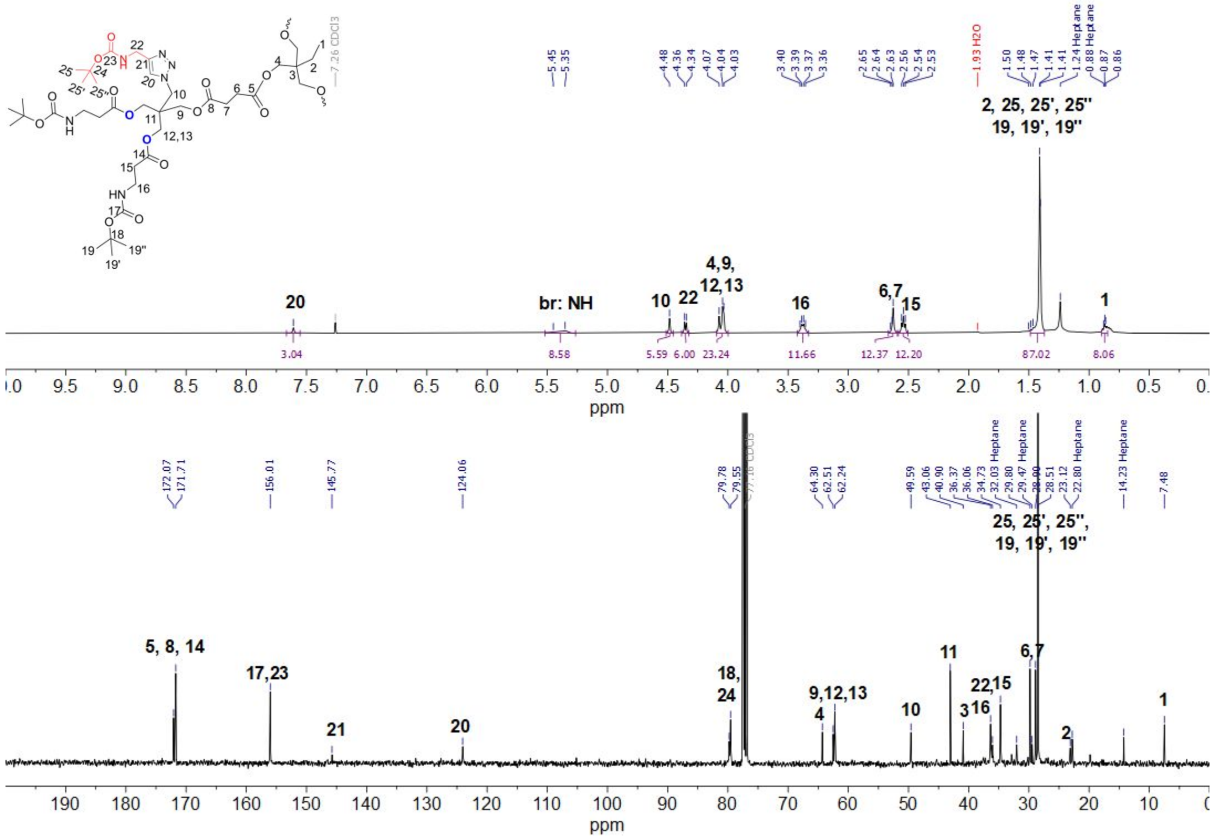

Figure S9. ^{1}H and ^{13}C NMR spectra of G1-(PA-NHBoc)$_3$-(β-Ala-NHBoc)$_6$ in $CDCl_3$. Heptane is denoted as residual solvent.

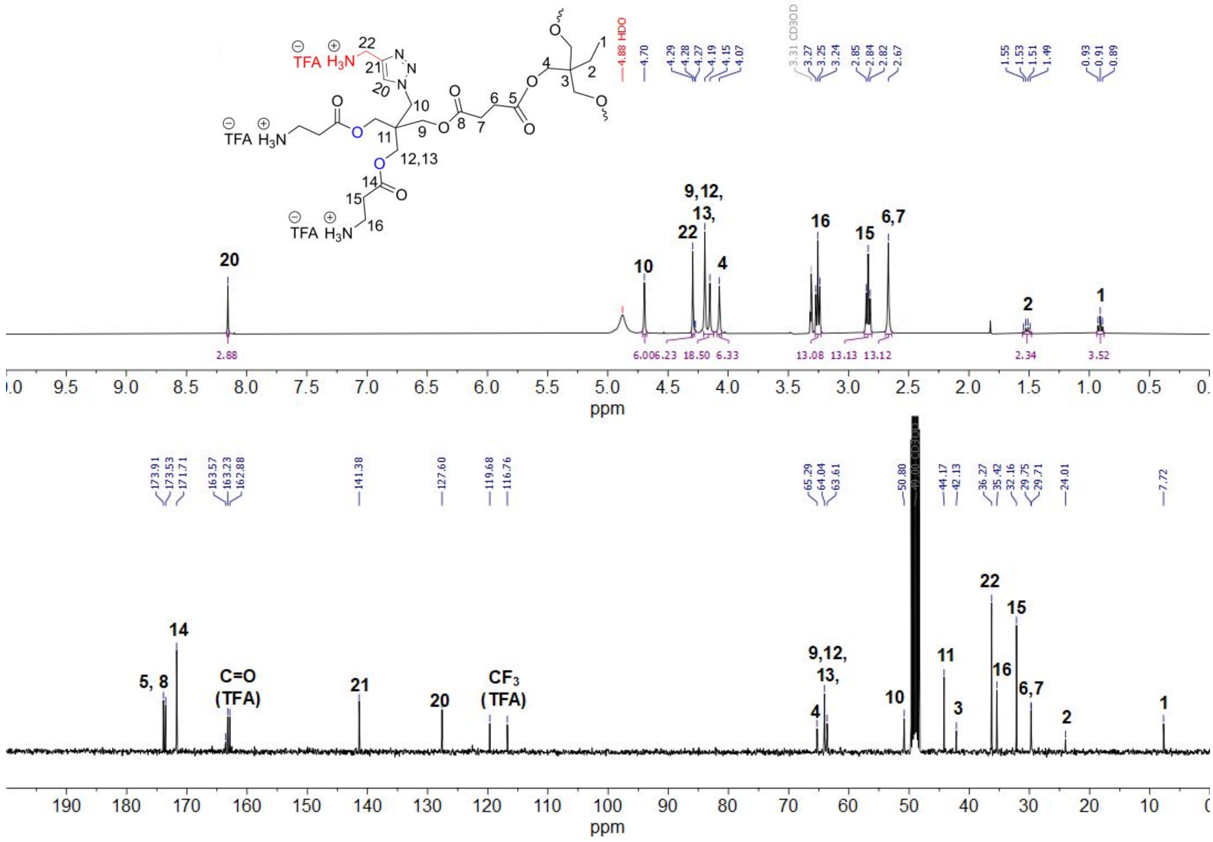

Figure S10. ^{1}H and ^{13}C NMR spectra of G1-(PA-NH_3^+)$_3$-(β-Ala-NH_3^+)$_6$ in CD_3OD.

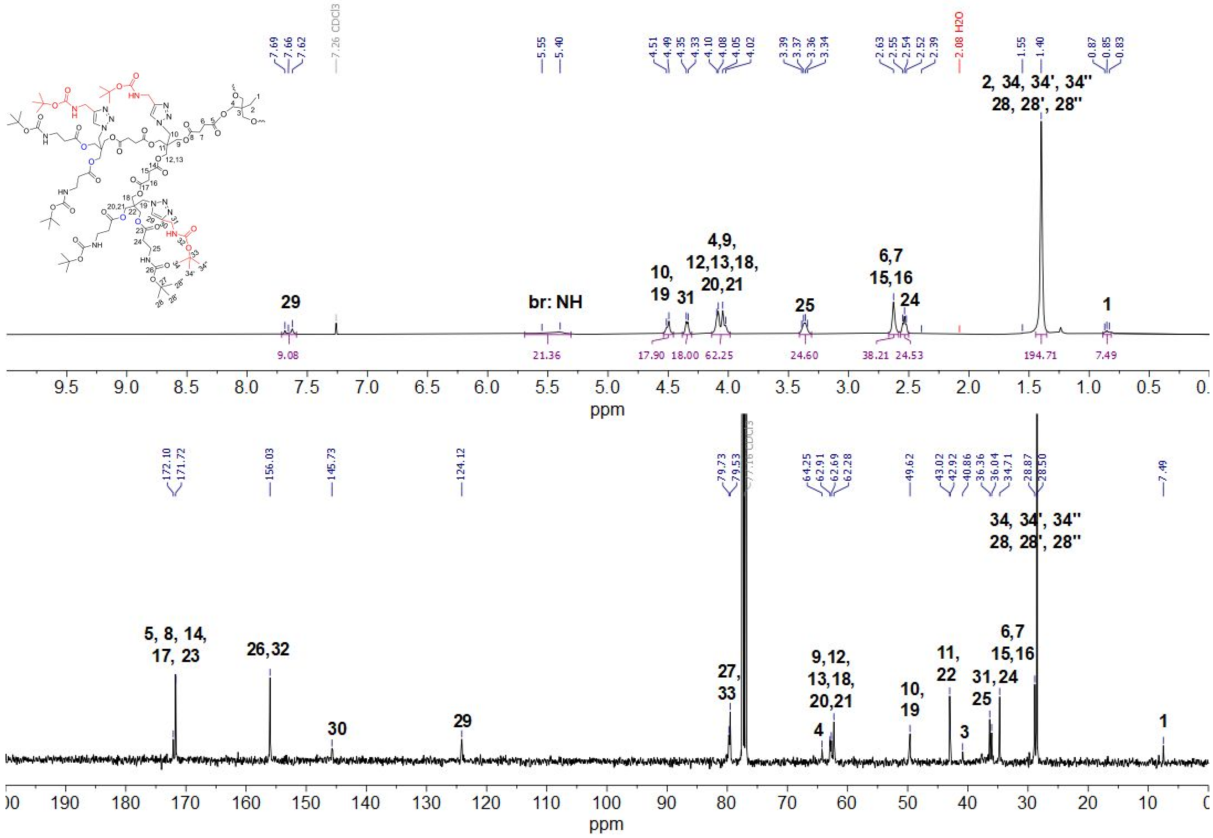

Figure S11. ^{1}H and ^{13}C NMR spectra of G2-(PA-NHBoc)$_9$-(β-Ala-NHBoc)$_{12}$ in $CDCl_3$.

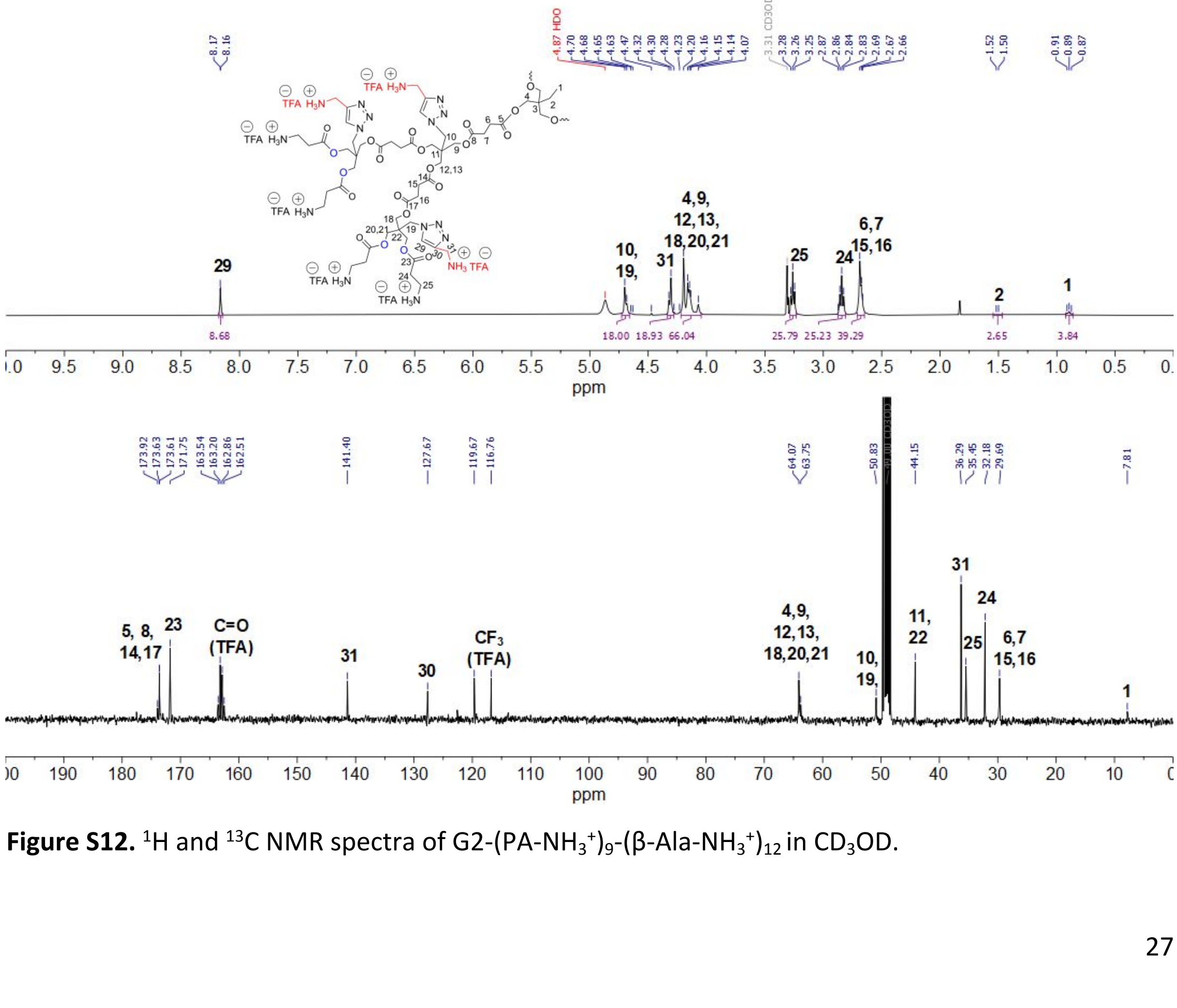

Figure S12. ^{1}H and ^{13}C NMR spectra of G2-(PA-NH_3^+)$_9$-(β-Ala-NH_3^+)$_{12}$ in CD_3OD.

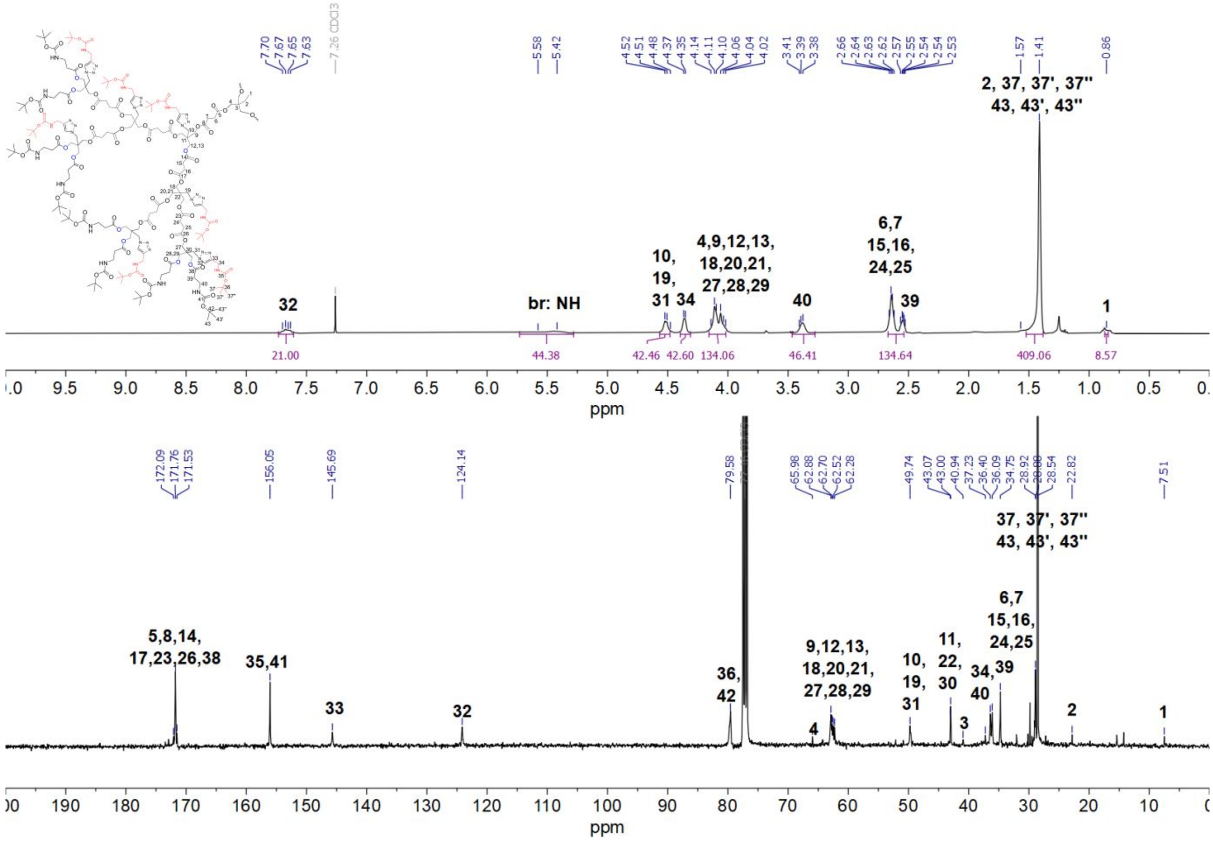

Figure S13. ^{1}H and ^{13}C NMR spectra of G3-(PA-NHBoc)$_{21}$-(β-Ala-NHBoc)$_{24}$ in $CDCl_3$.

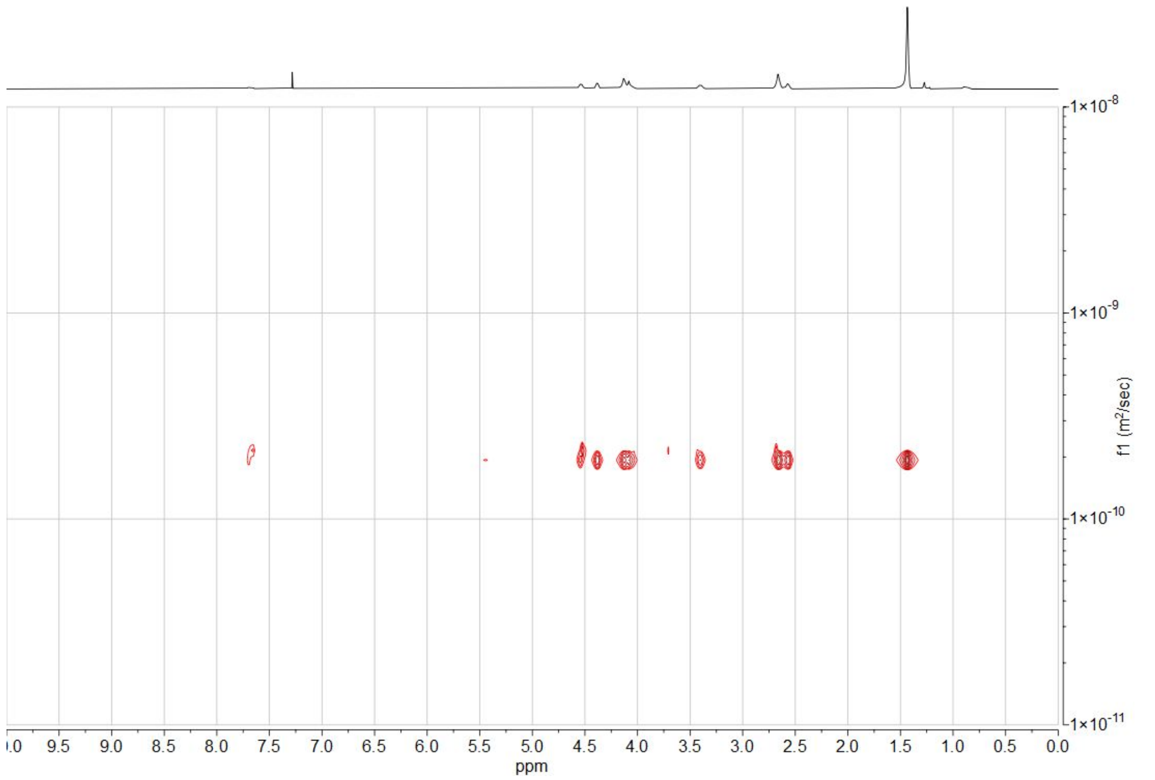

Figure S14. DOSY spectra of G3-(PA-NHBoc)$_{21}$-(β-Ala-NHBoc)$_{24}$ in $CDCl_3$.

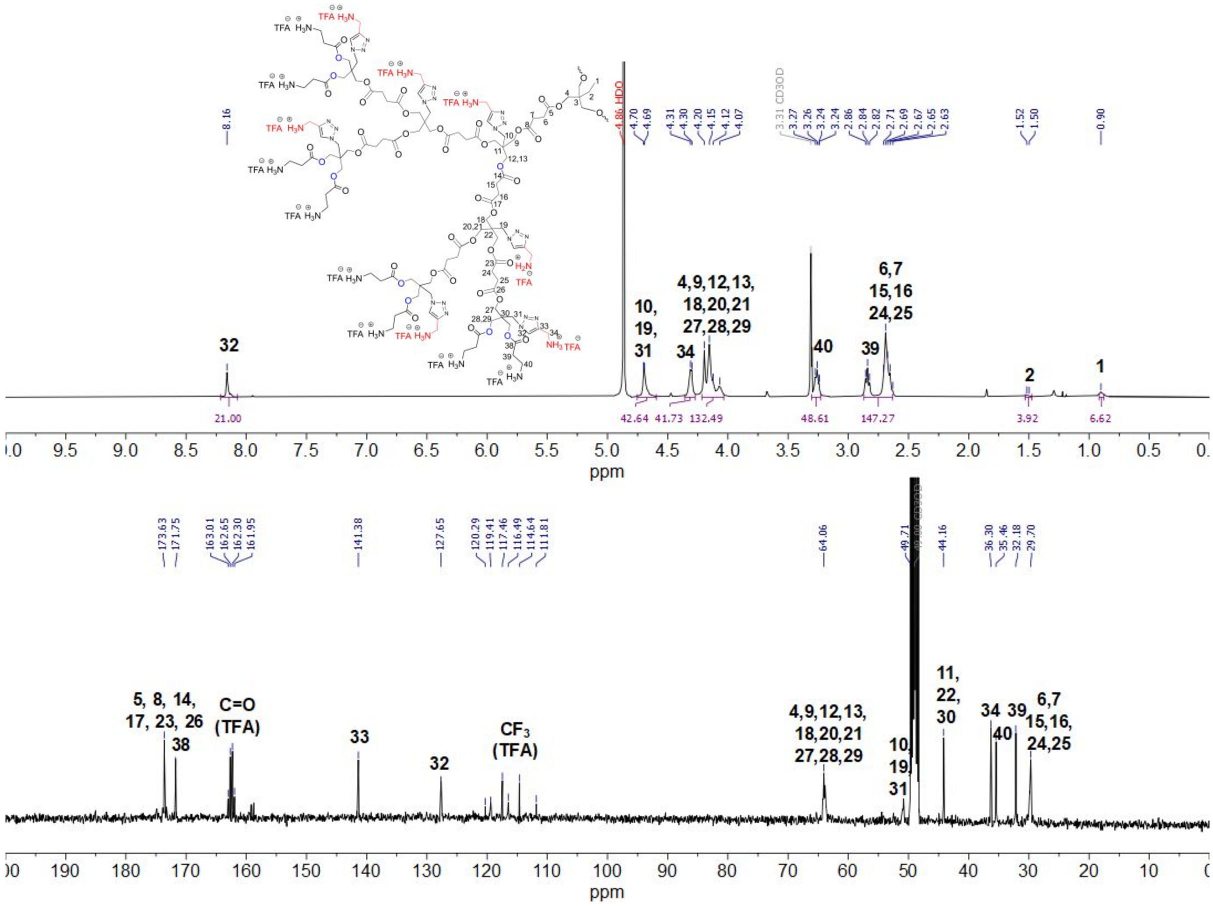

Figure S15. ^{1}H and ^{13}C NMR spectra of G3-$(PA\text{-}NH_3^+)_{21}$-$(\beta\text{-}Ala\text{-}NH_3^+)_{24}$ in CD_3OD.

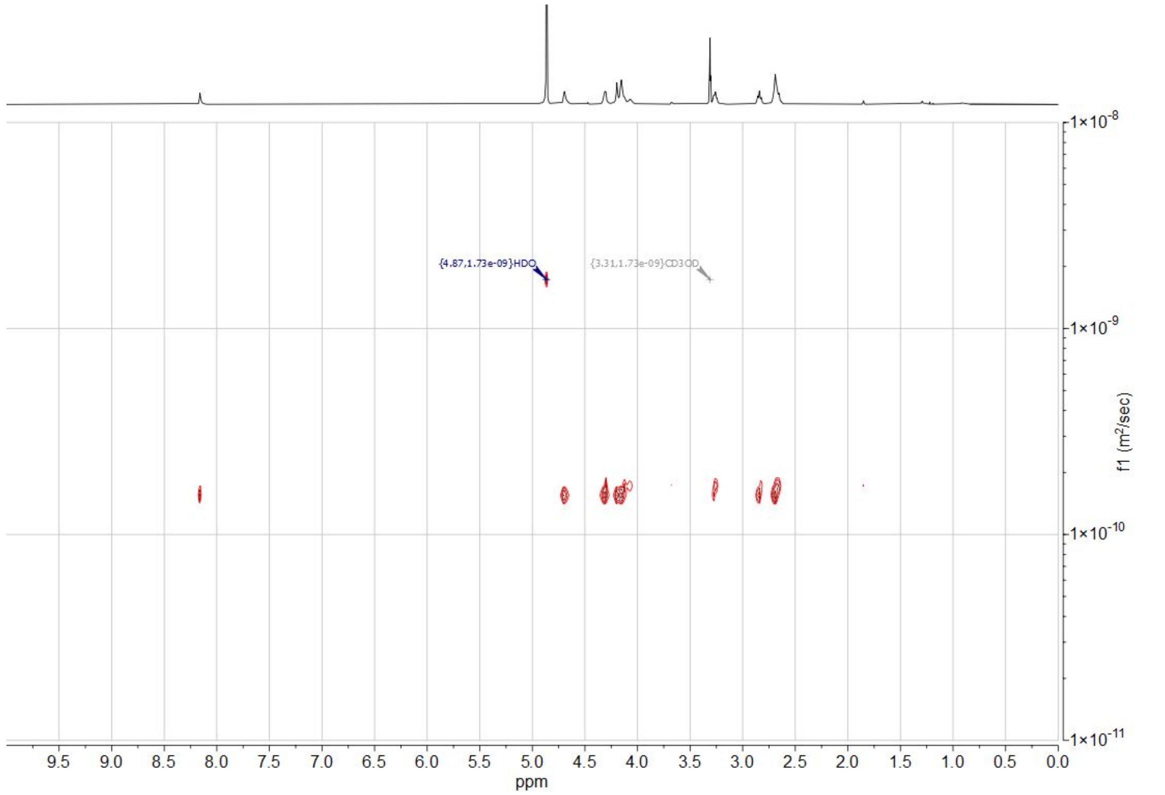

Figure S16. DOSY spectra of G3-(PA-NH_3^+)$_{21}$-(β-Ala-NH_3^+)$_{24}$ in CD_3OD.

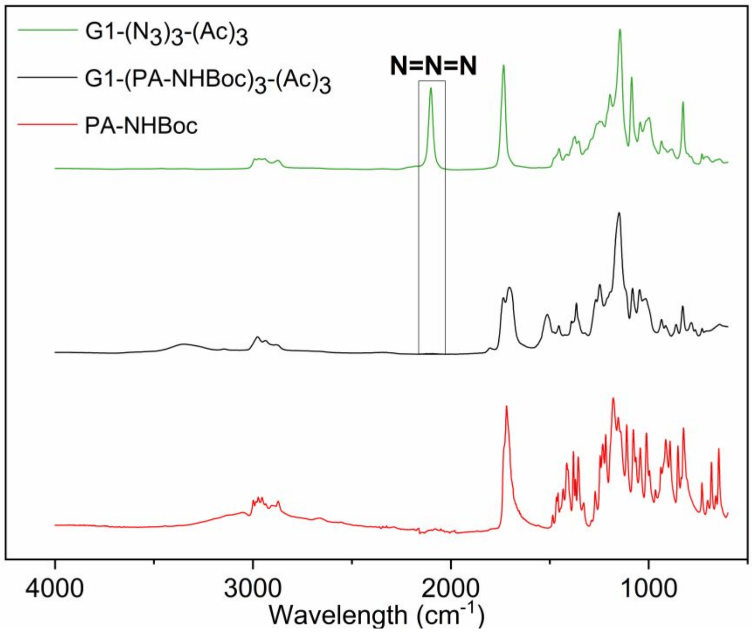

Figure S17. Stacked FTIR spectra of G1-(N_3)$_3$-(Ac)$_3$ (Top), G1-(PA-NHBoc)$_3$-(Ac)$_3$ (Middle) and free PA-NHBoc (Bottom).

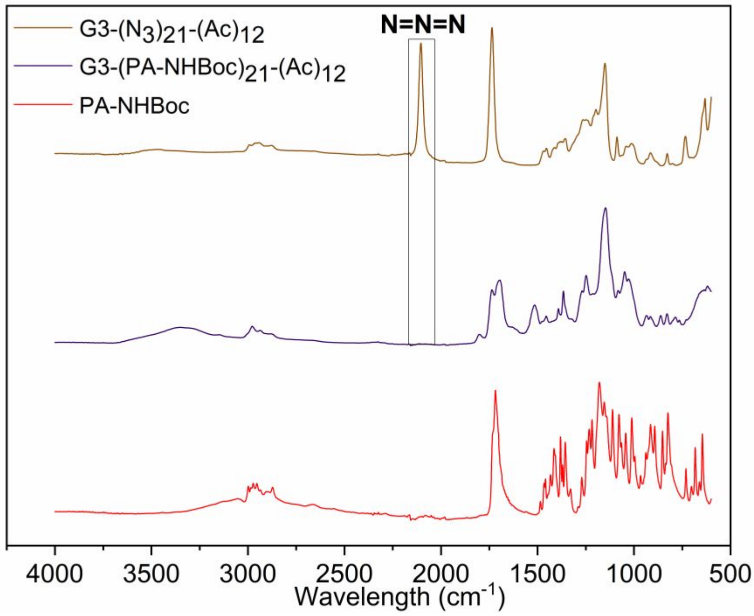

Figure S18. Stacked FTIR spectra of G3-(N_3)$_{21}$-(Ac)$_{12}$ (Top), G3-(PA-NHBoc)$_{21}$-(Ac)$_{12}$ (Middle) and free PA-NHBoc (Bottom).

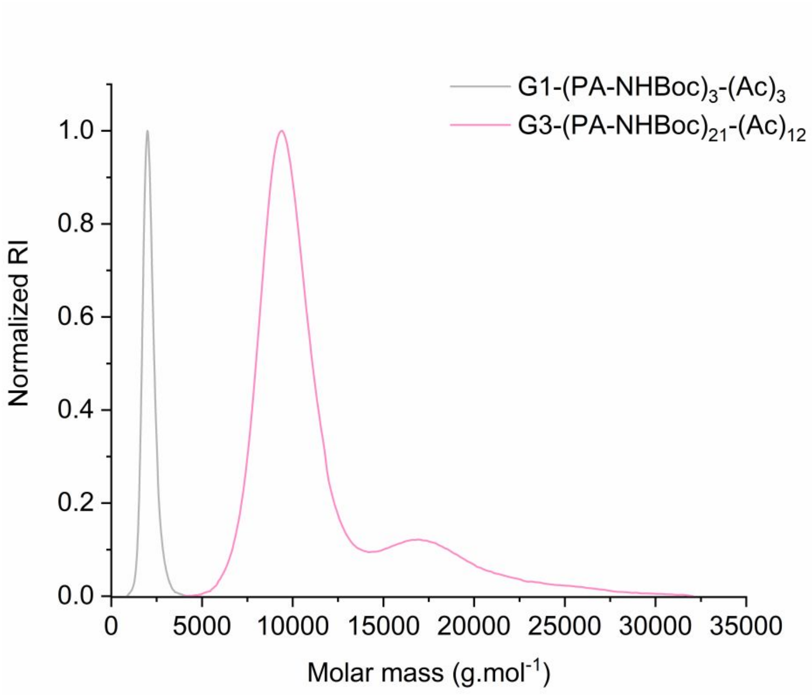

Figure S19. SEC overlay of G1-(PA-NHBoc)$_3$-(Ac)$_3$ and G3-(PA-NHBoc)$_{21}$-(Ac)$_{12}$.

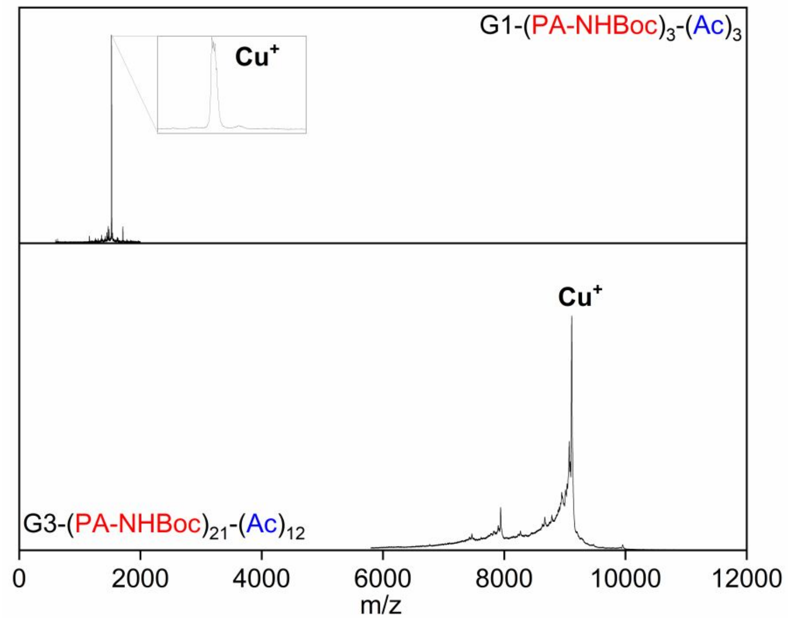

Figure S20. Stacked MALDI-TOF spectra of G1-(PA-NHBoc)$_3$-(Ac)$_3$ and G3-(PA-NHBoc)$_{21}$-(Ac)$_{12}$ in DCTB.

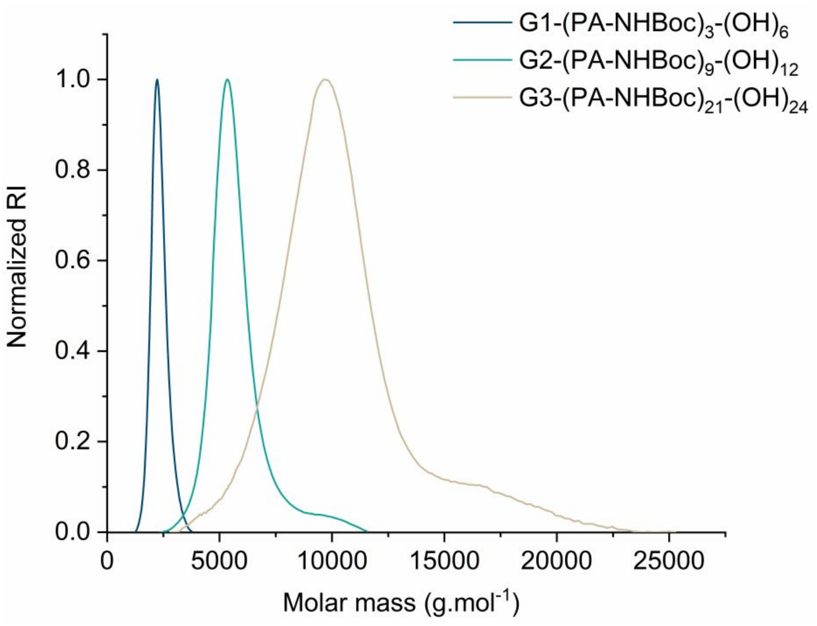

Figure S21. SEC overlay of G1-(PA-NHBoc)$_3$-(OH)$_6$, G2-(PA-NHBoc)$_9$-(OH)$_{12}$ and G3-(PA-NHBoc)$_{21}$-(OH)$_{24}$.

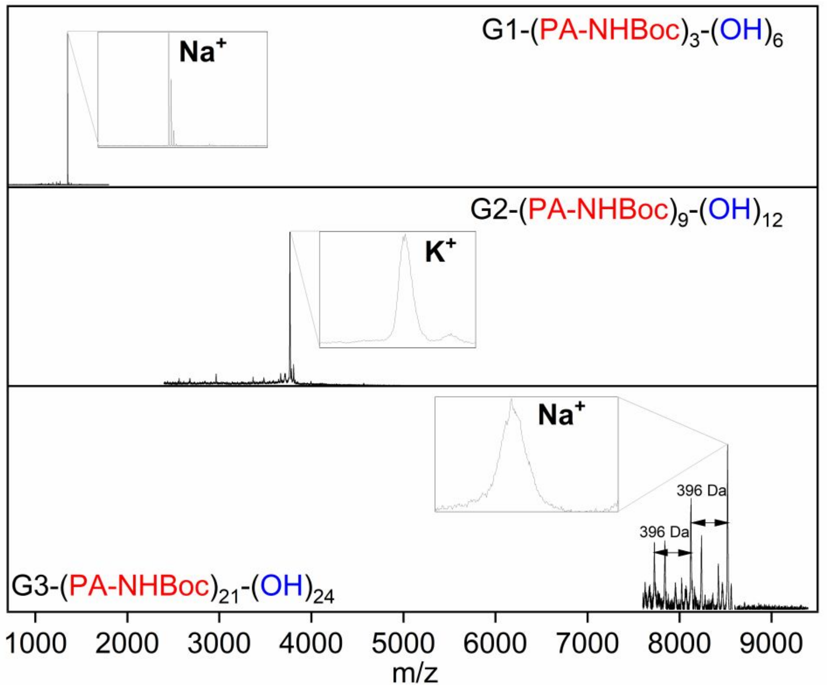

Figure S22. Stacked MALDI-TOF spectra of G1-(PA-NHBoc)$_3$-(OH)$_6$, G2-(PA-NHBoc)$_9$-(OH)$_{12}$ and G3-(PA-NHBoc)$_{21}$-(OH)$_{24}$ in DCTB. The MALDI-TOF spectrum of G3-(PA-NHBoc)$_{21}$-(OH)$_{24}$ indicated multiple peaks with a difference of 396 Da. The observed phenomenon is attributed to the cleavage of ester linkages within the AB_2C monomer units of the dendrimer upon laser irradiation, as well as potential limitations in detection sensitivity when using the DCTB matrix in MALDI-TOF analysis.

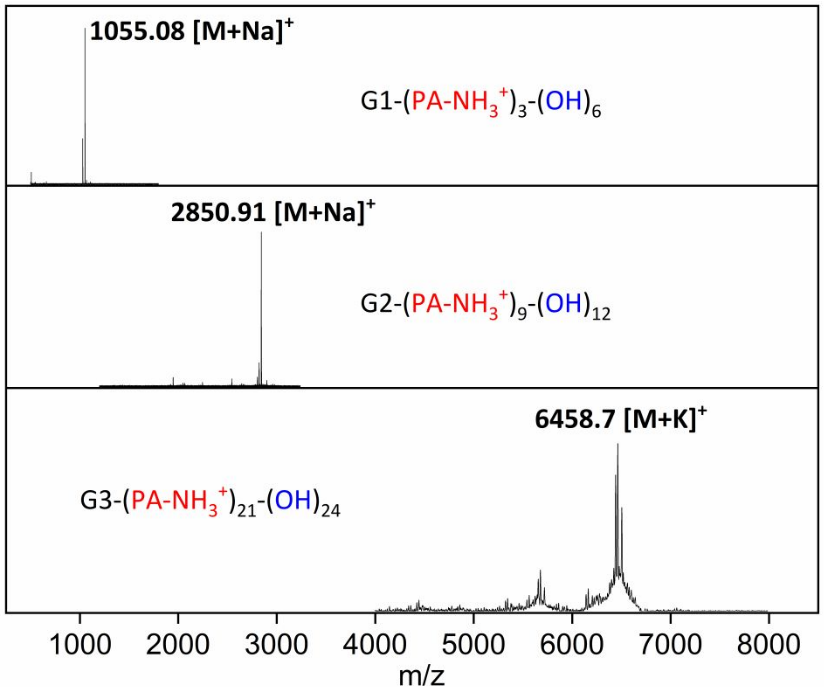

Figure S23. Stacked MALDI-TOF spectra of G1-(PA-NH_3^+)$_3$-(OH)$_6$, G2-(PA-NH_3^+)$_9$-(OH)$_{12}$ and G3-(PA-NH_3^+)$_{21}$-(OH)$_{24}$ in DHB.

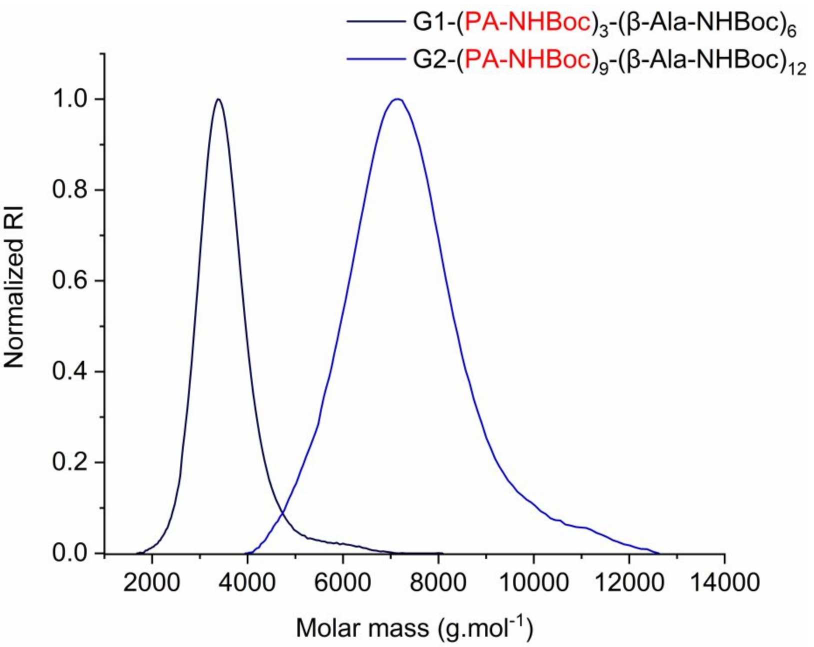

Figure S24. SEC overlay of G1-(PA-NHBoc)$_3$-(β-Ala-NHBoc)$_6$ and G2-(PA-NHBoc)$_9$-(β-Ala-NHBoc)$_{12.}$

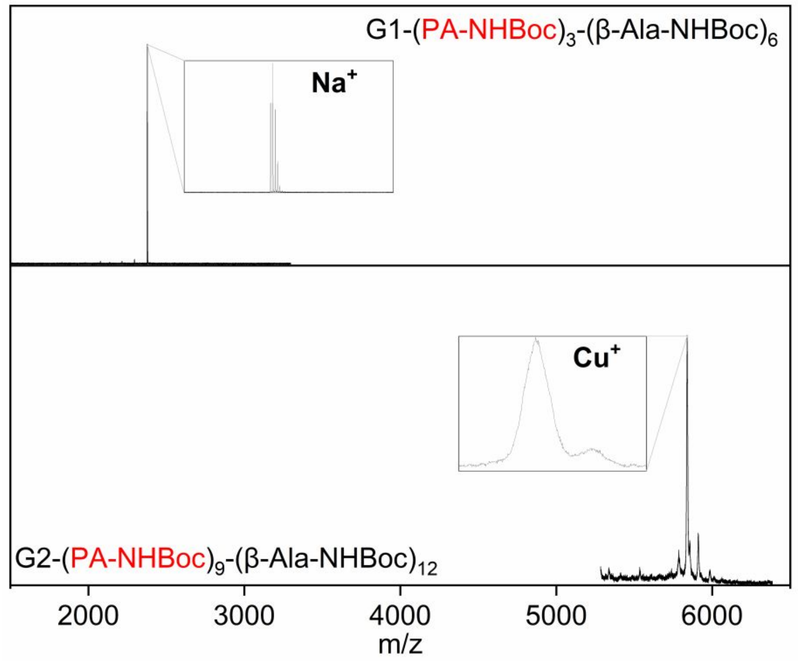

Figure S25. Stacked MALDI-TOF spectra of G1-(PA-NHBoc)$_3$-(β-Ala-NHBoc)$_6$ and G2-(PA-NHBoc)$_9$-(β-Ala-NHBoc)$_{12}$ in DCTB.

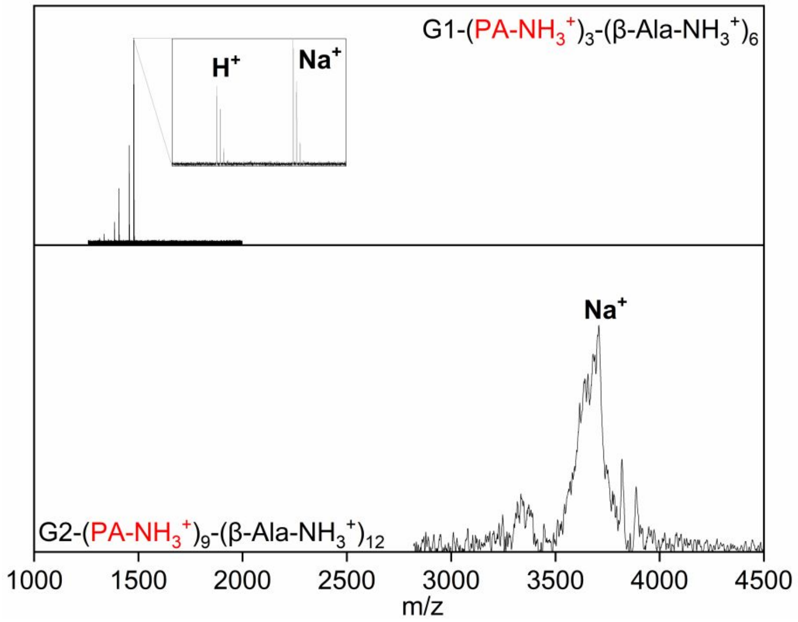

Figure S26. Stacked MALDI-TOF spectra of G1-(PA-NH_3^+)$_3$-(β-Ala-NH_3^+)$_6$ in DHB and G2-(PA-NH_3^+)$_9$-(β-Ala-NH_3^+)$_{12}$ in DCTB.

References

1. Singh, A.; Hutchinson, D. J.; Montañez, M. I.; Sanz Del Olmo, N.; Malkoch, M. Synthesis, Evaluation, and Modification of Heterofunctional Polyester Dendrimers with Internally Queued Bromide Groups. *Soft Matter* **2024**, *20* (38), 7573–7577.
2. Stenström, P.; Hjorth, E.; Zhang, Y.; Andrén, O. C. J.; Guette-Marquet, S.; Schultzberg, M.; Malkoch, M. Synthesis and *In Vitro* Evaluation of Monodisperse Amino-Functional Polyester Dendrimers with Rapid Degradability and Antibacterial Properties. *Biomacromolecules* **2017**, *18* (12), 4323–4330.
3. Fan, Y.; Namata, F.; Erlandsson, J.; Zhang, Y.; Wågberg, L.; Malkoch, M. Self-Assembled Polyester Dendrimer/Cellulose Nanofibril Hydrogels with Extraordinary Antibacterial Activity. *Pharmaceutics* **2020**, *12* (12), 1139.
